# Supplementary material for: Cohesin‐mediated DNA loop extrusion resolves sister chromatids in G2 phase
Source: EMBO J. 2023 Jun 26;42(16):e113475. doi: 10.15252/embj.2023113475 (PMC10425840; doi:10.15252/embj.2023113475)
Supplement: Supplementary file 1 — Appendix S1 [file EMBJ-42-e113475-s002.pdf]

Appendix

Cohesin-mediated DNA loop extrusion resolves sister chromatids in G2 phase

Paul Batty[1,2#], Christoph C.H. Langer[1], Zsuzsanna Takács[1], Wen Tang[3], Claudia Blaukopf[1], Jan-Michael Peters[3], Daniel W. Gerlich[1#]

[1]Institute of Molecular Biotechnology of the Austrian Academy of Sciences (IMBA), Vienna BioCenter (VBC), 1030 Vienna, Austria
[2]Vienna BioCenter PhD Program, Doctoral School of the University of Vienna and Medical University of Vienna, A-1030, Vienna, Austria
[3]Research Institute of Molecular Pathology (IMP), Vienna BioCenter (VBC), Vienna, 1030 Vienna, Austria

Table of Contents

Appendix Figures 2

Appendix Figure S1: Validation of protein depletion efficiency in WAPL-dTAG and SMC4-AID/WAPL-dTAG cells 2

Appendix Figure S2: Cohesin axes split upon WAPL depletion in Sororin depleted G2 cells 4

Appendix Figure S3: Acute depletion of condensin and WAPL in prometaphase cells 6

Appendix Figure S4: Validation of cell cycle stage and protein depletion efficiency for scsHi-C 7

Appendix Figure S5: Uncropped Western blot images 9

Appendix Figure S6: Flow cytometry gating strategy 11

Appendix Tables 12

Appendix Table S1: Flow cytometry sample statistics for G2 scsHi-C samples 12

Appendix Table S2: Flow cytometry sample statistics for prometaphase scsHi-C samples 12

Appendix Table S3: Cell lines used in this study 13

Appendix Table S4: scsHi-C read statistics for ΔNIPBL G2 cells 13

Appendix Table S5: scsHi-C read statistics for ΔSMC4 G2 cells 14

Appendix Table S6: scsHi-C read statistics for ΔWAPL G2 cells 14

Appendix Table S7: scsHi-C read statistics for ΔWAPL ΔSororin G2 cells 14

Appendix Table S8: scsHi-C read statistics for wild type prometaphase cells 14

Appendix Table S9: scsHi-C read statistics for ΔNIPBL prometaphase cells 14

Appendix Table S10: scsHi-C read statistics for ΔSMC4 prometaphase cells 14

Appendix Table S11: Published datasets used in this study 15

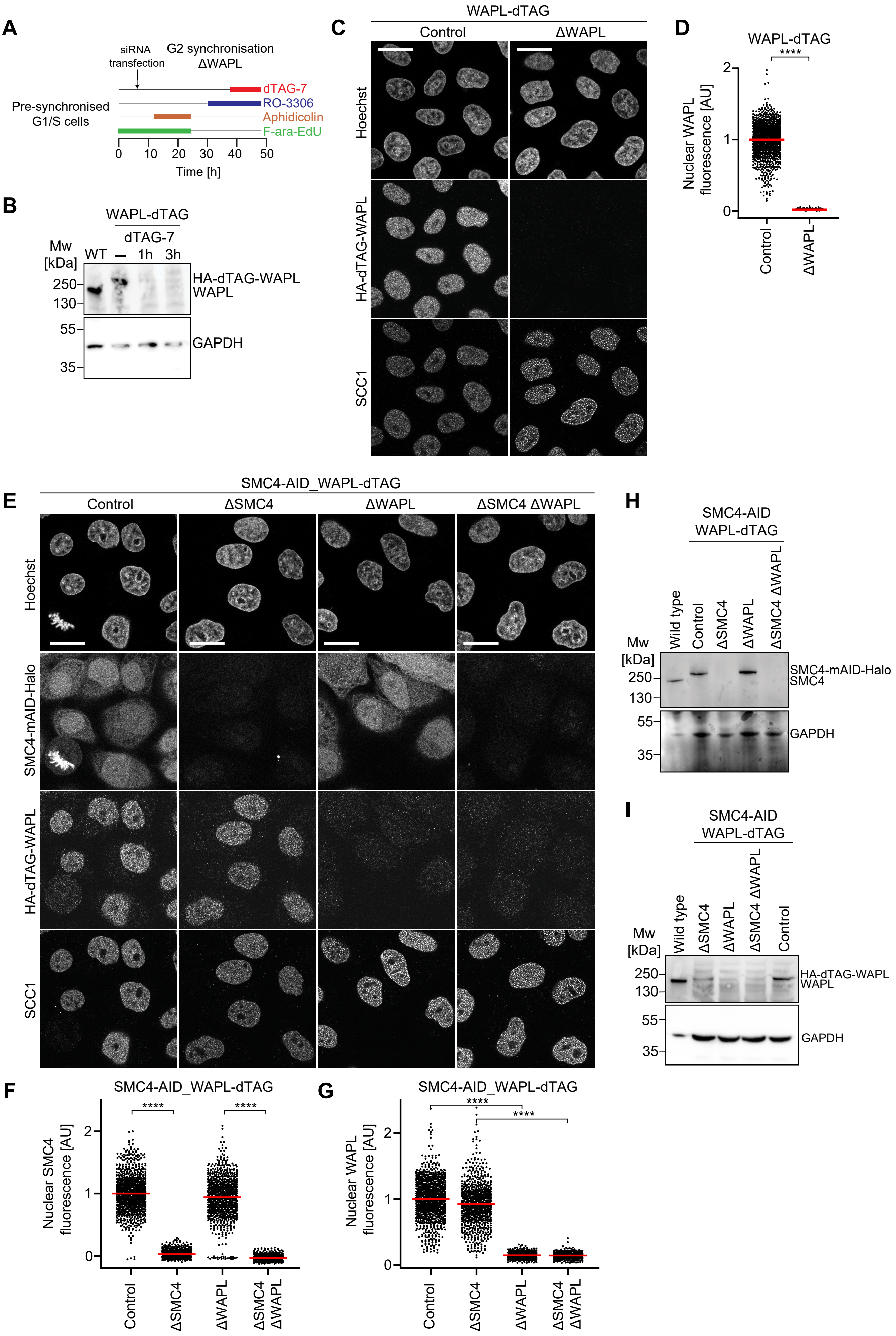

Appendix Figure S1 | Validation of protein depletion efficiency in WAPL-dTAG and SMC4-AID_WAPL-dTAG cells.
Legend on next page.

Appendix Figure S1 | Validation of protein depletion efficiency in WAPL-dTAG and SMC4-AID_WAPL-dTAG cells.
A, Schematic of experimental procedure for generation of one-sister labelled chromatids in ΔWAPL G2 cells. Cells were treated with the requisite compounds as indicated; the end point indicates the time of fixation. For conditions where WAPL was not depleted, dTAG-7 was not added to the cells.
B, Immunoblot analysis of WAPL in wild type (WT) cells, untreated WAPL-dTAG cells and WAPL-dTAG cells treated for either 1 h or 3 h with dTAG-7. Representative example of n = 2 biological replicates. For source gel data, see Appendix Fig. S5D.
C, Immunofluorescence analysis of HeLa cells homozygously tagged for HA-dTAG-WAPL. Cells were incubated for 3 h with (ΔWAPL) or without (Control) 1 μM dTAG-7 before subsequently fixing and staining for HA-WAPL and SCC1, with anti-HA and anti-SCC1 antibodies respectively. DNA was stained with Hoechst 33342.
D, Quantification of mean nuclear HA-WAPL fluorescence per cell, as shown in C. Dots represent individual cells; red bars indicate the mean. For each condition two experimental replicates were performed. n = 1466 cells analysed for control WAPL-dTAG cells, n = 1372 cells analysed for ΔWAPL cells. Significance was tested using a two-tailed Mann Whitney U test; P < 10-324 (precision limit of floating-point arithmetic).
E, Immunofluorescence analysis of HeLa cells homozygously tagged for SMC4-mAID-Halo and HA-dTAG-WAPL, and stably expressing OsTIR1F74G. Cells were incubated with 5-Ph-IAA or dTAG-7 in the following combinations: untreated (Control), 3 h 5-Ph-IAA (ΔSMC4), 3 h dTAG-7 (ΔWAPL), 3 h 5-Ph-IAA + dTAG-7 (ΔSMC4 ΔWAPL). SMC4 was stained with TMR-HaloTag ligand and HA-WAPL and SCC1 were stained using antibodies as in **B**. DNA was stained with Hoechst 33342.
F, Quantification of mean nuclear SMC4 fluorescence per cell, as shown in E. Dots represent individual cells; red bars indicate the mean. Wild type cells were stained with Halo-TMR and the mean Halo-TMR fluorescence within the segmented nuclei then calculated. Normalisation was performed relative to the mean nuclear Halo-TMR fluorescence of wild type cells (0 value), and control SMC4-AID_WAPL-dTAG cells (1 value). For each condition two experimental replicates were performed. n = 1007 cells analysed for control SMC4-AID_WAPL-dTAG cells, n = 848 cells analysed for ΔSMC4 cells, n = 867 cells analysed for ΔWAPL cells, n = 717 cells analysed for ΔSMC4 ΔWAPL cells. Significance was tested using a two-tailed Mann Whitney U test; P = 1.07 x 10-297 (ΔSMC4), P = 3.81 x 10-244 (ΔSMC4 ΔWAPL).
G, Quantification of mean nuclear HA-WAPL fluorescence per cell, as shown in **E**. Sample numbers as in **F.** Dots represent individual cells; red bars indicate the mean. Significance was tested using a two-tailed Mann Whitney U test; P = = 6.36 x 10-305 (ΔWAPL), P = 5.15 x 10-253 (ΔSMC4 ΔWAPL).
H, Immunoblot analysis of SMC4 in wild type and SMC4-AID_WAPL-dTAG cells treated as follows: untreated (Control), 3 h 5-Ph-IAA (ΔSMC4), 3 h dTAG-7 (ΔWAPL), 3 h 5-Ph-IAA + dTAG-7 (ΔSMC4 ΔWAPL). Representative example of n = 2 biological replicates. For source gel data, see Appendix Fig. S5E, F.
I, Immunoblot analysis of WAPL in wild type and SMC4-AID_WAPL-dTAG cells treated as follows: untreated (Control), 3 h 5-Ph-IAA (ΔSMC4), 3 h dTAG-7 (ΔWAPL), 3 h 5-Ph-IAA + dTAG-7 (ΔSMC4 ΔWAPL). Representative example of n = 2 biological replicates. For source gel data, see Appendix Fig. S5G.
Data information: (****) P < 0.0001; two-tailed Mann Whitney U test. Biological replicates: **B** (n = 2), **H, I** (n = 2). Technical replicates: **C-G** (n = 2). All microscopy images are single Z-sections. Scale bars: 20 μm.

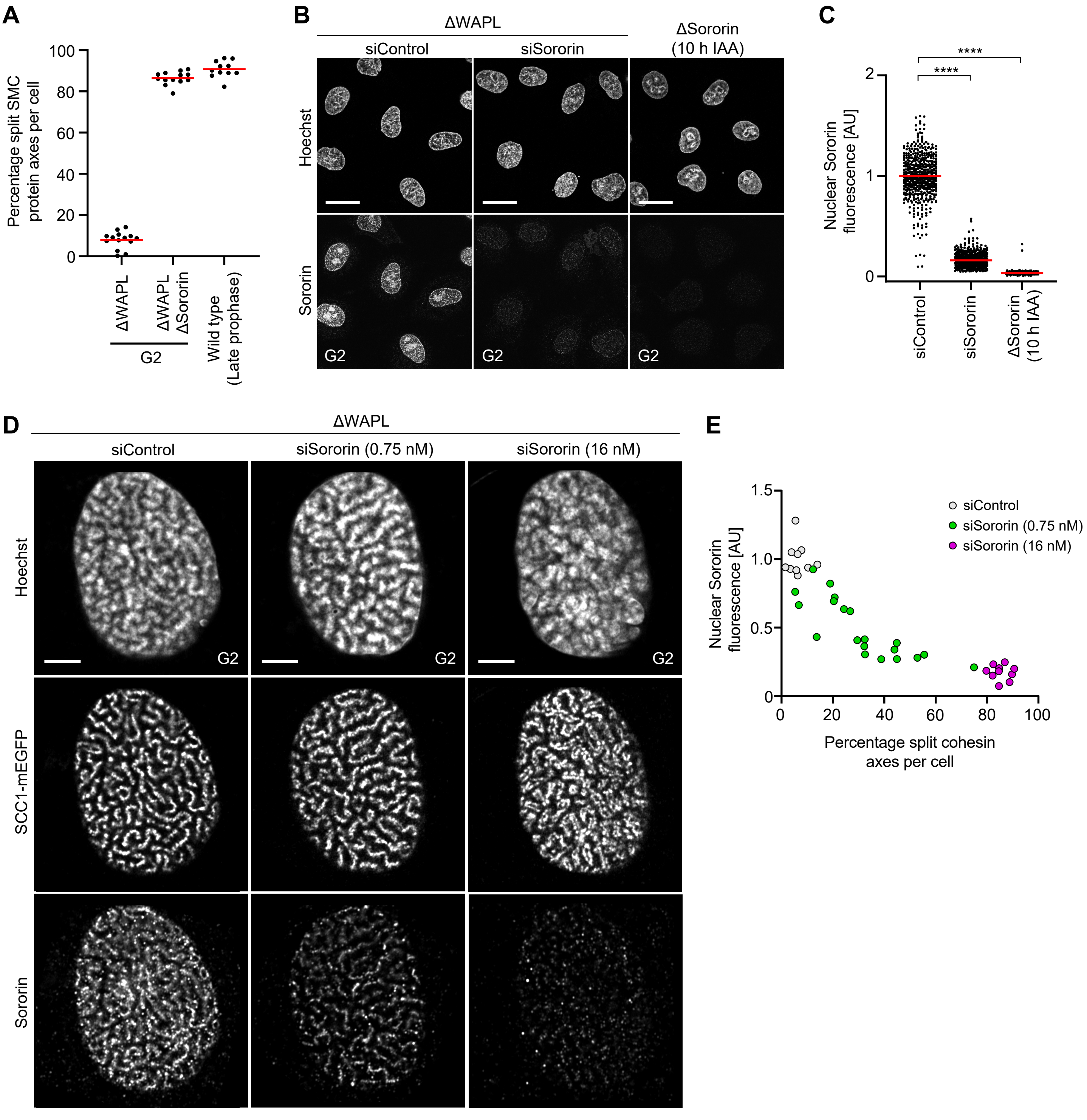

Appendix Figure S2 | Cohesin axes split upon WAPL depletion in Sororin depleted G2 cells.

A, Scatter plot of the percentage of split cohesin (SCC1) axes in ΔWAPL G2 and ΔWAPL ΔSororin G2 cells, and the percentage of split condensin (SMC4) axes in wild type late prophase cells, as indicated. Line profiles were drawn along cohesin or condensin axes and the percentage of split axis segments along the total axis length then calculated on a per cell basis. ΔWAPL G2 cells (n = 230 lines from 14 cells from 6 replicates), ΔWAPL ΔSororin G2 cells (n = 299 lines from 13 cells from 4 replicates), and wild type late prophase cells (n = 101 lines from 11 cells from 5 replicates) were analysed. Dots represent individual cells; red bars indicate the mean.

B, Immunofluorescence analysis of HeLa cells homozygously tagged for Halo-mAID-WAPL (ΔWAPL) or Sororin-mAID-mEGFP (Δ Sororin) as indicated. Cells were synchronised to G2 phase by RO-3306. WAPL and Sororin were depleted through the addition of 500 µM auxin (IAA) for 10 h. ΔWAPL cells were treated with 16 nM Control (siControl) or Sororin (siSororin) siRNAs as indicated. Sororin was stained using an anti-Sororin antibody, DNA was stained using Hoechst 33342.

C, Quantification of mean nuclear Sororin fluorescence per cell, as shown in **B**. Dots represent individual cells; red bars indicate the mean. For each condition two experimental replicates were performed. n = 545 cells analysed for siControl, n = 990 cells analysed for siSororin, n = 971 cells analysed for ΔSororin. Significance was tested using a two-tailed Mann Whitney U test; P = 9.48 x 10-226 (siSororin), P = 3.77 x 10-231 (ΔSororin).

Legend continues on next page.

D, Immunofluorescence of ΔWAPL G2 cells to assess the localisation and abundance of Sororin and the extent of cohesin axis splitting upon siRNA-mediated depletion of Sororin. Cells were treated with Control (siControl) or Sororin (siSororin) siRNAs as indicated. Control siRNAs were used at 16 nM and Sororin siRNAs were used at 0.75 nM or 16 nM, as indicated. WAPL was depleted in G2 phase. SCC1 was homozygously tagged with mEGFP and was visualised using an anti-EGFP nanobody, Sororin was stained using an anti-Sororin antibody, DNA was stained using Hoechst 33342. To aid visualisation the SCC1-mEGFP channel is not contrast matched.
E, Scatter plot of the percentage of split cohesin (SCC1-mEGFP) axes against mean nuclear Sororin fluorescence on a per cell basis for ΔWAPL G2 cells treated with siRNAs as in **D**. Quantification of the percentage of split cohesin axes was performed as in Appendix Fig. S2A. Quantification of mean nuclear Sororin fluorescence was performed for central Z-stack slices. Dots represent individual cells. For each condition two experimental replicates were performed. n = 135 lines from 10 cells from 2 replicates analysed for ΔWAPL siControl, n = 233 lines from 10 cells from 2 replicates analysed for ΔWAPL siSororin (16 nM), n = 441 lines from 20 cells from 2 replicates analysed for ΔWAPL siSororin (0.75 nM).
Data information: (****) P < 0.0001; two-tailed Mann Whitney U test. Biological replicates: **B-E** (n = 2). Images of fields are single Z-sections from Z-stack images. Images of single cells are single Z-slices from 3D-stacks. Scale bar fields: 30 μm, scale bars single cells: 5 μm.

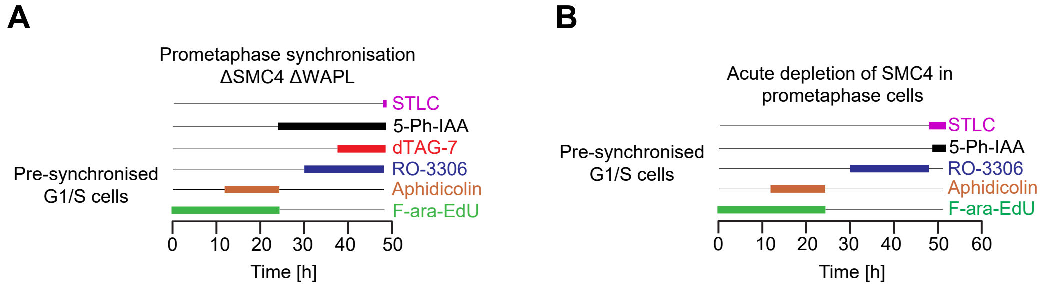

Appendix Figure S3 | Acute depletion of condensin and WAPL in prometaphase cells.

A, Schematic of experimental procedure for generation of one-sister labelled chromatids in ΔSMC4 ΔWAPL prometaphase cells, to test if cohesin is able to compensate for sister chromatid resolution defects resulting from condensin depletion. SMC4 was depleted in G1 through the addition of 5-Ph-IAA 1 h before the final release into S phase such that cells progressed through S and G2 phase in the absence of condensins. WAPL was depleted in G2 phase after completion of DNA replication to avoid potential effects of WAPL depletion on cohesion establishment. Cells were treated with the requisite compounds as indicated; the end point indicates the time of fixation.

B, Schematic of experimental procedure for generation of one-sister labelled chromatids in control prometaphase cells, before acute depletion of SMC4 through the addition of 5-Ph-IAA for 120 or 240 min. Schematic represents depletion of SMC4 for 240 min.

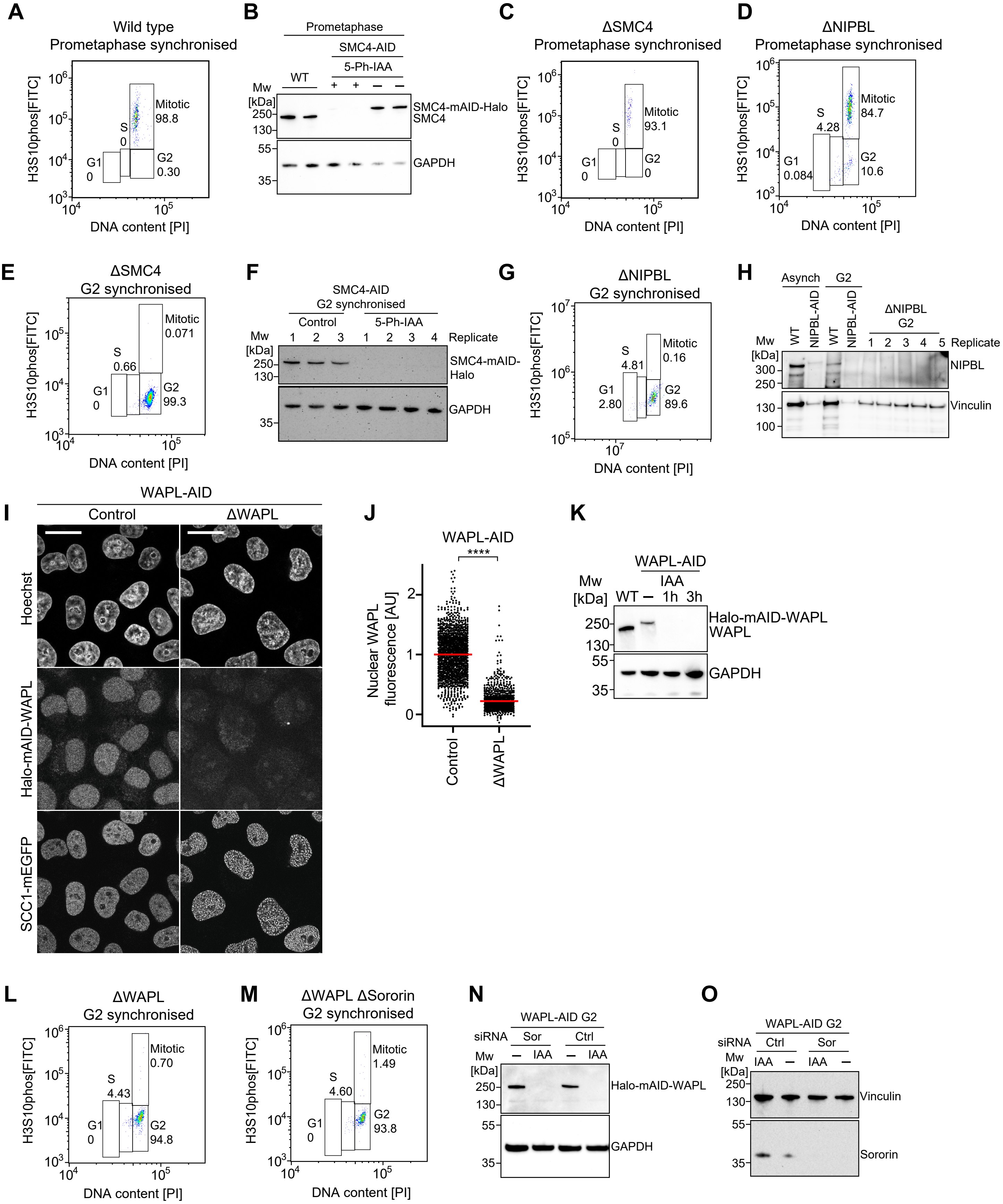

Appendix Figure S4 | Validation of cell cycle stage and protein depletion efficiency for scsHi-C.
Legend on next page.

Appendix Figure S4 | Validation of cell cycle stage and protein depletion efficiency for scsHi-C.
A-M, Cell cycle analysis by flow cytometry (A, C, D, E, G, L, M). Cells were stained with propidium iodide to determine DNA content and an antibody against phospho-H3-Ser10 as a marker for mitotic cells. The gates indicate the cell cycle stage, and the numbers indicate the percentage of cells measured for a given cell cycle stage.
A, Representative flow cytometry plot for one of the wild type prometaphase samples (n = 2 biological replicates).
B, Immunoblot analysis of SMC4 in wild type (WT) and SMC4-AID prometaphase cells harvested for scsHi-C analysis, plus or minus 5-Ph-IAA as indicated. For source gel data, see Appendix Fig. S5I.
C, Representative flow cytometry plot for one of the ΔSMC4 prometaphase samples (n = 2 biological replicates).
D, Representative flow cytometry plot for one of the ΔNIPBL prometaphase samples (n = 3 biological replicates).
E, Representative flow cytometry plot for one of the ΔSMC4 G2 samples (n = 4 biological replicates).
F, Immunoblot analysis of G2 synchronised control SMC4-AID and ΔSMC4 cells harvested for scsHi-C analysis, plus or minus 5-Ph-IAA as indicated. For source gel data, see Appendix Fig. S5J.
G, Representative flow cytometry plot for one of the ΔNIPBL G2 samples (n = 10 biological replicates).
H, Immunoblot analysis of NIPBL in wild type (WT) and G2 synchronised NIPBL-AID cells harvested for downstream scsHi-C analysis. For source gel data, see Appendix Fig. S5K.
I, Immunofluorescence analysis of HeLa cells homozygously tagged for Halo-mAID-WAPL and stably expressing OsTIR1. Cells were incubated for 2 h with (ΔWAPL) or without (Control) 500 µM auxin before subsequently staining of WAPL with TMR-HaloTag ligand. SCC1-mEGFP was stained with an anti-SCC1 antibody or an anti-mEGFP nanobody and DNA was stained with Hoechst 33342. Images show single Z-sections.
J, Quantification of mean nuclear WAPL fluorescence per cell, as shown in **I**. Wild type cells were stained with Halo-TMR and the mean Halo-TMR fluorescence within the segmented nuclei then calculated. Normalisation was performed relative to the mean nuclear Halo-TMR fluorescence of wild type cells (0 value) and control WAPL-AID cells (1 value). n = 1537 cells analysed for control WAPL-AID cells, n = 1199 cells analysed for ΔWAPL cells. Significance was tested using a two-tailed Mann Whitney U test; P = < 10-324 (precision limit of floating-point arithmetic).
K, Immunoblot analysis of WAPL in wild type cells, untreated WAPL-AID cells and WAPL-AID cells treated for either 1 h or 3 h with auxin (IAA). Representative example of n = 3 biological replicates. For source gel data, see Appendix Fig. S5L.
L, Representative flow cytometry plot for one of the ΔWAPL G2 samples (n = 6 biological replicates).
M, Representative flow cytometry plot for one of the ΔWAPL ΔSororin G2 samples (n = 4 biological replicates).
N, Immunoblot analysis of WAPL in WAPL-AID cells harvested for scsHi-C analysis, treated with Control (Ctrl) or Sororin (Sor) siRNAs, plus or minus auxin (IAA), as indicated. For source gel data, see Appendix Fig. S5M.
O, Immunoblot analysis of Sororin in WAPL-AID cells harvested for scsHi-C analysis, treated with Control (Ctrl) or Sororin (Sor) siRNAs, plus or minus auxin (IAA) as indicated. For source gel data, see Appendix Fig. S5N.
Data information: (****) P < 0.0001; two-tailed Mann Whitney U test. Biological replicates: **A-H**, **K-O**: as in Fig. 5 and Fig. EV6, **I, J** (n = 2). Technical replicates: I, J (n = 3).

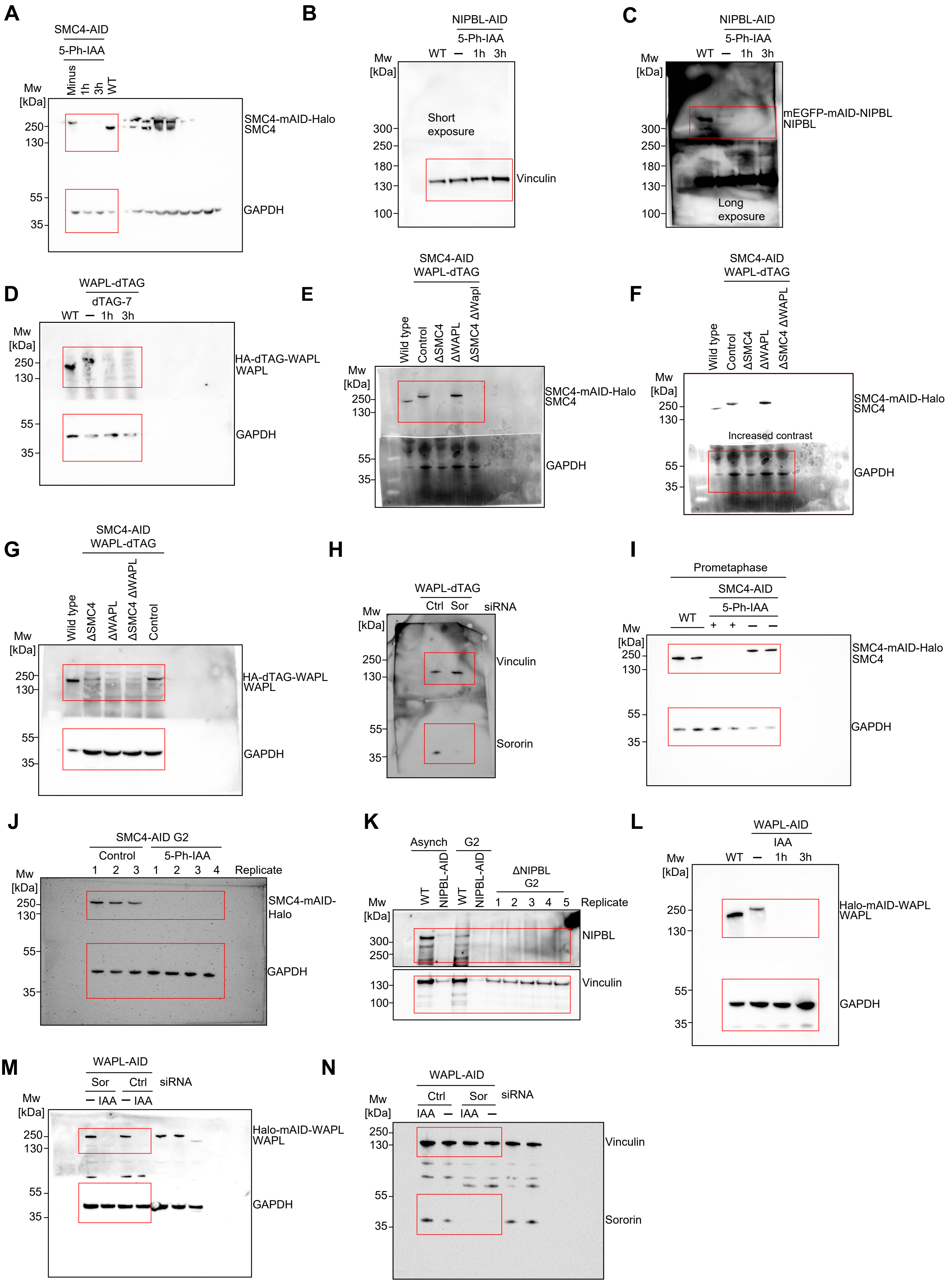

Appendix Figure S5 | Uncropped Western blot images.
Legend on next page.

Appendix Figure S5 | Uncropped Western blot images.

Loading controls were run on the same gel as the target protein, and unless otherwise specified, the membrane was cut at 70 KDa after transfer to allow detection of different proteins. In all cases, the cropped images used in the requisite figure panels are indicated with red boxes.

A, Characterisation of SMC4-AID cells. Uncropped blot of wild type (WT) and SMC4-AID cells (untreated, 1 h 5-Ph-IAA, 3 h 5-Ph-IAA), as indicated, blotting against SMC4 and GAPDH. Cropped images shown in Fig. EV3B.

B, Characterisation of NIPBL-AID cells. Uncropped blot of wild type (WT) and NIPBL-AID cells (untreated, 1 h 5-Ph-IAA, 3 h 5-Ph-IAA, as indicated), short exposure time, showing the loading control (Vinculin). Cropped area shown in Fig. EV4B. Loading controls were run on the same gel and the membrane cut after transfer at 250 KDa to stain NIPBL and Vinculin with different antibodies

C, Uncropped blot as in **B**, long exposure time, blotting against NIPBL.

D, Characterisation of WAPL-dTAG cells. Uncropped blot of wild type (WT) and WAPL-dTAG cells (untreated, 1 h dTAG-7, 3 h dTAG-7, as indicated). Cropped area shown in Appendix Fig. S1B, blotting against WAPL and GAPDH.

E, Characterisation of SMC4-AID_WAPL-dTag cells. Uncropped blot of wild type and SMC4-AID_WAPL-dTAG cells (untreated, 3 h 5-Ph-IAA, 3 h dTAG-7, 3 h 5-Ph-IAA + dTAG-7, as indicated), showing blotting against SMC4. Cropped area shown in Appendix Fig. S1H, blotting against SMC4 and GAPDH.

F, Uncropped blot as in **E**, increased contrast, showing blotting against the loading control (GAPDH).

G, Characterisation of SMC4-AID_WAPL-dTAG cells. Same conditions as in **E, F,** blotting against WAPL and GAPDH. Cropped areas shown in Appendix Fig. S1I, blotting against WAPL and GAPDH.

H, Uncropped blot of WAPL-dTAG cells treated with Control (Ctrl) or Sororin (Sor) siRNAs, as indicated. Cropped areas shown in Fig. EV5F, blotting against Sororin and Vinculin.

I, Uncropped blot of prometaphase synchronised wild type (WT) and SMC4-AID cells harvested for scsHi-C analysis, plus or minus 5-Ph-IAA as indicated. Cropped areas shown in Appendix Fig. S4B, blotting against SMC4 and GAPDH.

J, Uncropped blot of G2 synchronised control SMC4-AID and ΔSMC4 cells harvested for scsHi-C analysis, plus or minus 5-Ph-IAA as indicated. Cropped areas shown in Appendix Fig. S4F, blotting against SMC4 and GAPDH.

K, Uncropped blot of wild type (WT) and G2 synchronised NIPBL-AID cells harvested for scsHi-C analysis. Cropped areas shown in Appendix Fig. S4H, blotting against NIPBL and Vinculin.

L, Characterisation of WAPL-AID cell line. Uncropped blot of wild type (WT) and WAPL-AID cells (untreated, 1 h auxin (IAA), 3 h auxin (IAA), as indicated). Cropped area shown in Appendix Fig. S4K, blotting against WAPL and GAPDH.

M, Uncropped blot of G2 synchronised WAPL-AID cells harvested for scsHi-C analysis, treated with Control (Ctrl) or Sororin (Sor) siRNAs, plus or minus auxin (IAA), as indicated. Cropped areas shown in Appendix Fig. S4N, blotting against WAPL and GAPDH.

N, Uncropped blot of G2 synchronised WAPL-AID cells harvested for scsHi-C analysis, treated with Control (Ctrl) or Sororin (Sor) siRNAs, plus or minus auxin (IAA), as indicated. Cropped areas shown in Appendix Fig. S4O, blotting against Sororin and Vinculin.

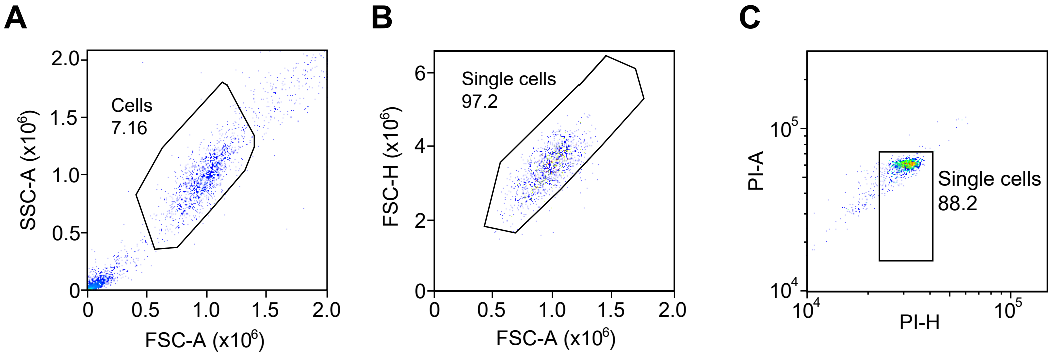

Appendix Figure S6 | Flow cytometry gating strategy.
For all panels, numbers indicate the percentage of cells inside the gate. Cells were stained with propidium iodide to determine DNA content and an antibody against phospho-H3-Ser10 as a marker for mitotic cells.
A, FSC-A vs SSC-A gate to gate cells and remove debris, shown for one of the G2 synchronised ΔWAPL samples.
B, FSC-A vs FSC-H gate to identify single cells.
C, PI-H vs PI-A, second gate to identify single cells. Cells in this gate were subsequently used to analyse cell cycle stage in Appendix Fig. S4L. The same gating strategy was used for Appendix Fig. S4A, C, D, E, G, M.

Appendix Tables

Appendix Table S1: Flow cytometry sample statistics for G2 scsHi-C samples

Condition	Replicate	#G1	#S	#G2	#M	% G1	% S	% G2	%M
ΔWAPL G2	Replicate 1	87	57	1151	9	6.64	4.35	87.9	0.69
ΔWAPL G2	Replicate 2	4	5	260	1	1.48	1.85	95.9	0.37
ΔWAPL G2	Replicate 3	0	23	329	1	0	6.53	93.5	0.28
ΔWAPL G2	Replicate 4	0	51	1095	8	0	4.42	94.8	0.69
ΔWAPL G2	Replicate 5	1	107	1632	12	0	6.1	93	0.68
ΔWAPL G2	Replicate 6	0	53	613	9	0	7.83	90.5	1.33
ΔWAPL ΔSororin G2	Replicate 1	5	7	430	1	1.13	1.58	97.1	0.23
ΔWAPL ΔSororin G2	Replicate 2	0	74	541	4	0	11.9	87.1	0.64
ΔWAPL ΔSororin G2	Replicate 3	0	90	992	2	0	8.29	91.3	0.18
ΔWAPL ΔSororin G2	Replicate 4	0	91	594	8	0	13.1	85.3	1.15
ΔSMC4 G2	Replicate 1	0	14	1264	0	0	1.1	99.1	0
ΔSMC4 G2	Replicate 2	0	17	1317	0	0	1.28	98.9	0
ΔSMC4 G2	Replicate 3	0	7	1009	0	0	0.69	99.3	0
ΔSMC4 G2	Replicate 4	0	28	4195	3	0	0.66	99.3	0.071
ΔNIPBL G2	Replicate 1	18	31	577	1	2.8	4.81	89.6	0.16
ΔNIPBL G2	Replicate 2	17	56	661	2	2.25	7.41	87.4	0.26
ΔNIPBL G2	Replicate 3	18	50	518	1	3.01	8.35	86.5	0.17
ΔNIPBL G2	Replicate 4	20	67	665	1	2.6	8.7	86.4	0.13
ΔNIPBL G2	Replicate 5	18	65	816	2	1.97	7.12	89.4	0.22
ΔNIPBL G2	Replicate 6	52	112	1172	1	3.81	8.2	85.8	0.073
ΔNIPBL G2	Replicate 7	60	148	1466	0	3.5	8.64	85.6	0
ΔNIPBL G2	Replicate 8	43	83	874	0	4.2	8.1	85.3	0
ΔNIPBL G2	Replicate 9	36	87	889	0	3.44	8.32	85	0
ΔNIPBL G2	Replicate 10	96	136	1533	0	5.33	7.55	85.1	0

Appendix Table S2: Flow cytometry sample statistics for prometaphase scsHi-C samples

Condition	Replicate	#G1	#S	#G2	#M	% G1	% S	% G2	%M
WT prometaphase	Replicate 1	0	4	5	150	0	2.44	3.05	91.05
WT prometaphase	Replicate 2	0	0	2	666	0	0	0.3	98.8
ΔSMC4 prometaphase	Replicate 1	0	0	2	115	0	0	1.64	94.3
ΔSMC4 prometaphase	Replicate 2	0	0	0	147	0	0	0	93.1
ΔNIPBL prometaphase	Replicate 1	1	52	126	1010	0.084	4.36	10.6	84.7
ΔNIPBL prometaphase	Replicate 2	0	30	87	556	0	4.44	12.9	82.2
ΔNIPBL prometaphase	Replicate 3	0	52	82	707	0	6.09	10.3	82.8

Appendix Table S3: Cell lines used in this study

**Name**	**Genotype**	**Plasmids used**	**Resistance marker**	**Internal Lab ID**	**Already published or generated for this study**
HeLa Kyoto	Wild type	n/a	n/a	1	
HeLa Kyoto Sororin-AID	EGFP-AID-Sororin TIR1-3xMyc-T2A-Puro (Lentiviral integration)	EGFP-AID-Sororin repair template<br>TIR1-3xMyc-T2A PuroLentivirus<br>sgRNA expressing / Cas9^{D10A} expressing to tag Sororin	Puromycin	1712	Published in Mitter et al; 2020
HeLa Kyoto SMC4-AID	SMC4-mAID-Halo OsTIR1(F74G)-SNAP-IRES-Blast (integrated into AAVS1 safe harbour locus)	SMC4-mAID-Halo repair template<br>OsTIR1^{F74G}-SNAP-IRES-Blast repair template<br>sgRNA expressing/Cas9-human geminin fusion (Cas9-hGem) to tag SMC4<br>sgRNA / Cas9-human geminin fusion (Cas9-hGem) expressing to integrate OsTIR1^{F74G}	Blasticidin S<br>Puromycin	2056	Published in Schneider et al; 2022
HeLa Kyoto NIPBL-AID	mEGFP-mAID-NIPBL OsTIR1(F74G)—SNAP-IRES-Blast (integrated into AAVS1 safe harbour locus)	mEGFP-mAID-NIPBL repair template<br>OsTIR1^{F74G}-SNAP-IRES-Blast repair template<br>sgRNA / Cas9-human geminin fusion (Cas9-hGem) expressing to tag NIPBL<br>sgRNA / Cas9-human geminin fusion (Cas9-hGem) expressing to integrate OsTIR1^{F74G}	Blasticidin S	2045	Generated for this study
HeLa Kyoto WAPL-AID	Halo-mAID-WAPL SCC1-mEGFP TIR1-3xMyc-T2A-Puro (Lentiviral integration)	Halo-mAID-WAPL repair template<br>SCC1-mEGFP repair template<br>TIR1-3xMyc-T2A PuroLentivirus<br>sgRNA expressing / Cas9^{D10A} expressing to tag WAPL	Puromycin	1802	Generated for this study
HeLa Kyoto WAPL-FKBP12^{F36V} (WAPL-dTAG)	Blasticidin-P2A-2xHA-FKBP12^{F36V}(dTAG) -WAPL	Blasticidin-P2A-2xHA-FKBP12^{F36V} repair template<br>sgRNA / Cas9^{D10A} expressing to tag WAPL	Blasticidin S	2096	Generated for this study
HeLa Kyoto SMC4-AID_WAPL FKBP12^{F36V} (WAPL-dTAG)	SMC4-mAID-Halo Blasticidin-P2A-2xHA-FKBP12^{F36V}(dTAG) -WAPL OsTIR1^{F74G}-SNAP-IRES-Blast (integrated into AAVS1 safe harbour locus)	SMC4-mAID-Halo repair template<br>HygromycinB-P2A-2xHA-FKBP12^{F36V} repair template<br>OsTIR1^{F74G}-SNAP-IRES-Blast repair template<br>sgRNA / Cas9-human geminin fusion (Cas9-hGem) expressing to tag SMC4<br>sgRNA / Cas9-human geminin fusion (Cas9-hGem) expressing to integrate OsTIR1^{F74G}	Blasticidin S<br>Hygromycin B<br>Puromycin	2108	Generated for this study, base cell line HeLa Kyoto SMC4-AID cell line mentioned above

Appendix Table S4: scsHi-C read statistics for ΔNIPBL G2 cells

**Replicate**	**Total**	**Mapped**	**Unique**	**Cis**	**Trans Heterologue**	**Cis 1Kb+**	**Cis 10Kb+**	**Cis sister**	**Trans sister**
Replicate 1	6.09E+07	5.00E+07	4.18E+07	3.41E+07	7.68E+06	2.57E+07	1.94E+07	2.08E+06	4.79E+05
Replicate 2	7.93E+07	6.30E+07	3.53E+07	2.85E+07	6.85E+06	2.13E+07	1.58E+07	1.51E+06	3.49E+05
Replicate 3	7.36E+07	5.98E+07	5.06E+07	4.14E+07	9.28E+06	3.08E+07	2.27E+07	2.47E+06	5.56E+05
Replicate 4	8.93E+07	7.22E+07	5.79E+07	4.74E+07	1.04E+07	3.53E+07	2.58E+07	2.56E+06	5.60E+05
Replicate 5	6.55E+07	5.37E+07	4.28E+07	3.49E+07	7.92E+06	2.61E+07	1.93E+07	2.00E+06	4.59E+05
Replicate 6	7.04E+07	5.74E+07	4.39E+07	3.59E+07	8.00E+06	2.60E+07	1.95E+07	9.43E+05	1.92E+05
Replicate 7	6.51E+07	5.19E+07	4.06E+07	3.27E+07	7.82E+06	2.35E+07	1.78E+07	6.74E+05	1.29E+05
Replicate 8	6.14E+07	5.01E+07	4.49E+07	3.60E+07	8.89E+06	2.62E+07	2.00E+07	1.28E+06	2.77E+05
Replicate 9	7.01E+07	5.57E+07	4.84E+07	3.81E+07	1.03E+07	2.76E+07	2.10E+07	1.39E+06	3.00E+05
Replicate 10	5.72E+07	4.61E+07	4.09E+07	3.35E+07	7.43E+06	2.23E+07	1.66E+07	7.86E+05	1.28E+05

Appendix Table S5: scsHi-C read statistics for ΔSMC4 G2 cells

Replicate	Total	Mapped	Unique	Cis	Trans Heterologue	Cis 1Kb+	Cis 10Kb+	Cis sister	Trans sister
Replicate 1	1.34E+08	1.02E+08	2.99E+07	2.60E+07	3.89E+06	1.38E+07	1.00E+07	1.88E+06	1.53E+05
Replicate 2	1.36E+08	1.10E+08	5.32E+07	1.10E+08	5.66E+06	2.52E+07	1.77E+07	3.83E+06	2.82E+05
Replicate 3	1.29E+08	1.01E+08	4.95E+07	4.35E+07	5.99E+06	2.36E+07	1.70E+07	4.34E+06	3.63E+05
Replicate 4	1.09E+08	7.26E+07	3.72E+07	2.52E+07	3.03E+06	1.54E+07	1.18E+07	3.32E+06	2.53E+05

Appendix Table S6: scsHi-C read statistics for ΔWAPL G2 cells

Replicate	Total	Mapped	Unique	Cis	Trans Heterologue	Cis 1Kb+	Cis 10Kb+	Cis sister	Trans sister
Replicate 1	3.50E+07	2.68E+07	1.64E+07	1.42E+07	2.25E+06	9.84E+06	7.64E+06	8.09E+05	1.06E+05
Replicate 2	4.64E+07	3.12E+07	9.40E+06	7.85E+06	1.55E+06	4.84E+06	3.80E+06	4.66E+05	5.27E+04
Replicate 3	5.62E+07	3.13E+07	1.15E+07	9.77E+06	1.70E+06	6.46E+06	5.02E+06	5.31E+05	6.16E+04
Replicate 4	7.99E+07	6.17E+07	1.02E+07	9.09E+06	1.11E+06	6.07E+06	4.54E+06	6.95E+05	9.36E+04
Replicate 5	1.00E+08	7.03E+07	1.92E+07	1.69E+07	2.32E+06	1.24E+07	9.86E+06	9.28E+05	1.53E+05
Replicate 6	9.30E+07	7.01E+07	8.50E+06	7.41E+06	1.09E+06	4.91E+06	3.78E+06	4.92E+05	6.75E+04

Appendix Table S7: scsHi-C read statistics for ΔWAPL ΔSororin G2 cells

Replicate	Total	Mapped	Unique	Cis	Trans Heterologue	Cis 1Kb+	Cis 10Kb+	Cis sister	Trans sister
Replicate 1	4.67E+07	3.52E+07	1.38E+07	1.17E+07	2.11E+06	7.45E+06	5.59E+06	7.30E+05	7.20E+04
Replicate 2	3.74E+07	2.77E+07	1.06E+07	8.99E+06	1.57E+06	6.33E+06	4.69E+06	4.08E+05	4.28E+04
Replicate 3	3.71E+07	2.73E+07	1.12E+07	9.14E+06	2.05E+06	6.27E+06	4.84E+06	5.50E+05	6.52E+04
Replicate 4	1.09E+08	7.26E+07	2.82E+07	2.52E+07	3.03E+06	1.54E+07	1.18E+07	1.95E+06	1.58E+05

Appendix Table S8: scsHi-C read statistics for wild type prometaphase cells

Replicate	Total	Mapped	Unique	Cis	Trans Heterologue	Cis 1Kb+	Cis 10Kb+	Cis sister	Trans sister
Replicate 1	7.88E+07	5.89E+07	3.12E+07	2.84E+07	2.87E+06	2.39E+07	2.09E+07	9.76E+05	8.35E+04
Replicate 2	6.53E+07	4.90E+07	2.22E+07	2.01E+07	2.08E+06	1.67E+07	1.44E+07	5.39E+05	5.17E+04

Appendix Table S9: scsHi-C read statistics for ΔNIPBL prometaphase cells

Replicate	Total	Mapped	Unique	Cis	Trans Heterologue	Cis 1Kb+	Cis 10Kb+	Cis sister	Trans sister
Replicate 1	3.65E+07	2.85E+07	7.66E+06	6.48E+06	1.18E+06	3.80E+06	3.15E+06	2.49E+05	2.47E+04
Replicate 2	4.20E+07	3.09E+07	1.45E+07	1.28E+07	1.76E+06	9.02E+06	7.69E+06	5.03E+05	4.62E+04
Replicate 3	2.78E+07	2.08E+07	6.71E+06	5.61E+06	1.10E+06	4.33E+06	3.74E+06	1.74E+05	2.11E+04

Appendix Table S10: scsHi-C read statistics for ΔSMC4 prometaphase cells

Replicate	Total	Mapped	Unique	Cis	Trans Heterologue	Cis 1Kb+	Cis 10Kb+	Cis sister	Trans sister
Replicate 1	7.56E+07	5.40E+07	3.54E+07	2.82E+07	7.24E+06	2.36E+07	2.00E+07	1.38E+06	4.72E+05
Replicate 2	7.38E+07	5.46E+07	4.34E+07	3.72E+07	6.14E+06	2.75E+07	2.18E+07	1.96E+06	3.79E+05

Appendix Table S11: Published datasets used in this study

Name	GEO ID	Description
Hi-C: G2	GSM4613674	Cooler files for wild type HeLa Kyoto cells, synchronised to G2 (All contacts, cis sister contacts, trans sister contacts)
Hi-C: Sororin-AID + auxin G2	GSM4613677	Cooler files for Sororin depleted HeLa Kyoto cells, synchronised to G2 (All contacts, cis sister contacts, trans sister contacts)
