# Supplementary material for: Cohesin‐mediated DNA loop extrusion resolves sister chromatids in G2 phase
Source: EMBO J. 2023 Jun 26;42(16):e113475. doi: 10.15252/embj.2023113475 (PMC10425840; doi:10.15252/embj.2023113475)
Supplement: Supplementary file 2 — Expanded View Figures PDF [file EMBJ-42-e113475-s008.pdf]

Expanded View Figures

Figure EV1. Validation of sister chromatid resolution assay.

A Schematic of experimental procedure for generation of two or one-sister labelled chromatids in wild-type cells. Cells were treated with the requisite compounds as indicated.

B Bar plot indicating the percentage of chromosome segments with 0, 1 or 2 labelled sister chromatids for wild-type prometaphase cells, fixed during the first mitosis after labelling with F-ara-EdU. n = 121 sister chromatid pairs from 15 cells from seven replicates analysed. Bar indicates mean; error bars indicate standard deviation.

C Bar plot indicating the percentage of chromosome segments with 0, 1 or 2 labelled sister chromatids for wild-type prometaphase cells, fixed during the second mitosis after labelling with F-ara-EdU. n = 129 sister chromatid pairs from 27 cells from seven replicates analysed. Bar indicates mean; error bars indicate standard deviation.

D Schematic indicating the image analysis pipeline to calculate the sister chromatid separation score. Extract central Z-sections: The central slice of an input Z-stack is calculated, and the five slices above and below are chosen (11 slices in total). Segment chromatin, extract pixel intensities: The chromatin channel is segmented to generate a mask, which is applied to the Hoechst and F-ara-EdU channels. Pixel intensities within the mask are extracted. Pixel correlation analysis: The Spearman correlation coefficient (SCC) between Hoechst and F-ara-EdU pixel values in the mask is calculated first per slice, and then per cell. See Materials and Methods for details of the normalisation procedure to generate the separation score. Plot is an example scatter plot representation of pixel value intensities for Hoechst and F-ara-EdU fluorescence in a prometaphase cell labelled on one sister chromatid.

E Scatter plot representation and SCC of pixel value intensities for Hoechst and F-ara-EdU fluorescence for a single slice of a wild-type prometaphase cell, fixed during the 1st mitosis after labelling with F-ara-EdU (two-sister labelled). Real data is shown.

F Scatter plot representation and SCC of pixel value intensities for Hoechst and F-ara-EdU fluorescence for a single slice of a wild-type prometaphase cell, fixed during the 2nd mitosis after labelling with F-ara-EdU (one-sister labelled). Real data is shown.

G Quantification of the Spearman correlation coefficient between Hoechst and F-ara-EdU for wild-type prometaphase chromosomes fixed during the 1st (two-sister labelled) or 2nd (one-sister labelled) mitosis after labelling with F-ara-EdU, as indicated. Individual data points represent the mean SCC per cell, red bars indicate the mean. n = 59 cells (1st mitosis), n = 83 cells (2nd mitosis). Significance was tested using a two-tailed Mann–Whitney U test; $P = 3.91 \times 10^{-24}$.

H Quantification of sister chromatid separation as in Fig 1D and E, for wild-type prometaphase chromosomes fixed during the 1st (two-sister labelled) or 2nd (one-sister labelled) mitosis after labelling with F-ara-EdU. Dots represent individual cells; red bars indicate the mean. Sample numbers and statistics as in (G). Quantification for one-sister labelled cells is the same as in Fig 1D (wild-type) to allow side by side comparison with two-sister labelled prometaphase cells.

I Quantification of sister chromatid separation as in Fig 1D and E. Two-sister labelled (n = 44 cells) and one-sister sister labelled (n = 69 cells) wild-type cells synchronised to G2 by RO-3306 were analysed. Dots represent individual cells; red bars indicate the mean. Significance was tested using a two-tailed Mann–Whitney U test; $P = 4.06 \times 10^{-19}$. Quantification for one-sister labelled G2 cells is the same as in Fig 1E (wild-type) to allow side by side comparison with two-sister labelled G2 cells.

Data information: (****) $P < 0.0001$; two-tailed Mann–Whitney U test. Biological and technical replicates: (G–I) Two-sister and one-sister labelled wild-type prometaphase and G2 cells, as in Fig 1.

© 2023 The Authors

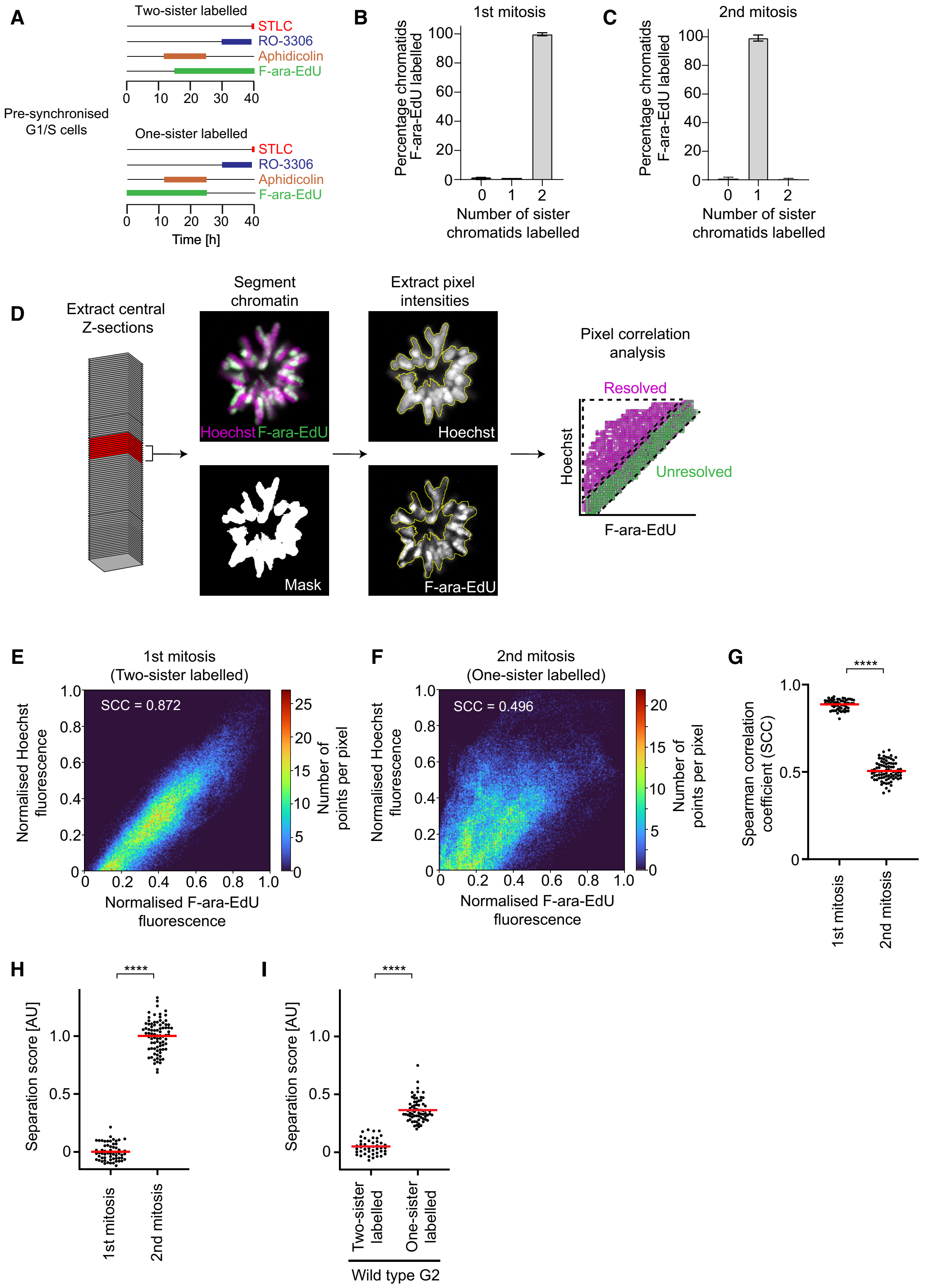

Figure EV1.

 © 2023 The Authors

Figure EV2. Validation of cell cycle stage for sister chromatid labelling.

Cells were F-ara-EdU labelled on one sister chromatid and synchronised to different cell cycle stages as in Figs 1 and 2 to determine the abundance and localisation of the mitotic histone modification phospho-H3-Ser10 and cyclin B1 by immunofluorescence. Prometaphase and prophase cells were treated with STLC, G2 cells were treated with RO-3306.

A Representative images for one-sister labelled sister chromatids in cells stained with an anti-phospho-H3-Ser10 antibody from wild-type prometaphase, prophase and G2 cells as indicated.

B Quantification of mean phospho-H3-Ser10 fluorescence within the chromatin mask for central Z-slices for the conditions shown in (A). Prometaphase (n = 48 cells), prophase (n = 32 cells), and G2 (n = 31 cells) cells were analysed. Dots represent individual cells; red bars indicate the mean. Significance was tested using a two-tailed Mann–Whitney U test; $P = 9.61 \times 10^{-12}$ (prophase), $P = 8.33 \times 10^{-14}$ (prometaphase).

C Representative images for one-sister labelled sister chromatids in cells stained with an anti-cyclin B1 antibody, from wild-type prometaphase, prophase and G2 arrested cells as indicated.

D Quantification of mean cyclin B1 fluorescence within the chromatin mask for central Z-slices for the conditions shown in (C). Calculation of mean fluorescence was performed as described in (B). Prometaphase (n = 48 cells), prophase (n = 32 cells), and G2 (n = 69 cells) cells were analysed. Dots represent individual cells; red bars indicate the mean. Significance was tested using a two-tailed Mann–Whitney U test; $P = 7.95 \times 10^{-16}$ (prophase).

E Representative images for one-sister labelled sister chromatids from G2 cells, treated with RO-3306 or untreated, as indicated.

F Quantification of sister chromatid separation as in Fig 1D and E for one-sister labelled wild-type G2 cells, for the conditions shown in (E). RO-3306 treated (n = 69 cells) and untreated (n = 16 cells) cells were analysed. Dots represent individual cells; red bars indicate the mean. Quantification for RO-3306 treated cells is the same as in Fig 1E (wild-type) to allow side by side comparison with untreated cells. A different example RO-3306 treated cell is shown.

Data information: (****) $P < 0.0001$; two-tailed Mann–Whitney U test. Biological replicates: (A–F) One-sister labelled wild-type G2 (RO-3306 treated) as in Fig 1. Technical replicates: (A–D) Wild-type prometaphase (n = 3), wild-type prophase (n = 4). (E, F) Wild-type G2 (untreated) (n = 2). All images shown are single Z-slices from 3D-stacks. Scale bars: 5 µm.

© 2023 The Authors

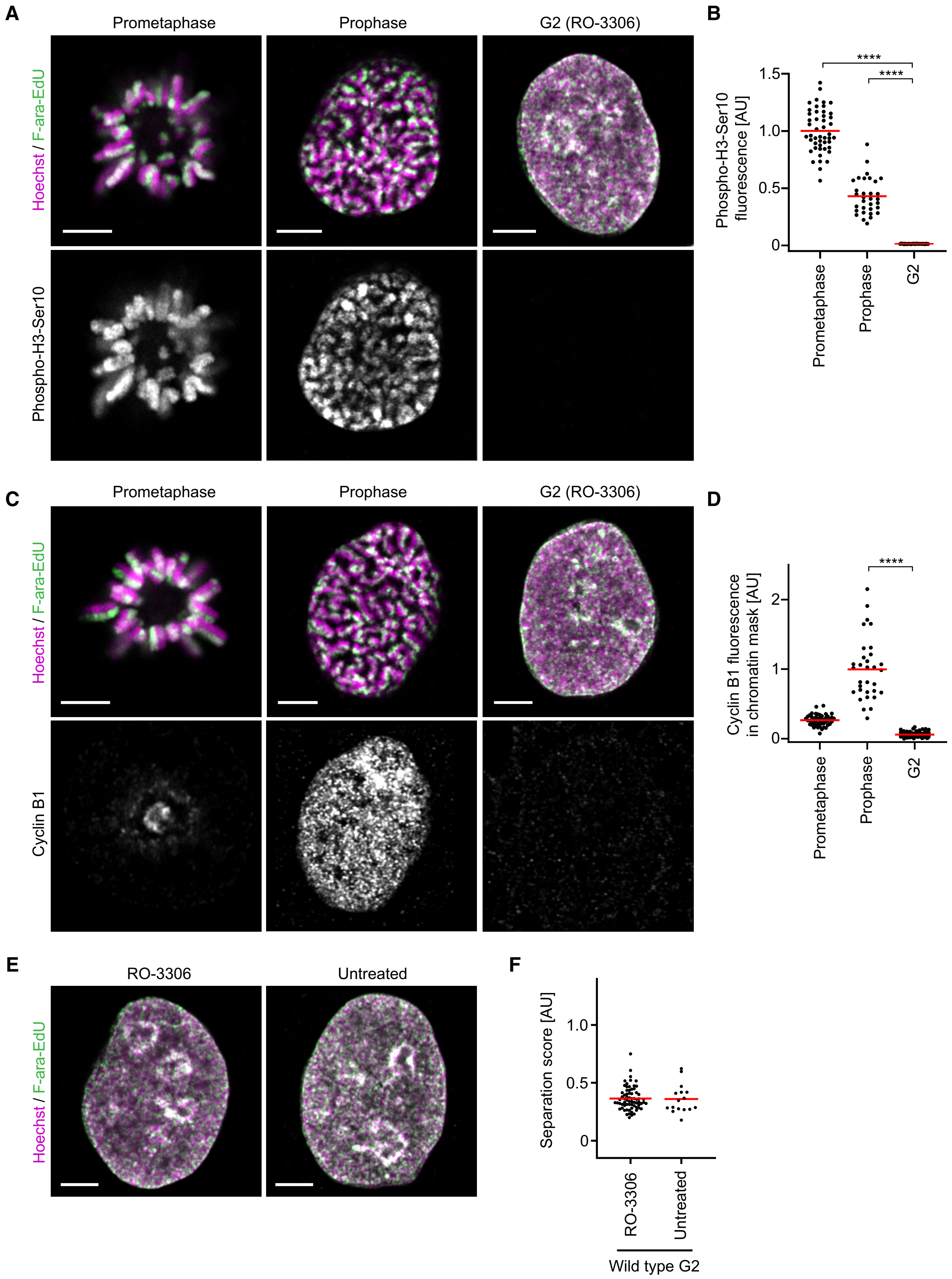

Figure EV2.

 © 2023 The Authors

Figure EV3. Validation of protein depletion efficiency and sister labelling controls in SMC4-AID cells.

A Schematic of experimental procedure for generation of one-sister labelled chromatids in ΔSMC4 G2 cells. Cells were treated with the requisite compounds as indicated; the end point indicates the time of fixation. Prometaphase samples were generated by washing out RO-3306 and releasing into medium containing STLC.

B Immunoblot analysis of SMC4 in wild-type (WT) cells, untreated SMC4-AID cells and SMC4-AID cells treated for either 1 or 3 h with 5-Ph-IAA. Representative example of n = 3 biological replicates.

C Immunofluorescence analysis of HeLa cells homozygously tagged for SMC4-mAID-Halo and stably expressing OsTIR1^{F74G}. Cells were incubated for 3 h with (ΔSMC4) or without (Control) 1 μM 5-Ph-IAA before subsequently staining of SMC4 with HaloTag TMR Ligand. DNA was stained with Hoechst 33342.

D Quantification of mean nuclear SMC4 fluorescence per cell, as shown in (C). Dots represent individual cells; red bars indicate the mean. Wild-type cells were stained with HaloTag TMR Ligand and the mean Halo-TMR fluorescence within the segmented nuclei then calculated. Normalisation was performed relative to the mean nuclear Halo-TMR fluorescence of wild-type cells (0 value) and control SMC4-AID cells (1 value). For each condition two experimental replicates were performed. n = 1,159 cells analysed for control SMC4-AID cells, n = 1,000 cells analysed for ΔSMC4 cells. Significance was tested using a two-tailed Mann–Whitney U test; $P < 10^{-324}$ (precision limit of floating-point arithmetic).

E Representative images from ΔSMC4 prometaphase cells labelled on one or two sister chromatids as indicated.

F Representative images from ΔSMC4 G2 cells labelled on one or two sister chromatids as indicated.

G Quantification of sister chromatid separation as in Fig 1D and E, for ΔSMC4 prometaphase cells labelled on one or two sister chromatids. Dots represent individual cells; red bars indicate the mean. Two-sister labelled (n = 26 cells) and one-sister labelled (n = 75 cells) cells were analysed. Quantification for one-sister labelled ΔSMC4 prometaphase cells is the same as in Fig 1D (ΔSMC4) to allow side by side comparison with two-sister labelled cells. A different example prometaphase cell labelled on one sister chromatid is shown.

H Quantification of sister chromatid separation as in Fig 1D and E, for ΔSMC4 G2 cells labelled on one or two sister chromatids. Dots represent individual cells; red bars indicate the mean. Two-sister labelled (n = 19 cells) and one-sister labelled (n = 50 cells) cells were analysed. Quantification for one-sister labelled ΔSMC4 G2 cells is the same as in Fig 1E (ΔSMC4) to allow side by side comparison with two-sister labelled cells. A different example G2 cell labelled on one sister chromatid is shown.

Data information: (****) $P < 0.0001$; two-tailed Mann–Whitney U test. Biological replicates: B (n = 3), (E–H), ΔSMC4 two-sister labelled prometaphase (n = 4), ΔSMC4 two-sister labelled G2 (n = 3), ΔSMC4 one-sister labelled prometaphase and G2 as in Fig 1. Technical replicates: C, D (n = 2), (E-H) ΔSMC4 two-sister labelled prometaphase (n = 9), ΔSMC4 two-sister labelled G2 (n = 5), ΔSMC4 one-sister labelled prometaphase and G2 as in Fig 1. Images with fields of cells (panel C) are single Z-sections. Images with single cells (panels E, F) are single Z-slices from 3D-stacks. Scale bars fields: 20 μm. Scale bars single cells, large panels: 5 μm, insets: 1 μm.

© 2023 The Authors

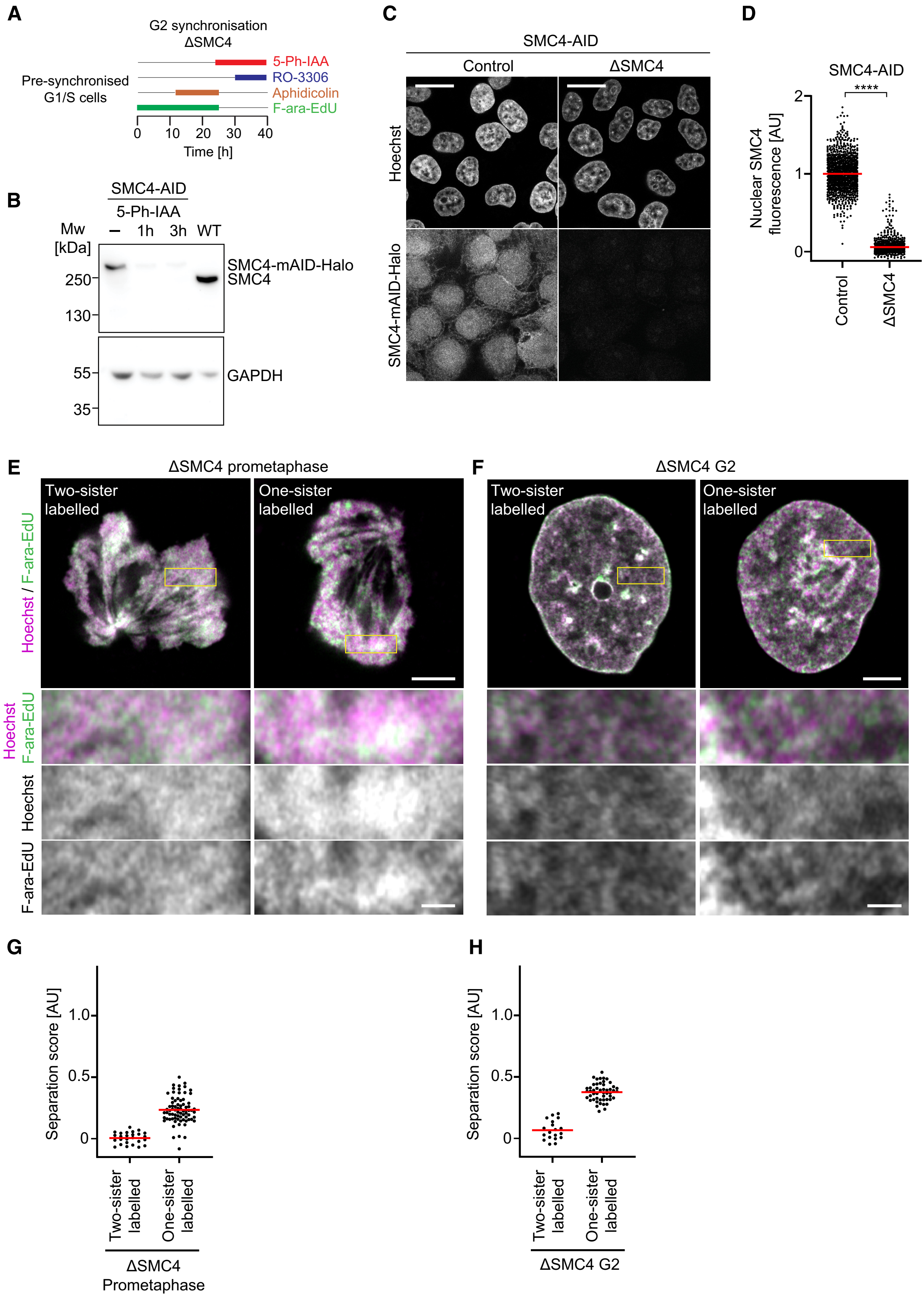

Figure EV3.

© 2023 The Authors

Figure EV4. Validation of protein depletion efficiency and sister labelling controls in NIPBL-AID cells.

A Schematic of experimental procedure for generation of one-sister labelled chromatids in ΔNIPBL G2 cells. Cells were treated with the requisite compounds as indicated; the end point indicates the time of fixation. Prometaphase samples were generated by washing out RO-3306 and releasing into medium containing STLC.

B Immunoblot analysis of NIPBL in wild-type (WT) cells, untreated NIPBL-AID cells and NIPBL-AID cells treated for either 1 or 3 h with 5-Ph-IAA. Representative example of $n = 2$ biological replicates.

C Immunofluorescence analysis of HeLa cells homozygously tagged for mEGFP-mAID-NIPBL and stably expressing $OsTIR1^{F74G}$. Cells were incubated for 2 h with (ΔNIPBL) or without (Control) 1 μM 5-Ph-IAA before subsequently fixing and staining for mEGFP with an anti-GFP nanobody. DNA was stained with Hoechst 33342.

D Quantification of mean nuclear mEGFP-mAID-NIPBL fluorescence per cell, as shown in (C). Dots represent individual cells; red bars indicate the mean. For each condition three experimental replicates were performed. $n = 1{,}205$ cells analysed for control NIPBL-AID cells, $n = 1{,}223$ cells analysed for ΔNIPBL cells. Significance was tested using a two-tailed Mann–Whitney *U* test; $P < 10^{-324}$ (precision limit of floating-point arithmetic).

E Representative images from ΔNIPBL prometaphase cells labelled on one or two sister chromatids as indicated.

F Representative images from ΔNIPBL G2 cells labelled on one or two sister chromatids as indicated.

G Quantification of sister chromatid separation as in Fig 1D and E, for ΔNIPBL prometaphase cells labelled on one or two sister chromatids. Dots represent individual cells; red bars indicate the mean. Two-sister labelled ($n = 24$ cells) and one-sister labelled ($n = 48$ cells) cells were analysed. Quantification for one-sister labelled ΔNIPBL prometaphase cells is the same as in Fig 1D (ΔNIPBL) to allow side-by-side comparison with two-sister labelled cells. A different example prometaphase cell labelled on one sister chromatid is shown.

H Quantification of sister chromatid separation as in Fig 1D and E, for ΔNIPBL G2 cells labelled on one or two sister chromatids. Dots represent individual cells; red bars indicate the mean. Two-sister labelled ($n = 54$ cells) and one-sister labelled ($n = 70$ cells) cells were analysed. Quantification for one-sister labelled ΔNIPBL G2 cells is the same as in Fig 1E (ΔNIPBL) to allow side by side comparison with two-sister labelled cells. A different example G2 cell labelled on one sister chromatid is shown.

Data information: (****) $P < 0.0001$; two-tailed Mann–Whitney *U* test. Biological replicates: B ($n = 2$), C, D ($n = 2$), (E–H) ΔNIPBL two-sister labelled prometaphase ($n = 3$), ΔNIPBL two-sister labelled G2 ($n = 5$), ΔNIPBL one-sister labelled prometaphase and G2 as in Fig 1. Technical replicates: C, D ($n = 3$), (E–H) ΔNIPBL two-sister labelled prometaphase ($n = 5$), ΔNIPBL two-sister labelled G2 ($n = 8$), ΔNIPBL one-sister labelled prometaphase and G2 as in Fig 1. Images with fields of cells (panel C) are single Z-sections. Images with single cells (panels E, F) are single Z-slices from 3D-stacks. Scale bars fields: 20 μm. Scale bars single cells, large panels: 5 μm, insets: 1 μm.

© 2023 The Authors

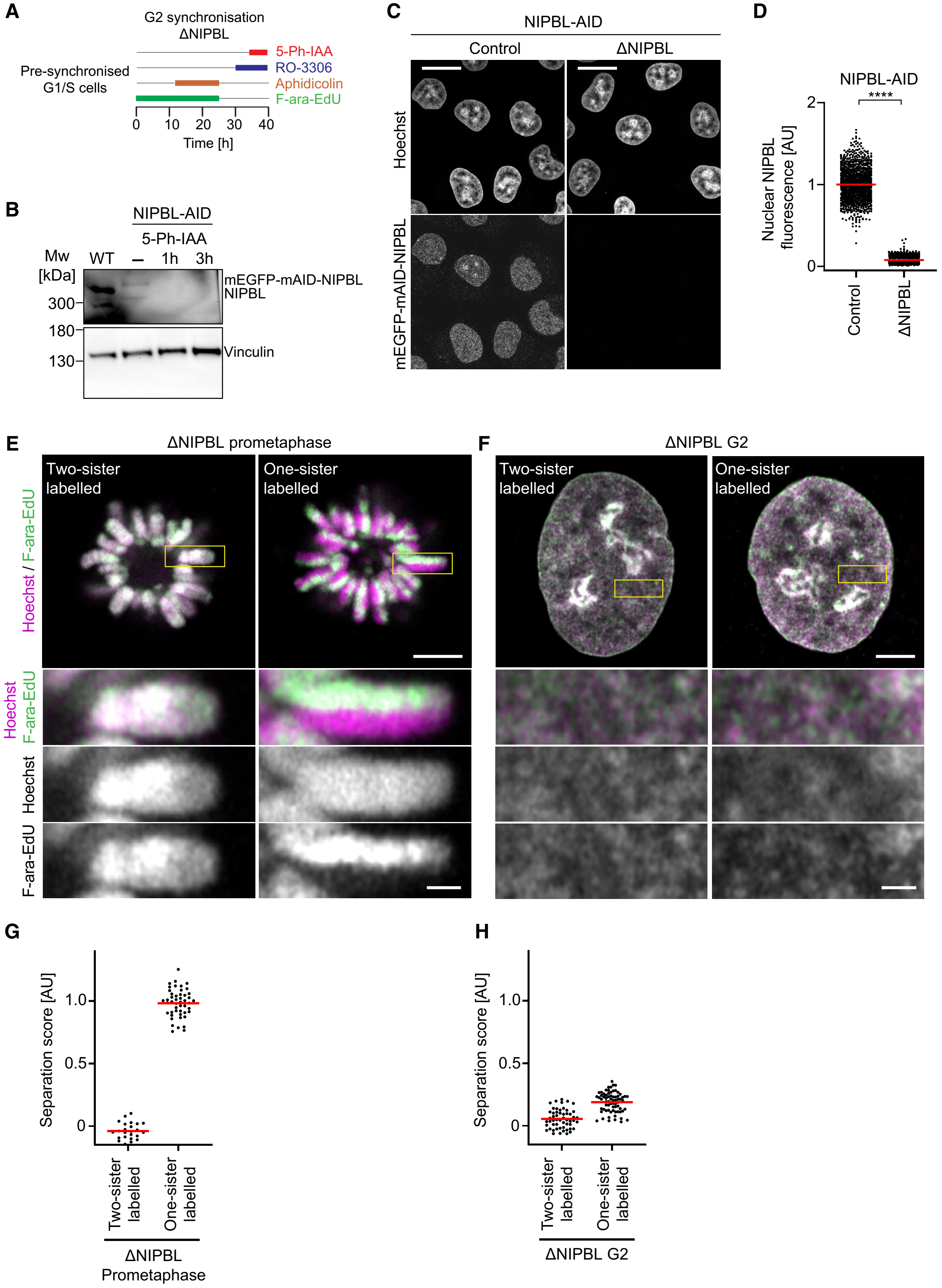

Figure EV4.

 © 2023 The Authors

Figure EV5. Condensin is not required to resolve sister DNAs in WAPL depleted G2 cells.

A Schematic of experimental procedure for generation of one-sister labelled chromatids in ΔSMC4 ΔWAPL G2 cells. Cells were treated with the requisite compounds as indicated; the end point indicates the time of fixation.

B Representative images of one-sister labelled sister chromatids from ΔWAPL or ΔSMC4 ΔWAPL G2 cells, as indicated. Cells were labelled and fixed as in Fig 2B. SMC4 was depleted in G1 through the addition of 5-Ph-IAA 1 h before the final release into S phase such that cells progressed through S and G2 phase in the absence of condensins. WAPL was depleted in G2 phase after completion of DNA replication to avoid potential effects of WAPL depletion on cohesion establishment. SMC4 was visualised by staining with HaloTag TMR Ligand. Wild-type cells were stained with HaloTag TMR Ligand to determine the fluorescence background for background subtraction. The SMC4 channel is displayed after background subtraction.

C Quantification of sister chromatid separation as in Fig 1D and E. ΔWAPL G2 (n = 45 cells) and ΔSMC4 ΔWAPL G2 cells (n = 39 cells) cells were analysed. Dots represent individual cells; red bars indicate the mean.

D Validation of cell cycle stage in ΔWAPL cells by phospho-H3-Ser10 immunofluorescence in cells arrested in prometaphase (prometa) by STLC and cells arrested in G2 by RO-3306.

E Quantification of mean phospho-H3-Ser10 fluorescence for central Z-stack slices for the conditions shown in (D). Dots represent individual cells; red bars indicate the mean. ΔWAPL prometaphase (n = 19 cells) and ΔWAPL G2 (n = 33 cells) cells were analysed. Significance was tested using a two-tailed Mann–Whitney U test; $P = 2.72 \times 10^{-9}$.

F Immunoblot analysis of Sororin in WAPL-dTAG cells treated with either Control (Ctrl) or Sororin (Sor) siRNAs as indicated, as in Fig 2E. Cells were harvested 40 h after transfection. Representative example of n = 2 biological replicates.

Data information: (****) $P < 0.0001$; two-tailed Mann–Whitney U test. Biological replicates: (B, C) ΔWAPL G2 (n = 3), ΔWAPL ΔSMC4 G2 (n = 3). F (n = 2). Technical replicates: (B, C) ΔWAPL G2 (n = 5), ΔWAPL ΔSMC4 G2 (n = 5), D, E (n = 3). All microscopy images are single Z-slices from 3D-stacks. Yellow boxes indicate inset regions. Scale bars large panels: 5 µm, insets: 2 µm.

© 2023 The Authors

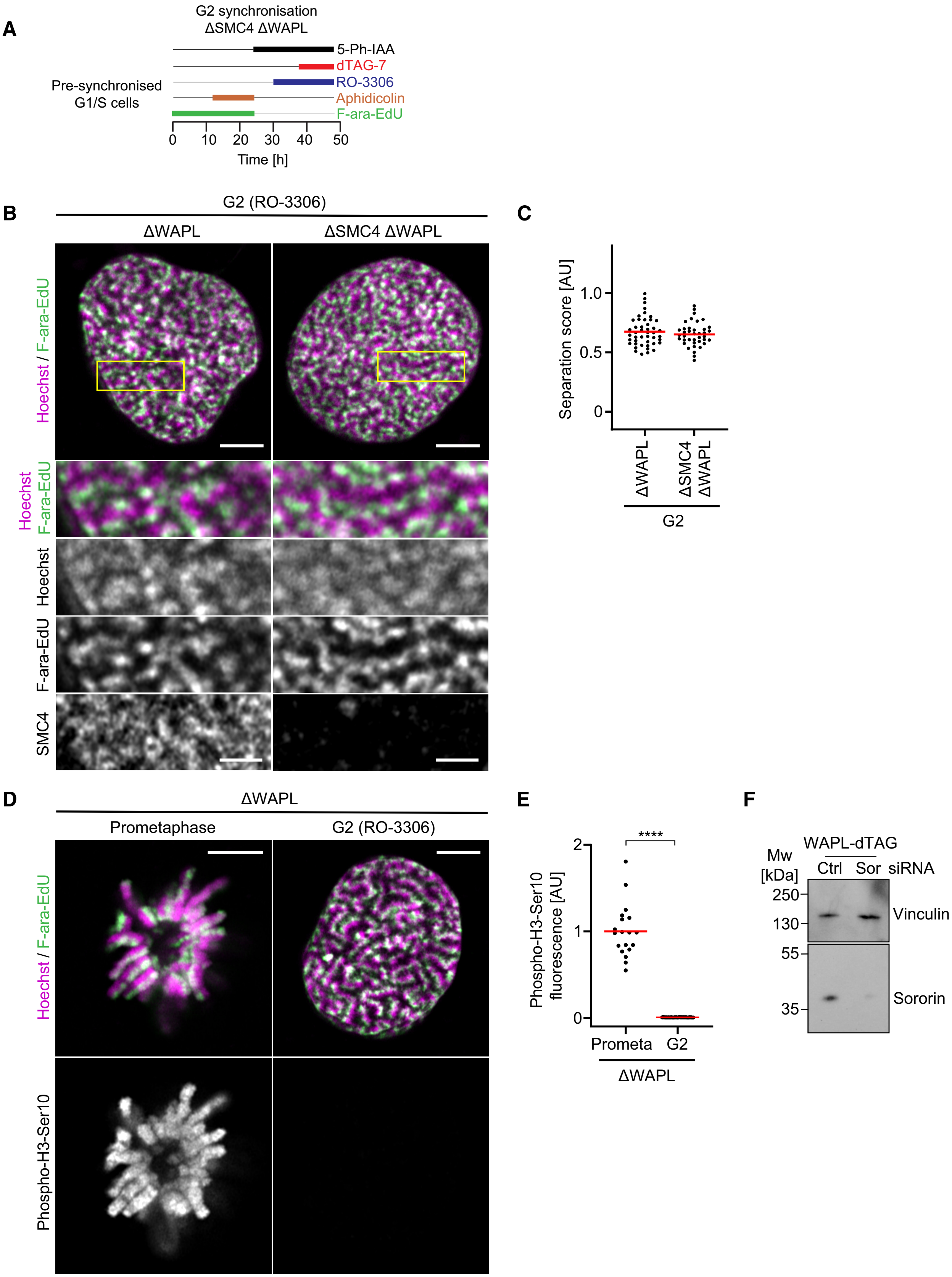

Figure EV5.

 © 2023 The Authors

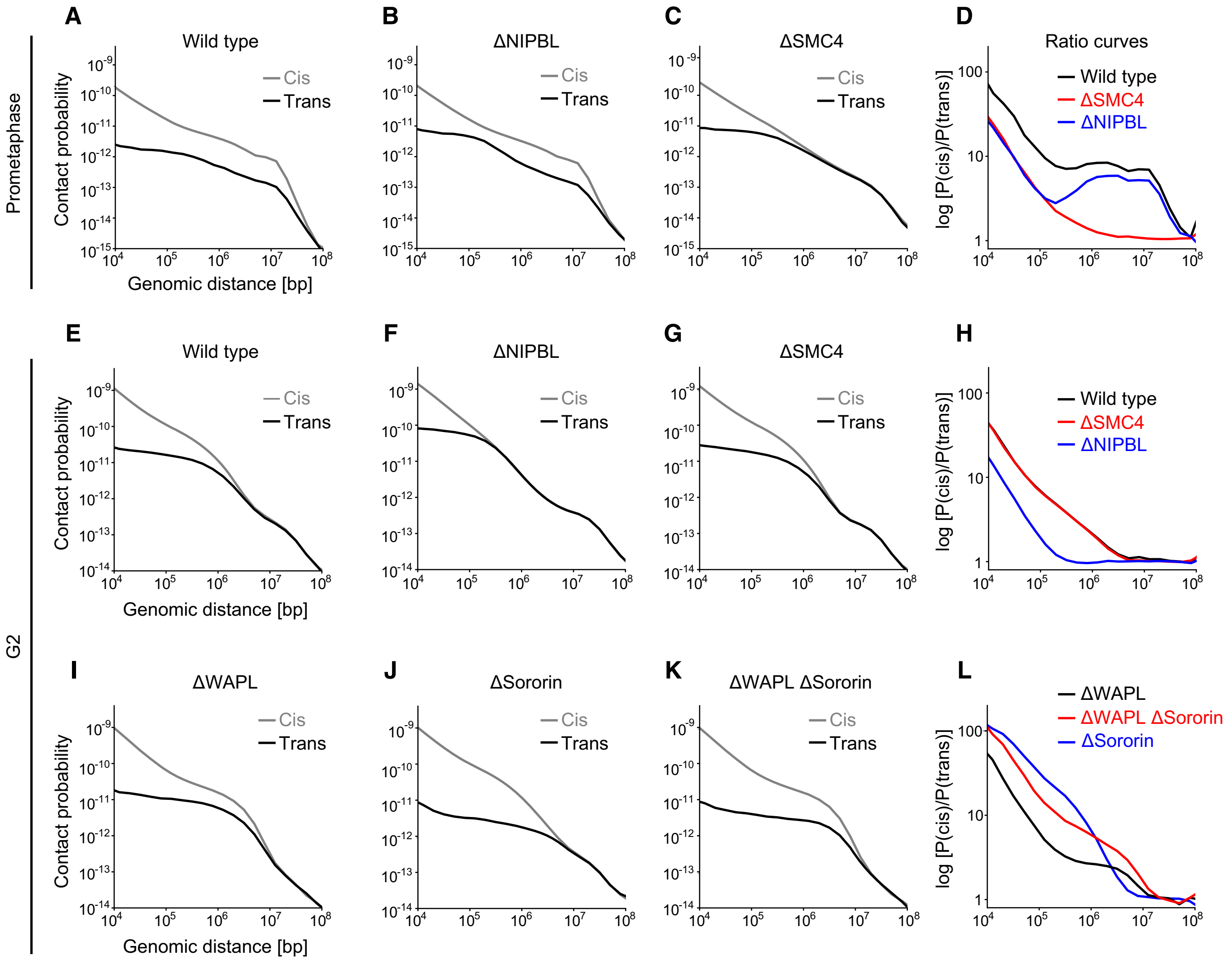

Figure EV6. scsHi-C contact probability curves.

A–L Sister-chromatid-sensitive Hi-C experiments were analysed by calculating the average contact probability for cis sister and trans sister contacts over variable genomic intervals. Merged curves of the individual replicates are shown for all panels. (A) Cis sister and trans sister contact probability curves for wild-type prometaphase cells. (B) Cis sister and trans sister contact probability curves for ΔNIPBL prometaphase cells. (C) Cis sister and trans sister contact probability curves for ΔSMC4 prometaphase cells. (D) Ratio curves plotting cis sister contact probability / trans sister contact probability against genomic distance for the conditions in (A–C). (E) Cis sister and trans sister contact probability curves for wild-type G2 cells. (F) Cis sister and trans sister contact probability curves for ΔNIPBL G2 cells. (G) Cis sister and trans sister contact probability curves for ΔSMC4 G2 cells. (H) Ratio curves plotting cis sister contact probability/trans sister contact probability against genomic distance for the conditions in (E–G). (I) Cis sister and trans sister contact probability curves for ΔWAPL G2 cells. (J) Cis sister and trans sister contact probability curves for ΔSororin G2 cells. (K) Cis sister and trans sister contact probability curves for ΔWAPL ΔSororin G2 cells. (L) Ratio curves plotting cis sister contact probability / trans contact probability against genomic distance for the conditions in (I–K). Biological replicates: Wild-type prometaphase (n = 2), ΔNIPBL prometaphase (n = 3), ΔSMC4 prometaphase (n = 2), wild-type G2 (n = 11), ΔNIPBL G2 (n = 10), ΔSMC4 G2 (n = 4), ΔWAPL G2 (n = 6), ΔSororin G2 (n = 3), ΔWAPL ΔSororin G2 (n = 4).

© 2023 The Authors
