# Supplementary figures and images for: Cohesin‐mediated DNA loop extrusion resolves sister chromatids in G2 phase (part 1 of 3)
Source: EMBO J. 2023 Jun 26;42(16):e113475. doi: 10.15252/embj.2023113475 (PMC10425840; doi:10.15252/embj.2023113475)

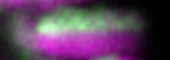

Supplement: Supplementary file 4 — Source Data for Figure 1 [file EMBJ-42-e113475-s007.zip › sd_figure1/panel_b/one_sister_prometa/insets/RGB_220207_5389_WT_c2_rep2_prometa_60min_stlc_hemi_zoom5-03-77.czi #3.tif_registered_670_x_670_slice62_8bit_inset_170-60_hoechst_edu.tif.tif]

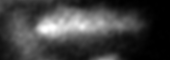

Supplement: Supplementary file 4 — Source Data for Figure 1 [file EMBJ-42-e113475-s007.zip › sd_figure1/panel_b/one_sister_prometa/insets/RGB_220207_5389_WT_c2_rep2_prometa_60min_stlc_hemi_zoom5-03-77.czi #3.tif_registered_670_x_670_slice62_inset_170-60_edu_gray.tif]

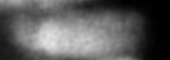

Supplement: Supplementary file 4 — Source Data for Figure 1 [file EMBJ-42-e113475-s007.zip › sd_figure1/panel_b/one_sister_prometa/insets/RGB_220207_5389_WT_c2_rep2_prometa_60min_stlc_hemi_zoom5-03-77.czi #3.tif_registered_670_x_670_slice62_inset_170-60_hoechst_gray.tif]

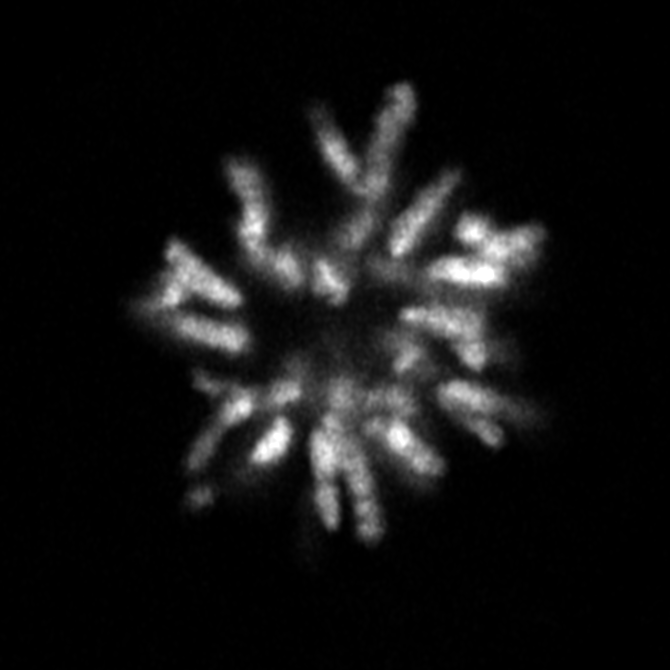

Supplement: Supplementary file 4 — Source Data for Figure 1 [file EMBJ-42-e113475-s007.zip › sd_figure1/panel_b/one_sister_prometa/whole_cell/16bit_220207_5389_WT_c2_rep2_prometa_60min_stlc_hemi_zoom5-03-77.czi #3.tif_registered_670_x_670_slice62_hoechst_edu_cropped.tif]

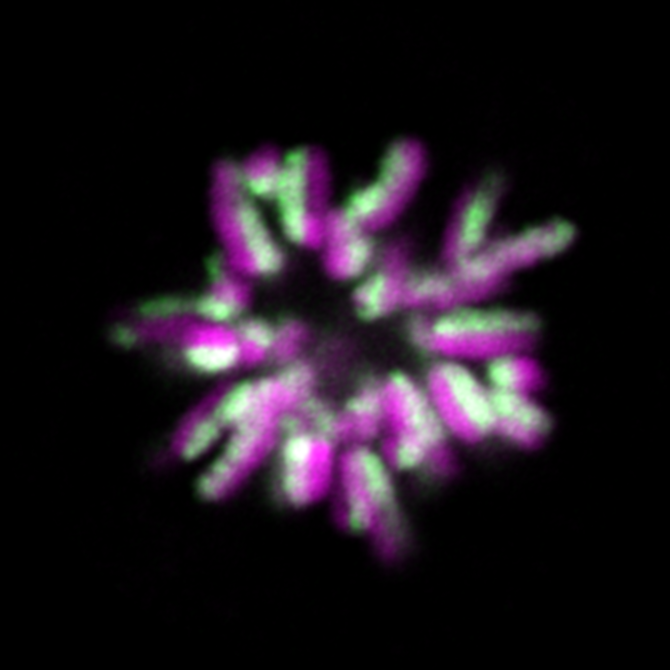

Supplement: Supplementary file 4 — Source Data for Figure 1 [file EMBJ-42-e113475-s007.zip › sd_figure1/panel_b/one_sister_prometa/whole_cell/RGB_220207_5389_WT_c2_rep2_prometa_60min_stlc_hemi_zoom5-03-77.czi #3.tif_registered_670_x_670_slice62_rotated_cropped_hoechst_edu.tif]

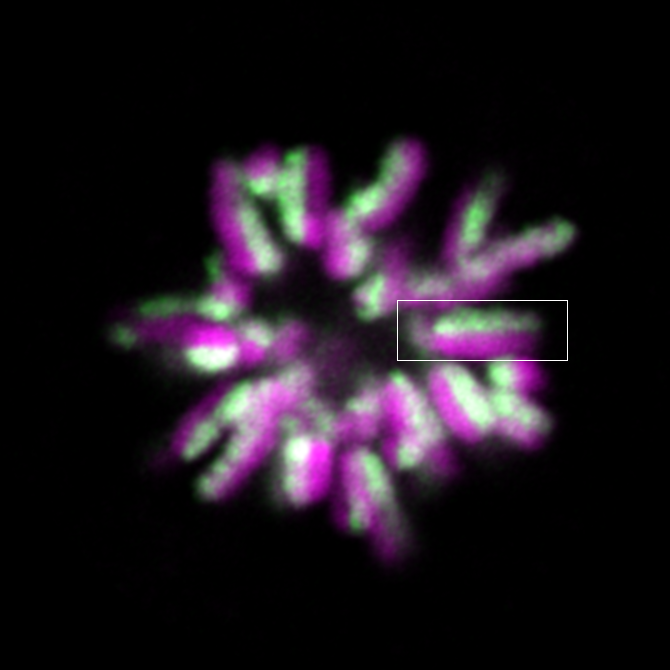

Supplement: Supplementary file 4 — Source Data for Figure 1 [file EMBJ-42-e113475-s007.zip › sd_figure1/panel_b/one_sister_prometa/whole_cell/RGB_220207_5389_WT_c2_rep2_prometa_60min_stlc_hemi_zoom5-03-77.czi #3.tif_registered_670_x_670_slice62_rotated_cropped_hoechst_edu_draw_roi.tif]

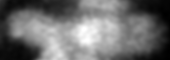

Supplement: Supplementary file 4 — Source Data for Figure 1 [file EMBJ-42-e113475-s007.zip › sd_figure1/panel_b/two_sister_prometa/insets/16bit_220207_5389_WT_c2_rep2_prometa_60min_stlc_fully_zoom5-03-25.czi #8.tif_registered_slice50_8bit_rotated_edu_gray.tif]

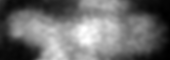

Supplement: Supplementary file 4 — Source Data for Figure 1 [file EMBJ-42-e113475-s007.zip › sd_figure1/panel_b/two_sister_prometa/insets/16bit_220207_5389_WT_c2_rep2_prometa_60min_stlc_fully_zoom5-03-25.czi #8.tif_registered_slice50_8bit_rotated_hoechst_edu_inset.tif]

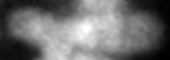

Supplement: Supplementary file 4 — Source Data for Figure 1 [file EMBJ-42-e113475-s007.zip › sd_figure1/panel_b/two_sister_prometa/insets/16bit_220207_5389_WT_c2_rep2_prometa_60min_stlc_fully_zoom5-03-25.czi #8.tif_registered_slice50_8bit_rotated_hoechst_gray.tif]

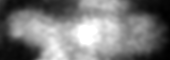

Supplement: Supplementary file 4 — Source Data for Figure 1 [file EMBJ-42-e113475-s007.zip › sd_figure1/panel_b/two_sister_prometa/insets/RGB_220207_5389_WT_c2_rep2_prometa_60min_stlc_fully_zoom5-03-25.czi #8.tif_registered_slice50_8bit_rotated_edu_gray.tif]

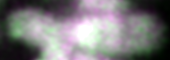

Supplement: Supplementary file 4 — Source Data for Figure 1 [file EMBJ-42-e113475-s007.zip › sd_figure1/panel_b/two_sister_prometa/insets/RGB_220207_5389_WT_c2_rep2_prometa_60min_stlc_fully_zoom5-03-25.czi #8.tif_registered_slice50_8bit_rotated_hoechst_edu_inset.tif hoechst_edu.tif]

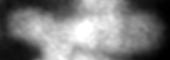

Supplement: Supplementary file 4 — Source Data for Figure 1 [file EMBJ-42-e113475-s007.zip › sd_figure1/panel_b/two_sister_prometa/insets/RGB_220207_5389_WT_c2_rep2_prometa_60min_stlc_fully_zoom5-03-25.czi #8.tif_registered_slice50_8bit_rotated_hoechst_gray.tif]

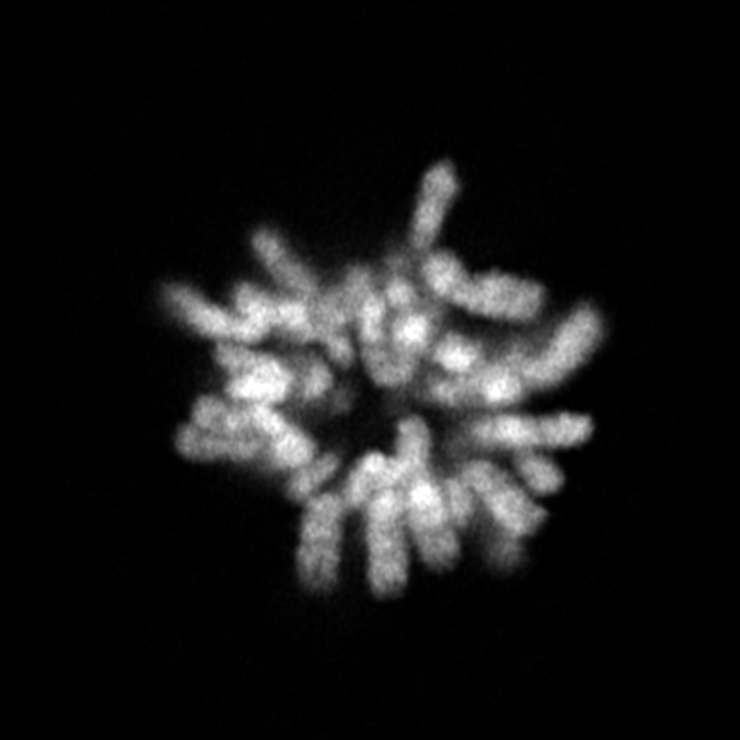

Supplement: Supplementary file 4 — Source Data for Figure 1 [file EMBJ-42-e113475-s007.zip › sd_figure1/panel_b/two_sister_prometa/whole_cell/16bit_220207_5389_WT_c2_rep2_prometa_60min_stlc_fully_zoom5-03-25.czi #8.tif_registered_slice50.tif]

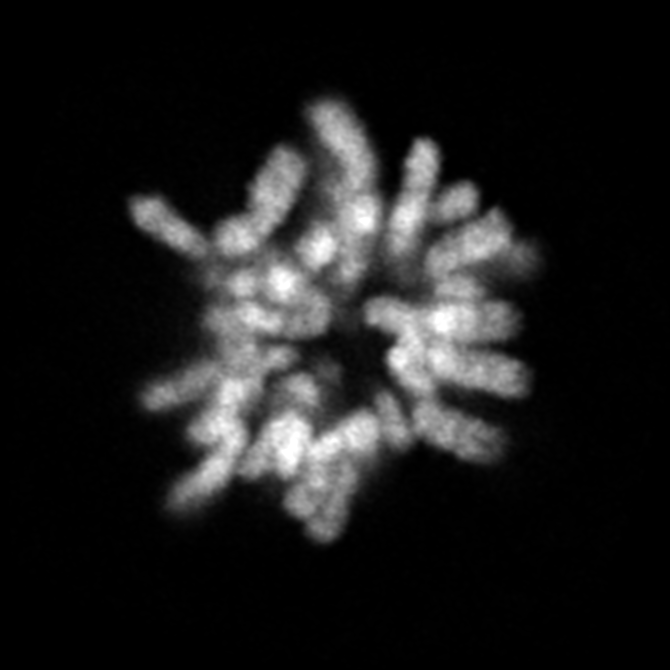

Supplement: Supplementary file 4 — Source Data for Figure 1 [file EMBJ-42-e113475-s007.zip › sd_figure1/panel_b/two_sister_prometa/whole_cell/16bit_220207_5389_WT_c2_rep2_prometa_60min_stlc_fully_zoom5-03-25.czi #8.tif_registered_slice50_8bit_rotated_cropped_hoechst_edu.tif]

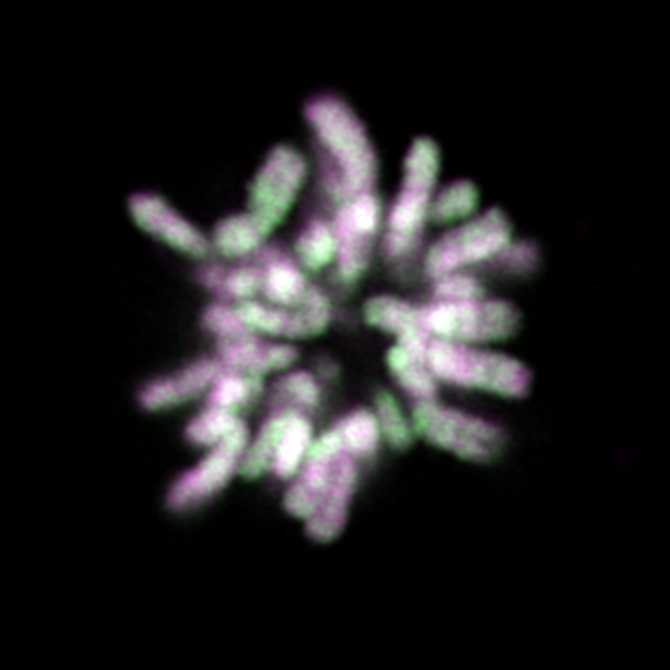

Supplement: Supplementary file 4 — Source Data for Figure 1 [file EMBJ-42-e113475-s007.zip › sd_figure1/panel_b/two_sister_prometa/whole_cell/RGB_220207_5389_WT_c2_rep2_prometa_60min_stlc_fully_zoom5-03-25.czi #8.tif_registered_slice50_8bit_rotated_hoechst_edu.tif (RGB).tif]

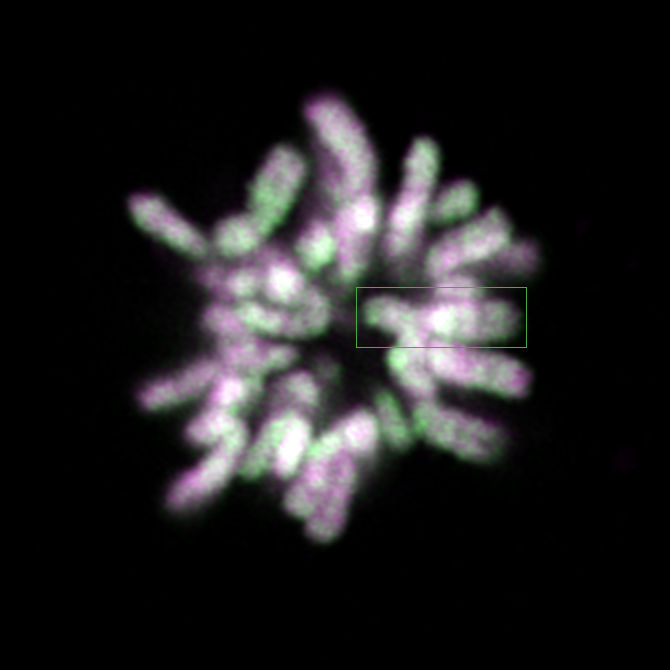

Supplement: Supplementary file 4 — Source Data for Figure 1 [file EMBJ-42-e113475-s007.zip › sd_figure1/panel_b/two_sister_prometa/whole_cell/RGB_220207_5389_WT_c2_rep2_prometa_60min_stlc_fully_zoom5-03-25.czi #8.tif_registered_slice50_8bit_rotated_inset_hoechst_edu_marked.tif (RGB)_draw_roi.tif]

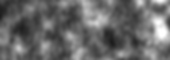

Supplement: Supplementary file 4 — Source Data for Figure 1 [file EMBJ-42-e113475-s007.zip › sd_figure1/panel_c/one_sister_g2/insets/8bit_211110_5325_WT_15h_release_ctrl_hemi_zoom4_8_stain_scc1_cycb1_g2_rep1_czi-07-10.tif_registered-1_slice30_inset_edu_170-60.tif]

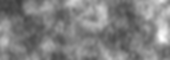

Supplement: Supplementary file 4 — Source Data for Figure 1 [file EMBJ-42-e113475-s007.zip › sd_figure1/panel_c/one_sister_g2/insets/8bit_211110_5325_WT_15h_release_ctrl_hemi_zoom4_8_stain_scc1_cycb1_g2_rep1_czi-07-10.tif_registered-1_slice30_inset_hoechst_170-60.tif]

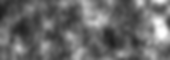

Supplement: Supplementary file 4 — Source Data for Figure 1 [file EMBJ-42-e113475-s007.zip › sd_figure1/panel_c/one_sister_g2/insets/RGB_211110_5325_WT_15h_release_ctrl_hemi_zoom4_8_stain_scc1_cycb1_g2_rep1_czi-07-10.tif_registered-1_slice30_inset_edu_170-60.tif]

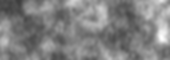

Supplement: Supplementary file 4 — Source Data for Figure 1 [file EMBJ-42-e113475-s007.zip › sd_figure1/panel_c/one_sister_g2/insets/RGB_211110_5325_WT_15h_release_ctrl_hemi_zoom4_8_stain_scc1_cycb1_g2_rep1_czi-07-10.tif_registered-1_slice30_inset_hoechst_170-60.tif]

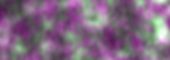

Supplement: Supplementary file 4 — Source Data for Figure 1 [file EMBJ-42-e113475-s007.zip › sd_figure1/panel_c/one_sister_g2/insets/RGB_211110_5325_WT_15h_release_ctrl_hemi_zoom4_8_stain_scc1_cycb1_g2_rep1_czi-07-10.tif_registered-1_slice30_inset_hoechst_edu_170-60.tif]

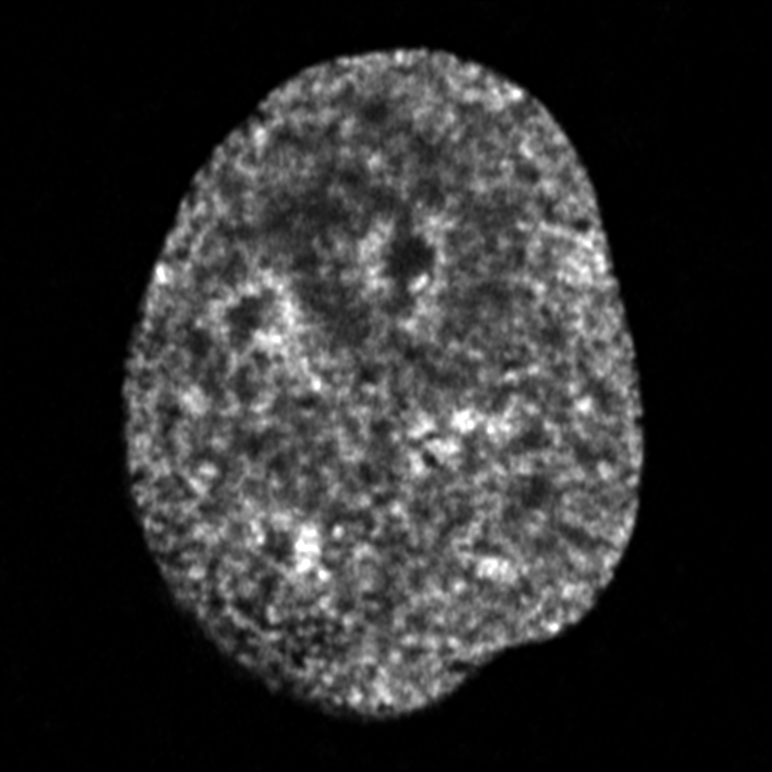

Supplement: Supplementary file 4 — Source Data for Figure 1 [file EMBJ-42-e113475-s007.zip › sd_figure1/panel_c/one_sister_g2/whole_cell/16bit_211110_5325_WT_15h_release_ctrl_hemi_zoom4_8_stain_scc1_cycb1_g2_rep1_czi-07-10.tif_registered-1_slice30_hoechst_edu.tif]

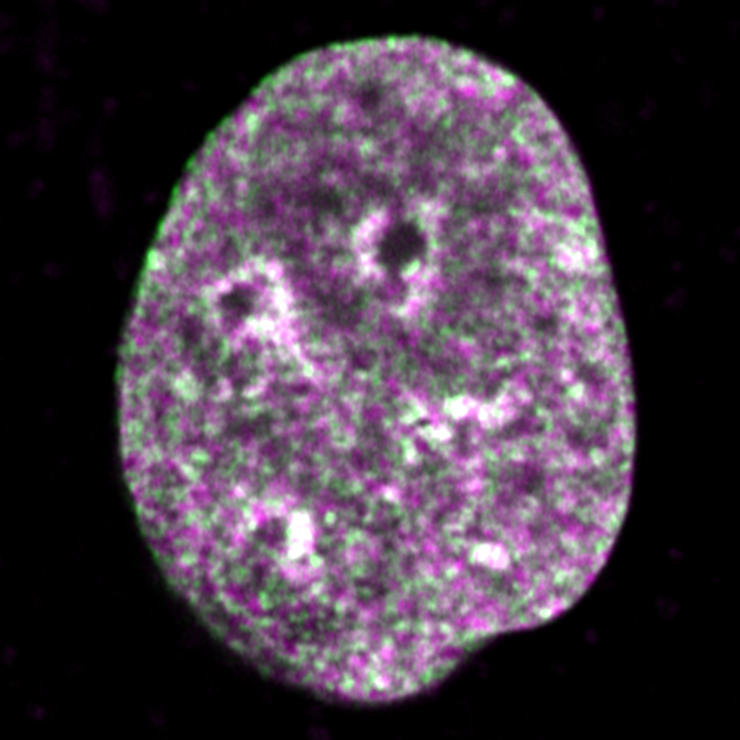

Supplement: Supplementary file 4 — Source Data for Figure 1 [file EMBJ-42-e113475-s007.zip › sd_figure1/panel_c/one_sister_g2/whole_cell/RGB_211110_5325_WT_15h_release_ctrl_hemi_zoom4_8_stain_scc1_cycb1_g2_rep1_czi-07-10.tif_registered_slice30_cropped_hoechst_edu.tif.tif]

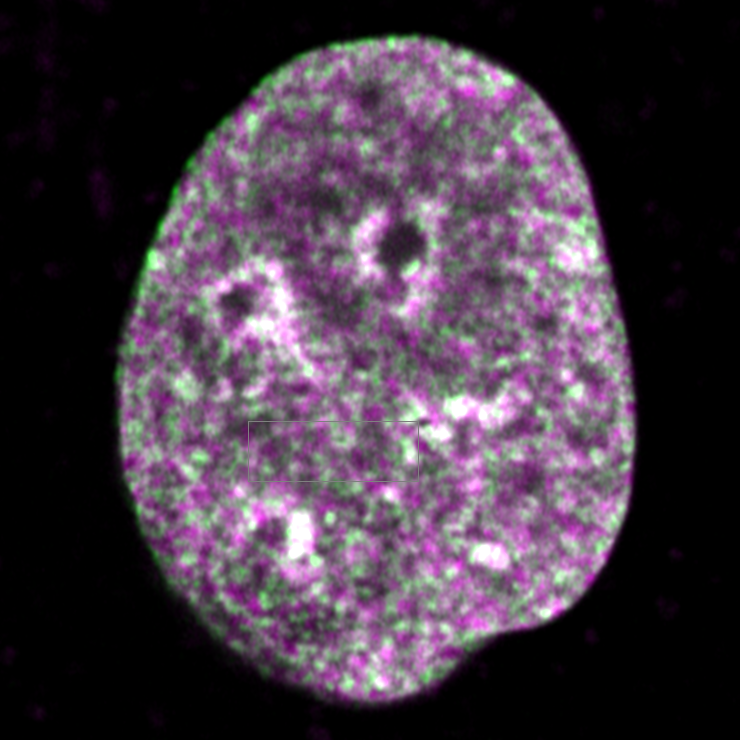

Supplement: Supplementary file 4 — Source Data for Figure 1 [file EMBJ-42-e113475-s007.zip › sd_figure1/panel_c/one_sister_g2/whole_cell/RGB_211110_5325_WT_15h_release_ctrl_hemi_zoom4_8_stain_scc1_cycb1_g2_rep1_czi-07-10.tif_registered-1_slice30_cropped_hoechst_edu_draw_roi.tif]

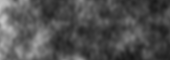

Supplement: Supplementary file 4 — Source Data for Figure 1 [file EMBJ-42-e113475-s007.zip › sd_figure1/panel_c/two_sister_g2/insets/8bit_220601_5517_wt_fully_15h_release_g2_rep1_zoom4_8-03-56.czi #1.tif_registered_slice34_170x60_edu_gray.tif]

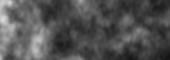

Supplement: Supplementary file 4 — Source Data for Figure 1 [file EMBJ-42-e113475-s007.zip › sd_figure1/panel_c/two_sister_g2/insets/8bit_220601_5517_wt_fully_15h_release_g2_rep1_zoom4_8-03-56.czi #1.tif_registered_slice34_170x60_hoechst_gray.tif]

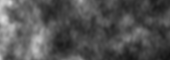

Supplement: Supplementary file 4 — Source Data for Figure 1 [file EMBJ-42-e113475-s007.zip › sd_figure1/panel_c/two_sister_g2/insets/RGB_20601_5517_wt_fully_15h_release_g2_rep1_zoom4_8-03-56.czi #1.tif_registered_slice34_170x60_hoechst_gray_rgb.tif]

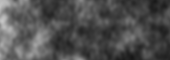

Supplement: Supplementary file 4 — Source Data for Figure 1 [file EMBJ-42-e113475-s007.zip › sd_figure1/panel_c/two_sister_g2/insets/RGB_220601_5517_wt_fully_15h_release_g2_rep1_zoom4_8-03-56.czi #1.tif_registered_slice34_170x60_edu_gray_rgb.tif]

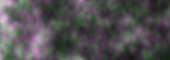

Supplement: Supplementary file 4 — Source Data for Figure 1 [file EMBJ-42-e113475-s007.zip › sd_figure1/panel_c/two_sister_g2/insets/RGB_220601_5517_wt_fully_15h_release_g2_rep1_zoom4_8-03-56.czi #1.tif_registered_slice34_hoechst_edu.tif (RGB)_170x60.tif (RGB).tif]

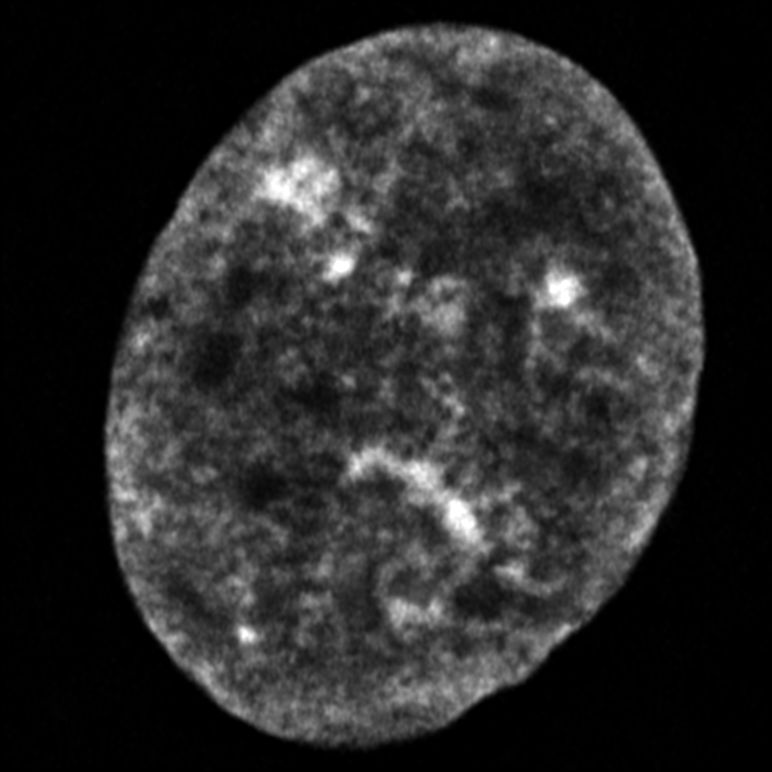

Supplement: Supplementary file 4 — Source Data for Figure 1 [file EMBJ-42-e113475-s007.zip › sd_figure1/panel_c/two_sister_g2/whole_cell/16bit_220601_5517_wt_fully_15h_release_g2_rep1_zoom4_8-03-56.czi #1.tif_registered_slice34_hoechst_edu.tif]

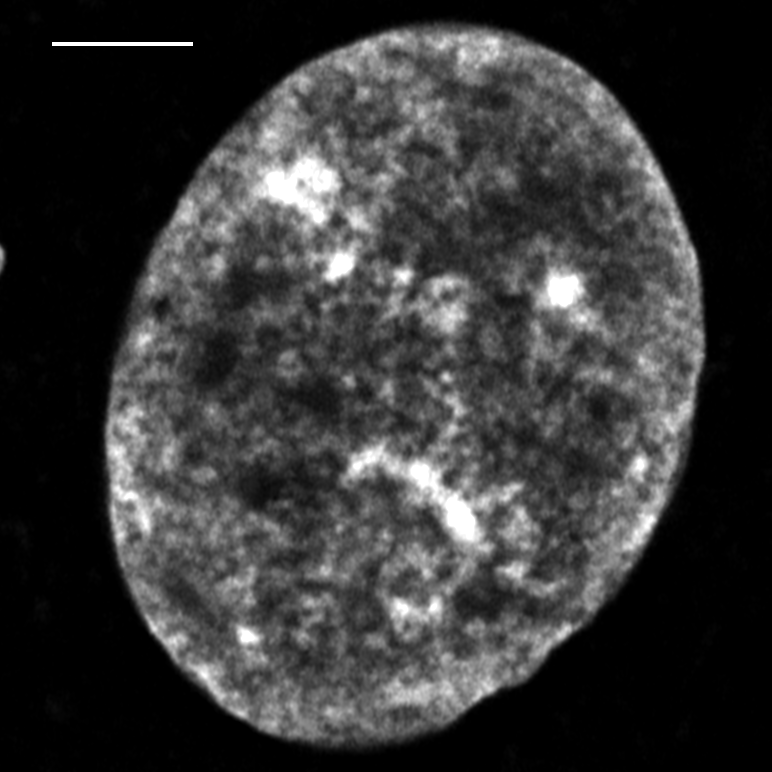

Supplement: Supplementary file 4 — Source Data for Figure 1 [file EMBJ-42-e113475-s007.zip › sd_figure1/panel_c/two_sister_g2/whole_cell/8bit_220601_5517_wt_fully_15h_release_g2_rep1_zoom4_8-03-56.czi #1.tif_registered_slice34_8bit_hoechst_scalebar_gray.tif]

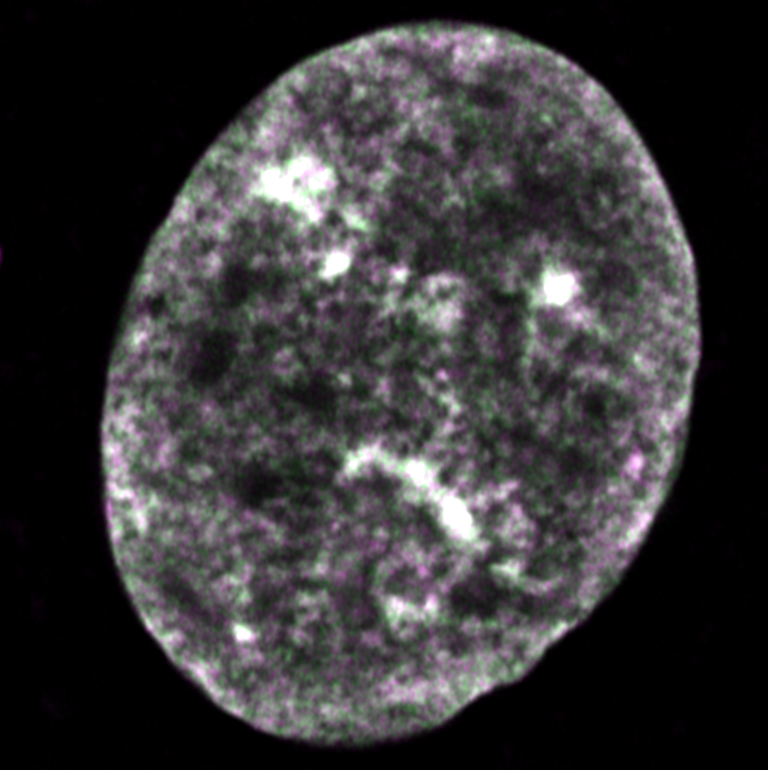

Supplement: Supplementary file 4 — Source Data for Figure 1 [file EMBJ-42-e113475-s007.zip › sd_figure1/panel_c/two_sister_g2/whole_cell/RGB_220601_5517_wt_fully_15h_release_g2_rep1_zoom4_8-03-56.czi #1.tif_registered_slice34_8bit_hoechst_edu.tif (RGB).tif]

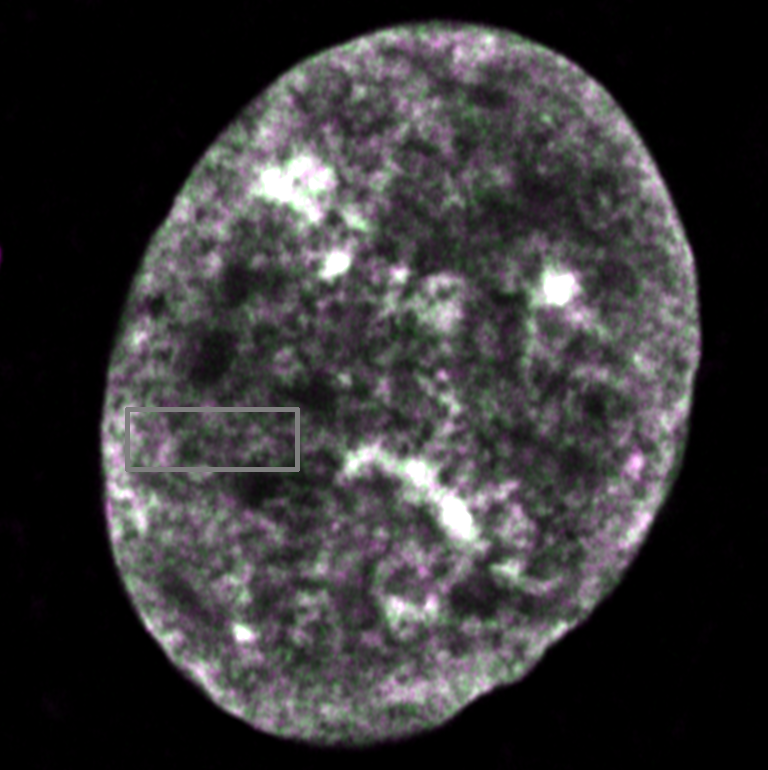

Supplement: Supplementary file 4 — Source Data for Figure 1 [file EMBJ-42-e113475-s007.zip › sd_figure1/panel_c/two_sister_g2/whole_cell/RGB_220601_5517_wt_fully_15h_release_g2_rep1_zoom4_8-03-56.czi #1.tif_registered_slice34_8bit_hoechst_edu.tif_draw_roi.tif]

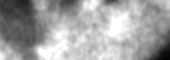

Supplement: Supplementary file 4 — Source Data for Figure 1 [file EMBJ-42-e113475-s007.zip › sd_figure1/panel_f/insets/8bit_5275_2056_smc4_depleted_hemi_prometa_30min_stlc_zoom5_rep1-33-115.czi #3.tif_registered-1_slice34_670_670_cropped_170-60_inset_hoechst_gray.tif]

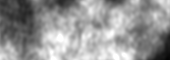

Supplement: Supplementary file 4 — Source Data for Figure 1 [file EMBJ-42-e113475-s007.zip › sd_figure1/panel_f/insets/8bit_5275_2056_smc4_depleted_hemi_prometa_30min_stlc_zoom5_rep1-33-115.czi #3.tif_registered-1_slice34_8bit_670_670_cropped_170-60_inset_edu_gray.tif]

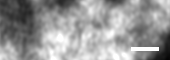

Supplement: Supplementary file 4 — Source Data for Figure 1 [file EMBJ-42-e113475-s007.zip › sd_figure1/panel_f/insets/8bit_5275_2056_smc4_depleted_hemi_prometa_30min_stlc_zoom5_rep1-33-115.czi #3.tif_registered-1_slice34_8bit_670_670_cropped_170-60_inset_edu_gray_sb.tif]

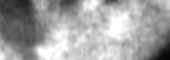

Supplement: Supplementary file 4 — Source Data for Figure 1 [file EMBJ-42-e113475-s007.zip › sd_figure1/panel_f/insets/RGB_5275_2056_smc4_depleted_hemi_prometa_30min_stlc_zoom5_rep1-33-115.czi #3.tif_registered-1_slice34_670_670_cropped_170-60_inset_hoechst_gray.tif]

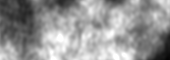

Supplement: Supplementary file 4 — Source Data for Figure 1 [file EMBJ-42-e113475-s007.zip › sd_figure1/panel_f/insets/RGB_5275_2056_smc4_depleted_hemi_prometa_30min_stlc_zoom5_rep1-33-115.czi #3.tif_registered-1_slice34_8bit_670_670_cropped_170-60_inset_edu_gray_RGB.tif]

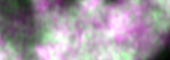

Supplement: Supplementary file 4 — Source Data for Figure 1 [file EMBJ-42-e113475-s007.zip › sd_figure1/panel_f/insets/RGB_5275_2056_smc4_depleted_hemi_prometa_30min_stlc_zoom5_rep1-33-115.czi #3.tif_registered-1_slice34_8bit_670_670_cropped_170-60_inset_hoechst_edu.tif.tif]

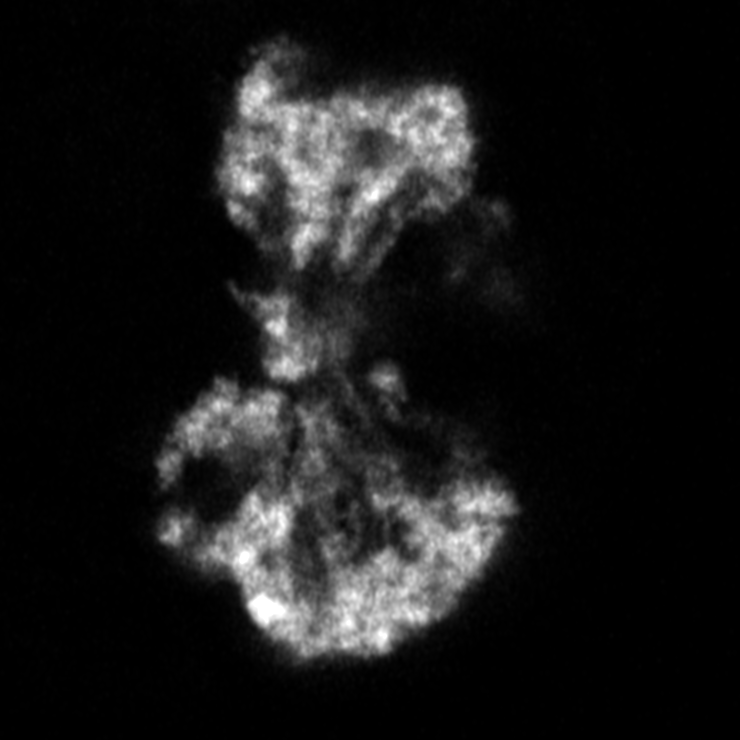

Supplement: Supplementary file 4 — Source Data for Figure 1 [file EMBJ-42-e113475-s007.zip › sd_figure1/panel_f/whole_cell/16bit_5275_2056_smc4_depleted_hemi_prometa_30min_stlc_zoom5_rep1-33-115.czi #3.tif_registered-1_slice34_hoechst_edu.tif]

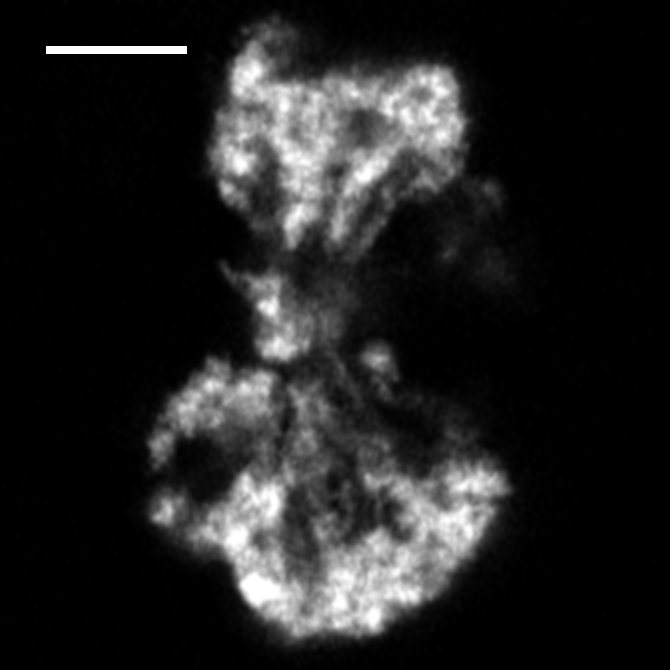

Supplement: Supplementary file 4 — Source Data for Figure 1 [file EMBJ-42-e113475-s007.zip › sd_figure1/panel_f/whole_cell/8bit_5275_2056_smc4_depleted_hemi_prometa_30min_stlc_zoom5_rep1-33-115.czi #3.tif_registered-1_slice34_8bit_670_670_cropped_edu_Gray_sb.tif]

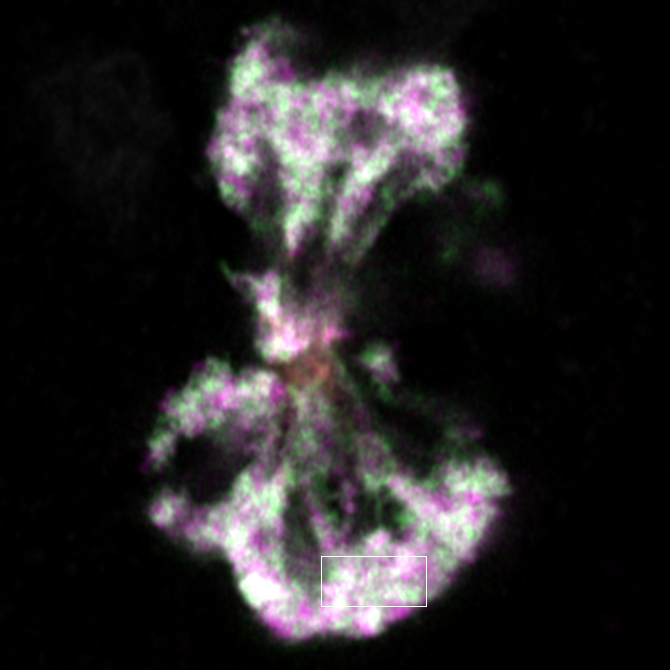

Supplement: Supplementary file 4 — Source Data for Figure 1 [file EMBJ-42-e113475-s007.zip › sd_figure1/panel_f/whole_cell/RGB_5275_2056_smc4_depleted_hemi_prometa_30min_stlc_zoom5_rep1-33-115.czi #3.tif_registered-1_slice34_8bit_670_670_cropped.tif_draw_roi.tif]

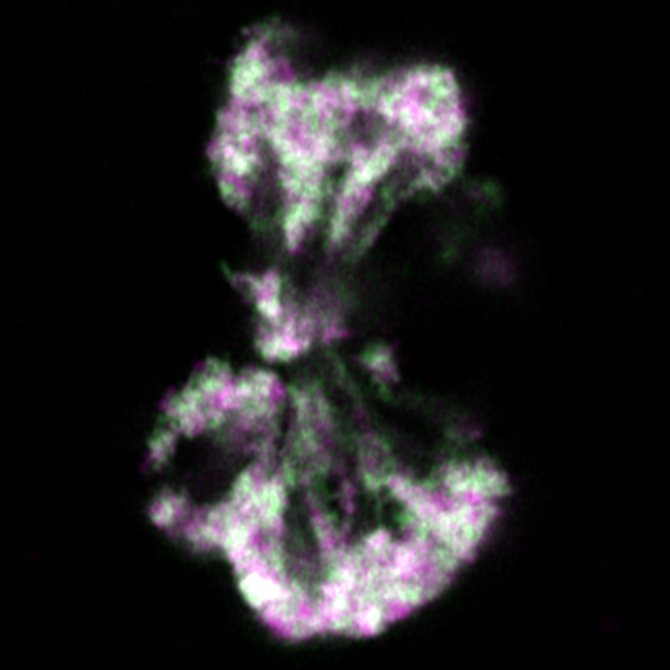

Supplement: Supplementary file 4 — Source Data for Figure 1 [file EMBJ-42-e113475-s007.zip › sd_figure1/panel_f/whole_cell/RGB_5275_2056_smc4_depleted_hemi_prometa_30min_stlc_zoom5_rep1-33-115.czi #3.tif_registered-1_slice34_RGB_670_670_cropped_hoechst_edu.tif (RGB).tif]

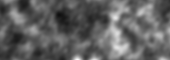

Supplement: Supplementary file 4 — Source Data for Figure 1 [file EMBJ-42-e113475-s007.zip › sd_figure1/panel_g/insets/8bit_220202_5388_2056_smc4_dep_c1_rep1_hemi_zoom4_8-03-101.tif_registered_slice29_8bit_rotated_all_740x740_edu_inset_170-60_gray.tif]

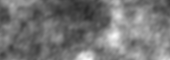

Supplement: Supplementary file 4 — Source Data for Figure 1 [file EMBJ-42-e113475-s007.zip › sd_figure1/panel_g/insets/8bit_220202_5388_2056_smc4_dep_c1_rep1_hemi_zoom4_8-03-101.tif_registered_slice29_8bit_rotated_all_740x740_hoechst_inset_170-60_gray.tif]

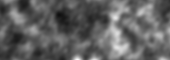

Supplement: Supplementary file 4 — Source Data for Figure 1 [file EMBJ-42-e113475-s007.zip › sd_figure1/panel_g/insets/RGB_220202_5388_2056_smc4_dep_c1_rep1_hemi_zoom4_8-03-101.tif_registered_slice29_8bit_rotated_all_740x740_edu_inset_170-60_gray.tif]

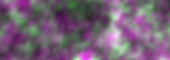

Supplement: Supplementary file 4 — Source Data for Figure 1 [file EMBJ-42-e113475-s007.zip › sd_figure1/panel_g/insets/RGB_220202_5388_2056_smc4_dep_c1_rep1_hemi_zoom4_8-03-101.tif_registered_slice29_8bit_rotated_all_740x740_hoechst_edu_inset_170-60.tif.tif]

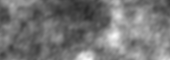

Supplement: Supplementary file 4 — Source Data for Figure 1 [file EMBJ-42-e113475-s007.zip › sd_figure1/panel_g/insets/RGB_220202_5388_2056_smc4_dep_c1_rep1_hemi_zoom4_8-03-101.tif_registered_slice29_8bit_rotated_all_740x740_hoechst_inset_170-60_gray.tif]

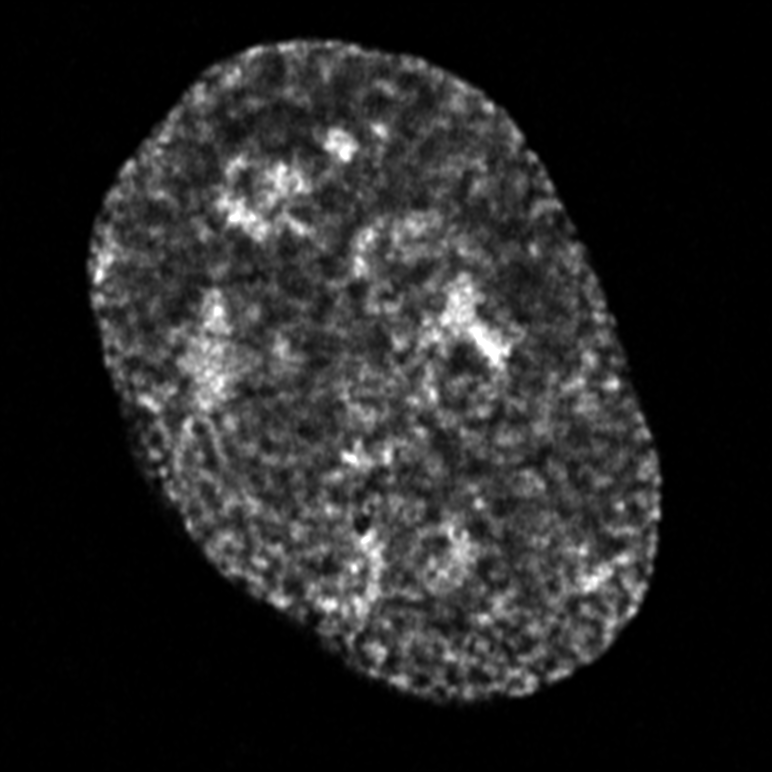

Supplement: Supplementary file 4 — Source Data for Figure 1 [file EMBJ-42-e113475-s007.zip › sd_figure1/panel_g/whole_cell/16bit_220202_5388_2056_smc4_dep_c1_rep1_hemi_zoom4_8-03-101.tif_registered_slice29_hoechst_edu.tif]

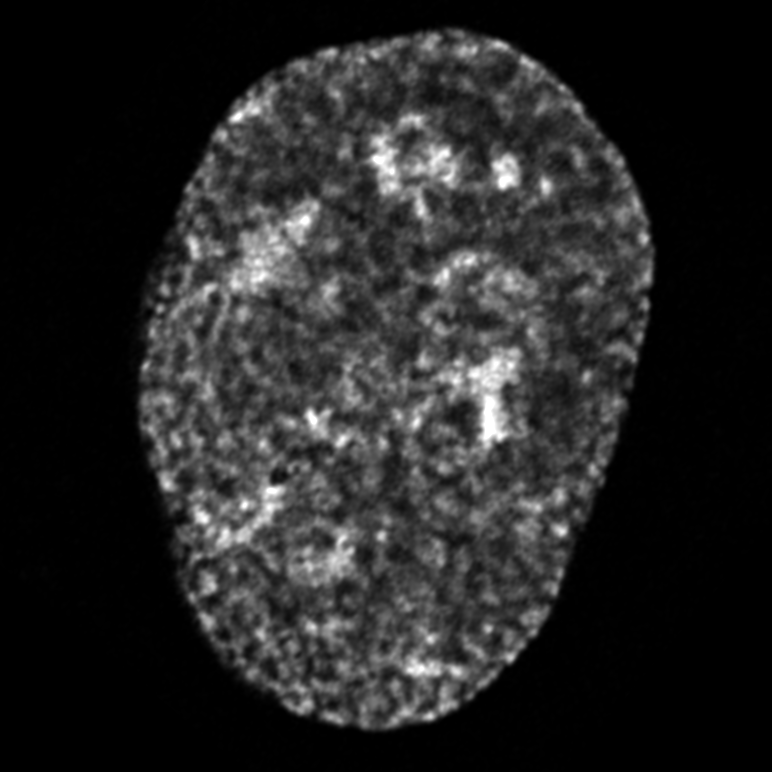

Supplement: Supplementary file 4 — Source Data for Figure 1 [file EMBJ-42-e113475-s007.zip › sd_figure1/panel_g/whole_cell/16bit_220202_5388_2056_smc4_dep_c1_rep1_hemi_zoom4_8-03-101.tif_registered_slice29_rotated_hoechst_edu.tif]

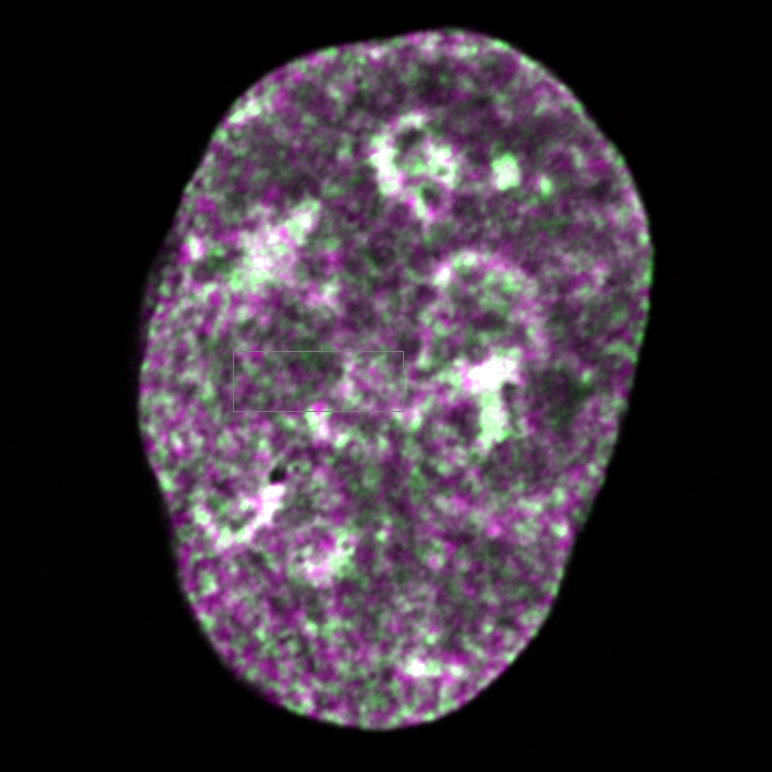

Supplement: Supplementary file 4 — Source Data for Figure 1 [file EMBJ-42-e113475-s007.zip › sd_figure1/panel_g/whole_cell/RGB_220202_5388_2056_smc4_dep_c1_rep1_hemi_zoom4_8-03-101.tif_registered_slice29_8bit_rotated_all_740x740_hoechst_edu_draw_roi.tif]

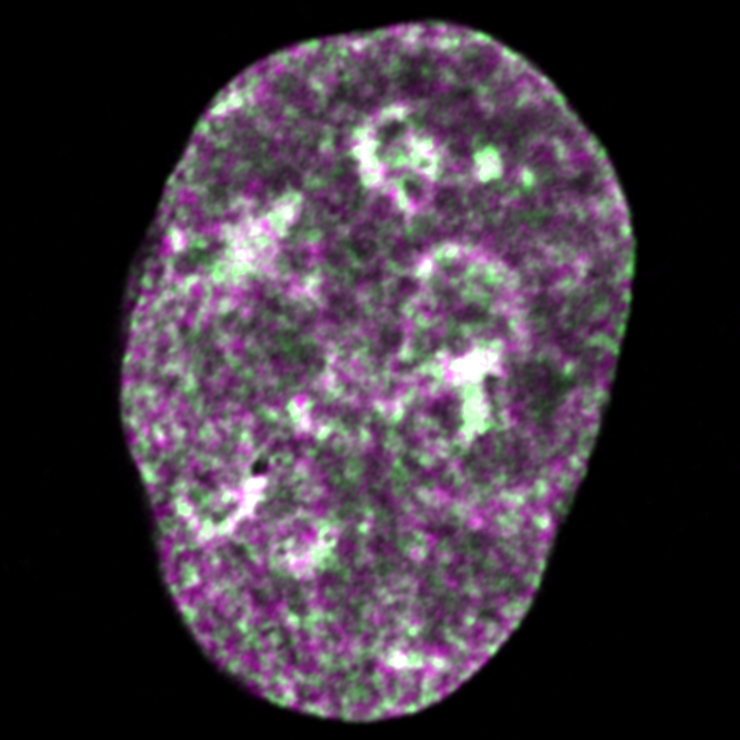

Supplement: Supplementary file 4 — Source Data for Figure 1 [file EMBJ-42-e113475-s007.zip › sd_figure1/panel_g/whole_cell/RGB_220202_5388_2056_smc4_dep_c1_rep1_hemi_zoom4_8-03-101.tif_registered_slice29_8bit_rotated_cropped_hoechst_edu.tif.tif]

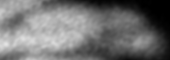

Supplement: Supplementary file 4 — Source Data for Figure 1 [file EMBJ-42-e113475-s007.zip › sd_figure1/panel_h/inset/8bit_220207_5389_2045_nipbl_dep_c1_rep2_60min_stlc_hemi_zoom5-01-10.czi #3.tif_registered_slice37_8bit_inset_170-60_hoechst.tif]

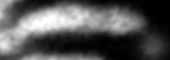

Supplement: Supplementary file 4 — Source Data for Figure 1 [file EMBJ-42-e113475-s007.zip › sd_figure1/panel_h/inset/C2-8bit_220207_5389_2045_nipbl_dep_c1_rep2_60min_stlc_hemi_zoom5-01-10.czi #3.tif_registered_slice37_8bit_inset_170-60_edu.tif]

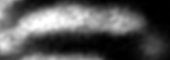

Supplement: Supplementary file 4 — Source Data for Figure 1 [file EMBJ-42-e113475-s007.zip › sd_figure1/panel_h/inset/RGB_220207_5389_2045_nipbl_dep_c1_rep2_60min_stlc_hemi_zoom5-01-10.czi #3.tif_registered_slice37_8bit_inset_170-60_edu_gray.tif]

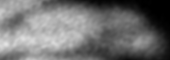

Supplement: Supplementary file 4 — Source Data for Figure 1 [file EMBJ-42-e113475-s007.zip › sd_figure1/panel_h/inset/RGB_220207_5389_2045_nipbl_dep_c1_rep2_60min_stlc_hemi_zoom5-01-10.czi #3.tif_registered_slice37_8bit_inset_170-60_hoechst_gray.tif]

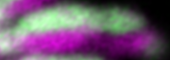

Supplement: Supplementary file 4 — Source Data for Figure 1 [file EMBJ-42-e113475-s007.zip › sd_figure1/panel_h/inset/RGB_220207_5389_2045_nipbl_dep_c1_rep2_60min_stlc_hemi_zoom5-01-10.czi #3.tif_registered_slice37_inset_170-60_hoechst_edu.tif.tif]

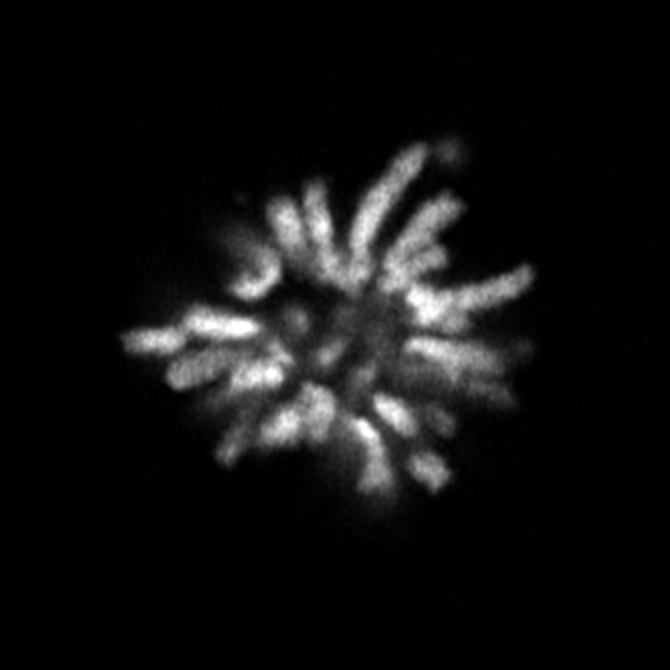

Supplement: Supplementary file 4 — Source Data for Figure 1 [file EMBJ-42-e113475-s007.zip › sd_figure1/panel_h/whole_cell/16bit_220207_5389_2045_nipbl_dep_c1_rep2_60min_stlc_hemi_zoom5-01-10.czi #3.tif_registered_slice37_16bit_hoechst_edu.tif]

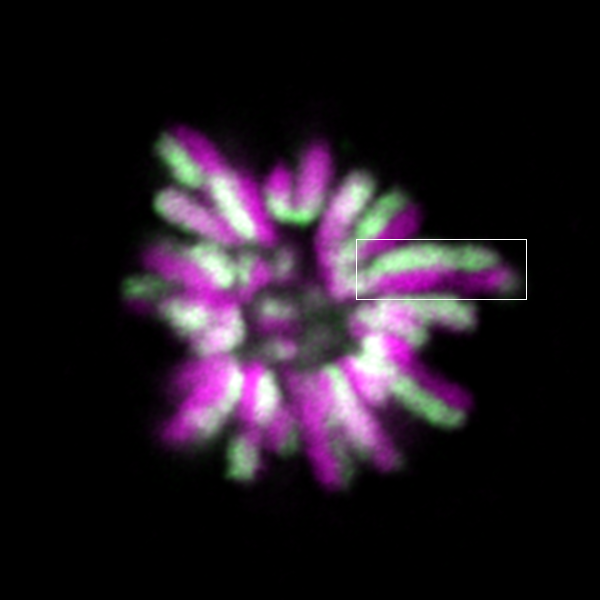

Supplement: Supplementary file 4 — Source Data for Figure 1 [file EMBJ-42-e113475-s007.zip › sd_figure1/panel_h/whole_cell/RGB_220207_5389_2045_nipbl_dep_c1_rep2_60min_stlc_hemi_zoom5-01-10.czi #3.tif_registered_slice37_hoechst_edu_draw_roi.tif]

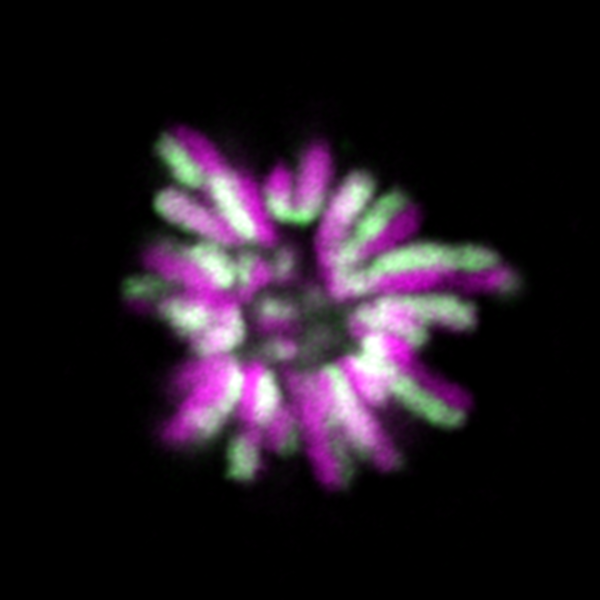

Supplement: Supplementary file 4 — Source Data for Figure 1 [file EMBJ-42-e113475-s007.zip › sd_figure1/panel_h/whole_cell/RGB_220207_5389_2045_nipbl_dep_c1_rep2_60min_stlc_hemi_zoom5-01-10.czi #3.tif_registered_slice37_rotated_cropped_hoechst_edu.tif (RGB).tif]

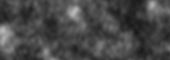

Supplement: Supplementary file 4 — Source Data for Figure 1 [file EMBJ-42-e113475-s007.zip › sd_figure1/panel_i/insets/8bit_220202_5388_2045_nipbl_dep_c2_rep2_hemi_zoom4_8-09-169.czi #2.tif_registered_slicd33_edu_inset_gray.tif]

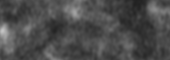

Supplement: Supplementary file 4 — Source Data for Figure 1 [file EMBJ-42-e113475-s007.zip › sd_figure1/panel_i/insets/8bit_220202_5388_2045_nipbl_dep_c2_rep2_hemi_zoom4_8-09-169.czi #2.tif_registered_slicd33_hoechst_inset_gray.tif]

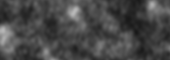

Supplement: Supplementary file 4 — Source Data for Figure 1 [file EMBJ-42-e113475-s007.zip › sd_figure1/panel_i/insets/RGB_220202_5388_2045_nipbl_dep_c2_rep2_hemi_zoom4_8-09-169.czi #2.tif_registered_slicd33_edu_inset_gray_Rgb.tif]

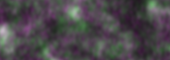

Supplement: Supplementary file 4 — Source Data for Figure 1 [file EMBJ-42-e113475-s007.zip › sd_figure1/panel_i/insets/RGB_220202_5388_2045_nipbl_dep_c2_rep2_hemi_zoom4_8-09-169.czi #2.tif_registered_slicd33_hoechst_edu_inset_170x60.tif.tif]

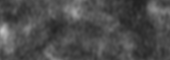

Supplement: Supplementary file 4 — Source Data for Figure 1 [file EMBJ-42-e113475-s007.zip › sd_figure1/panel_i/insets/RGB_220202_5388_2045_nipbl_dep_c2_rep2_hemi_zoom4_8-09-169.czi #2.tif_registered_slicd33_hoechst_inset_gray_rgb.tif]

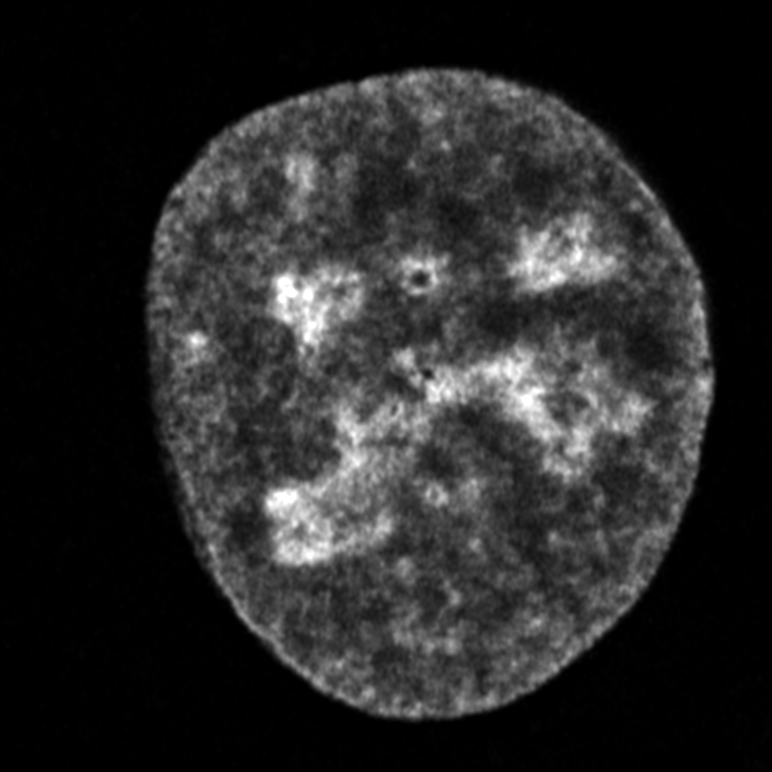

Supplement: Supplementary file 4 — Source Data for Figure 1 [file EMBJ-42-e113475-s007.zip › sd_figure1/panel_i/whole_cell/16bit_220202_5388_2045_nipbl_dep_c2_rep2_hemi_zoom4_8-09-169.czi #2.tif_registered_slicd33_hoechst_edu.tif]

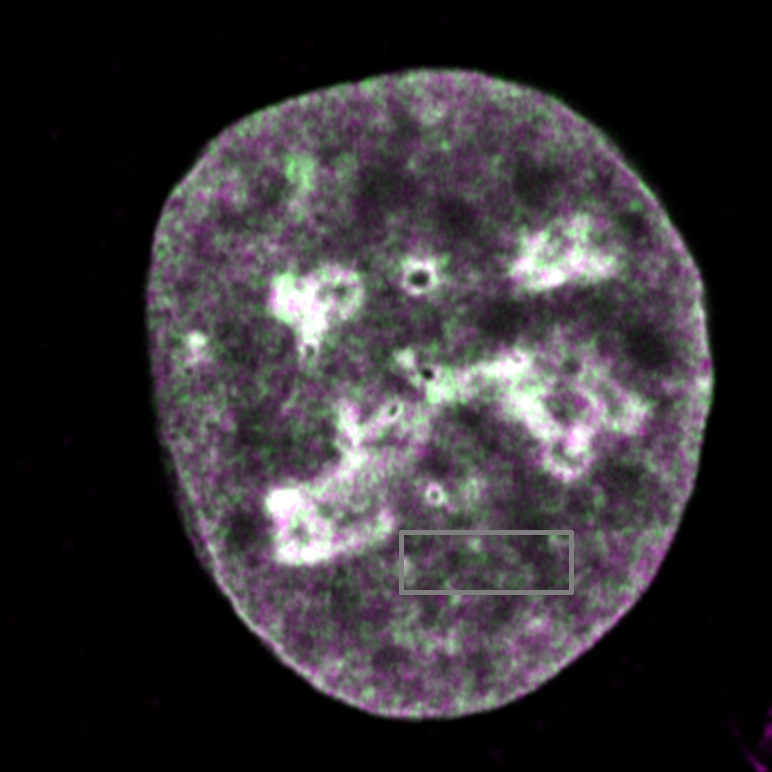

Supplement: Supplementary file 4 — Source Data for Figure 1 [file EMBJ-42-e113475-s007.zip › sd_figure1/panel_i/whole_cell/RGB_220202_5388_2045_nipbl_dep_c2_rep2_hemi_zoom4_8-09-169.czi #2.tif_registered_slicd33_hoechst_edu.tif (RGB)_draw_roi.tif]

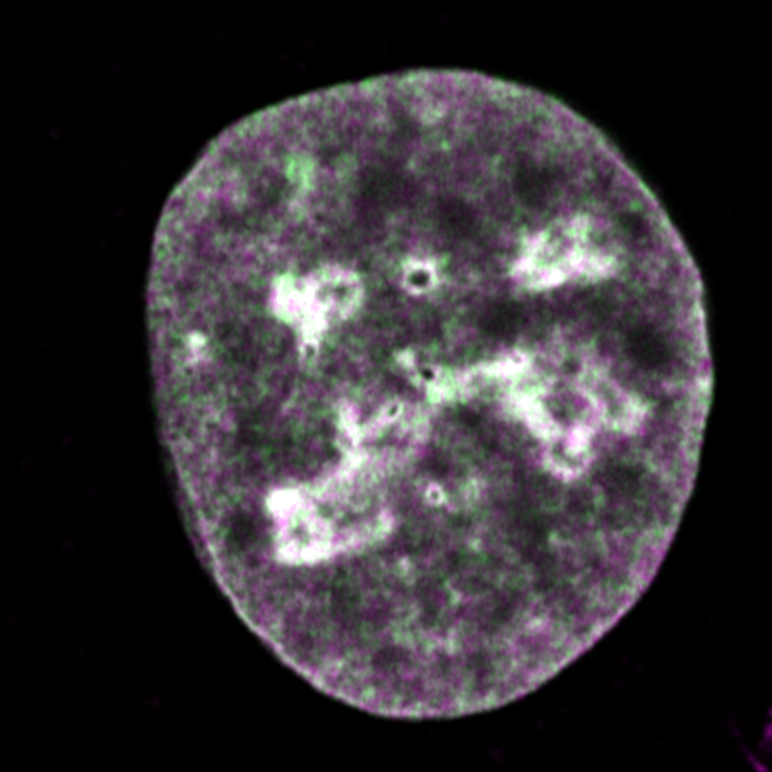

Supplement: Supplementary file 4 — Source Data for Figure 1 [file EMBJ-42-e113475-s007.zip › sd_figure1/panel_i/whole_cell/RGB_220202_5388_2045_nipbl_dep_c2_rep2_hemi_zoom4_8-09-169.czi #2.tif_registered_slicd33_hoechst_edu.tif.tif]

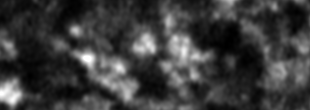

Supplement: Supplementary file 5 — Source Data for Figure 2 [file EMBJ-42-e113475-s004.zip › sd_figure2/panel_a/insets/16bit_220211_5389_2096_c2_rep1_ctrl_hemi_g2_zoom4_8-01-20.tif_registered_slice27_310x110_hoechst_edu.tif]

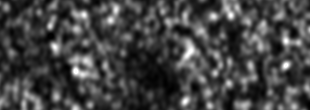

Supplement: Supplementary file 5 — Source Data for Figure 2 [file EMBJ-42-e113475-s004.zip › sd_figure2/panel_a/insets/16bit_220211_5389_2096_c2_rep1_ctrl_hemi_g2_zoom4_8-01-20.tif_registered_slice27_310x110_scc1.tif]

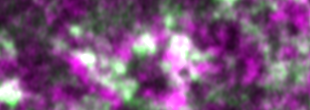

Supplement: Supplementary file 5 — Source Data for Figure 2 [file EMBJ-42-e113475-s004.zip › sd_figure2/panel_a/insets/RGB_220211_5389_2096_c2_rep1_ctrl_hemi_g2_zoom4_8-01-20.tif_registered_slice27_310x110_hoechst_edu.tif.tif]

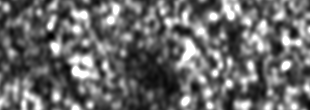

Supplement: Supplementary file 5 — Source Data for Figure 2 [file EMBJ-42-e113475-s004.zip › sd_figure2/panel_a/insets/RGB_220211_5389_2096_c2_rep1_ctrl_hemi_g2_zoom4_8-01-20.tif_registered_slice27_310x110_scc1.tif]

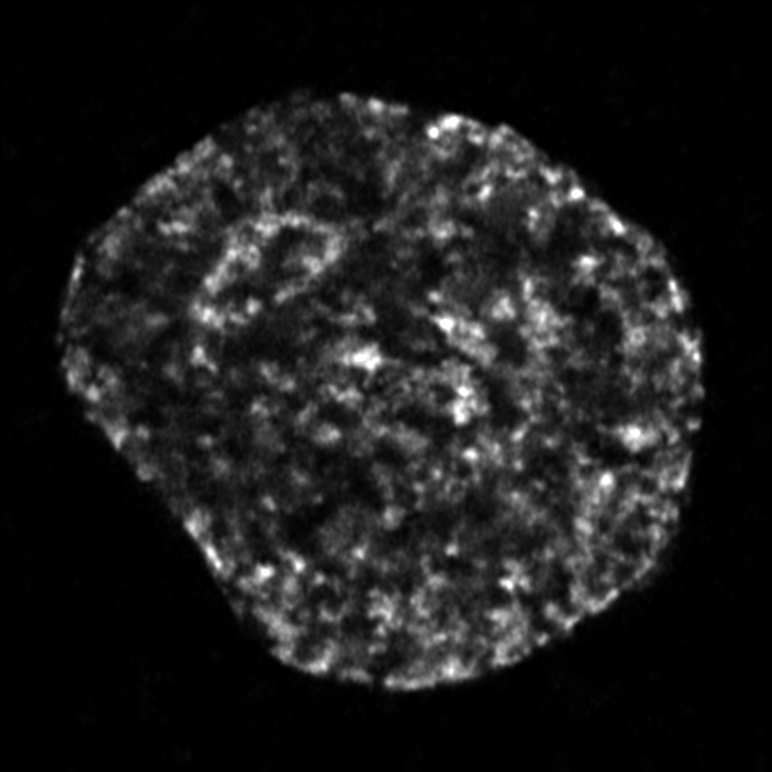

Supplement: Supplementary file 5 — Source Data for Figure 2 [file EMBJ-42-e113475-s004.zip › sd_figure2/panel_a/whole_cell/16bit_220211_5389_2096_c2_rep1_ctrl_hemi_g2_zoom4_8-01-20.tif_registered_slice28_hoechst_edu.tif]

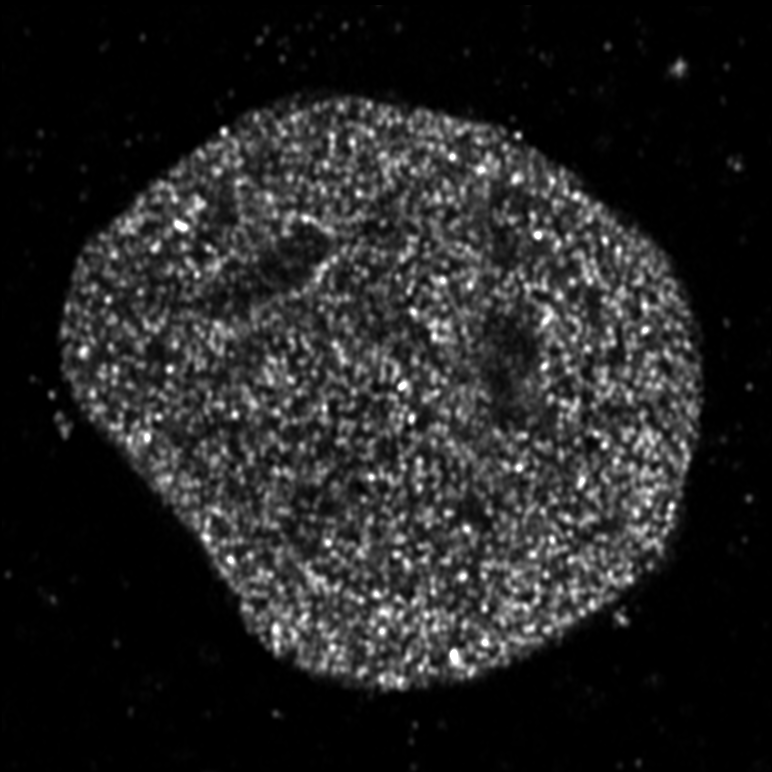

Supplement: Supplementary file 5 — Source Data for Figure 2 [file EMBJ-42-e113475-s004.zip › sd_figure2/panel_a/whole_cell/16bit_220211_5389_2096_c2_rep1_ctrl_hemi_g2_zoom4_8-01-20.tif_registered_slice28_scc1.tif]

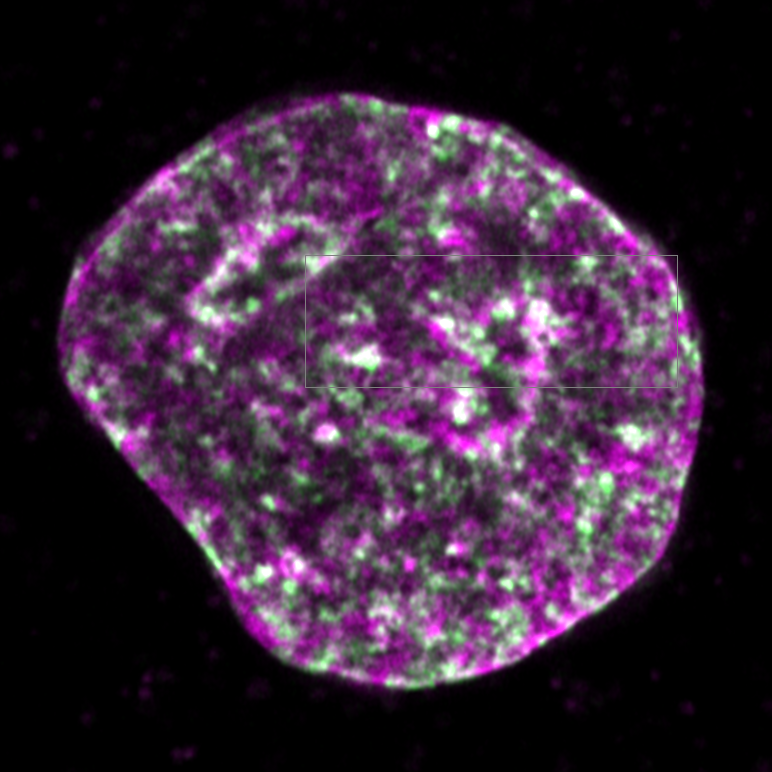

Supplement: Supplementary file 5 — Source Data for Figure 2 [file EMBJ-42-e113475-s004.zip › sd_figure2/panel_a/whole_cell/RGB_220211_5389_2096_c2_rep1_ctrl_hemi_g2_zoom4_8-01-20.tif_registered_slice_27_8bit_hoechst_edu.tif (RGB)_draw_roi.tif]

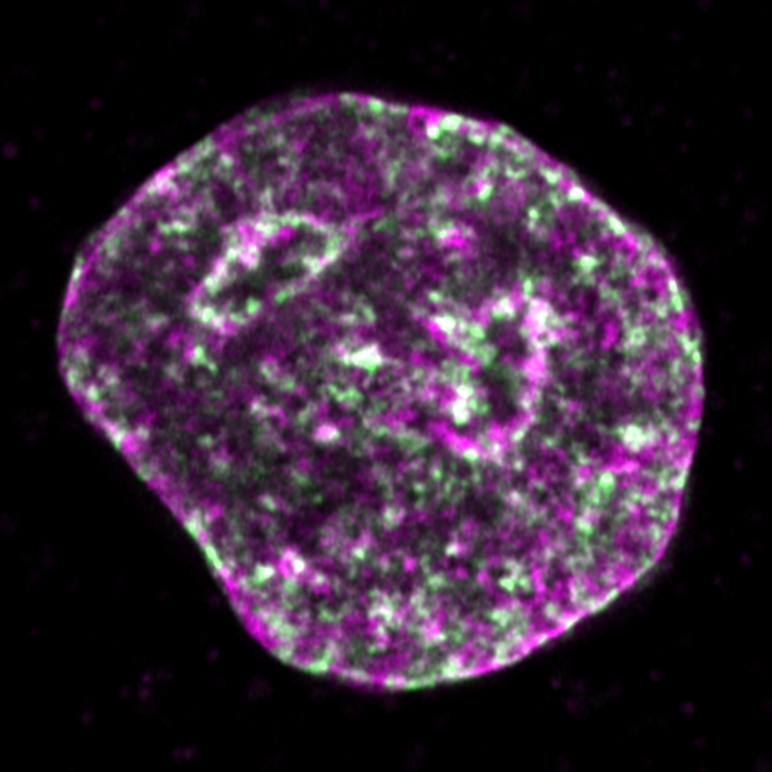

Supplement: Supplementary file 5 — Source Data for Figure 2 [file EMBJ-42-e113475-s004.zip › sd_figure2/panel_a/whole_cell/RGB_220211_5389_2096_c2_rep1_ctrl_hemi_g2_zoom4_8-01-20.tif_registered_slice28_hoechst_edu.tif (RGB).tif]

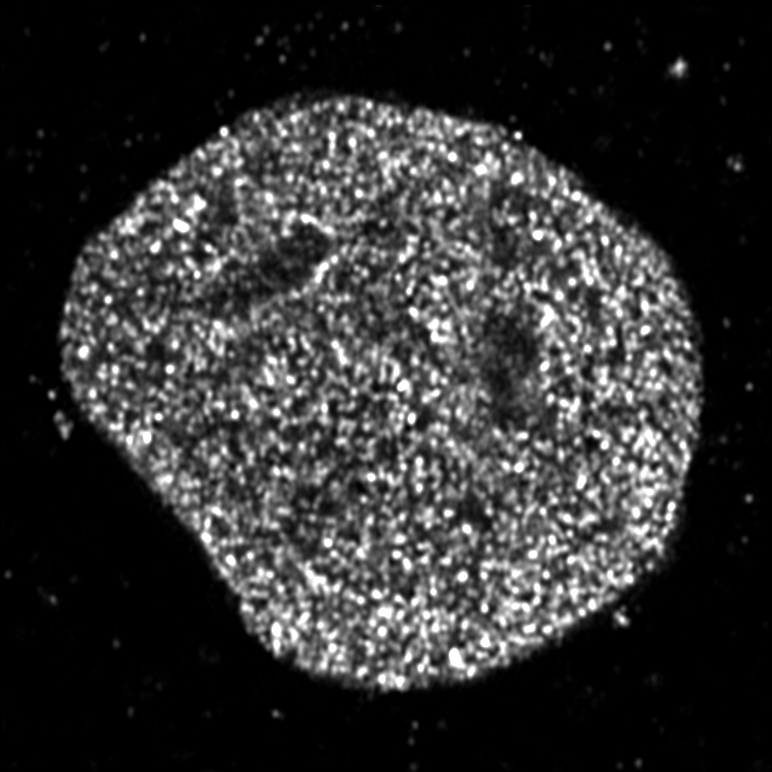

Supplement: Supplementary file 5 — Source Data for Figure 2 [file EMBJ-42-e113475-s004.zip › sd_figure2/panel_a/whole_cell/RGB_220211_5389_2096_c2_rep1_ctrl_hemi_g2_zoom4_8-01-20.tif_registered_slice28_scc1.tif]

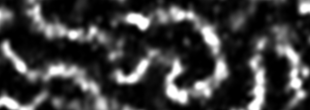

Supplement: Supplementary file 5 — Source Data for Figure 2 [file EMBJ-42-e113475-s004.zip › sd_figure2/panel_b/insets/8bit_220211_5389_2096_c2_rep2_wapl_dep_on_hemi_g2_zoom4_8-05-74.czi #1.tif_registered_slice22_8bit_rotated_inset_310x110_scc1.tif]

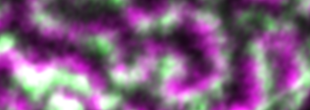

Supplement: Supplementary file 5 — Source Data for Figure 2 [file EMBJ-42-e113475-s004.zip › sd_figure2/panel_b/insets/RGB_220211_5389_2096_c2_rep2_wapl_dep_on_hemi_g2_zoom4_8-05-74.czi #1.tif_registered_slice22_8bit_rotated_inset_310x110_hoechst_edu.tif.tif]

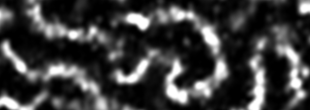

Supplement: Supplementary file 5 — Source Data for Figure 2 [file EMBJ-42-e113475-s004.zip › sd_figure2/panel_b/insets/RGB_220211_5389_2096_c2_rep2_wapl_dep_on_hemi_g2_zoom4_8-05-74.czi #1.tif_registered_slice22_8bit_rotated_inset_310x110_scc1.tif]

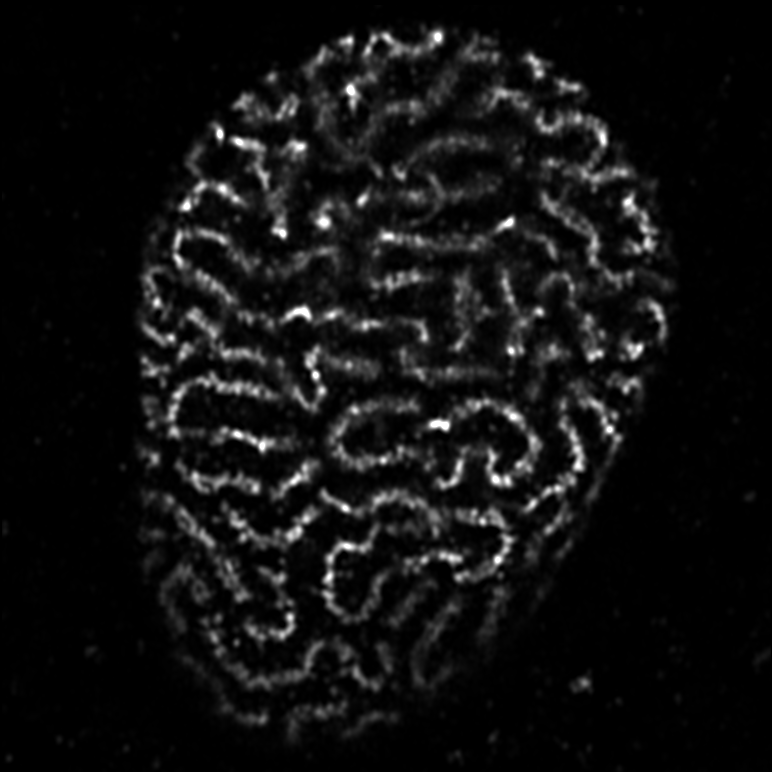

Supplement: Supplementary file 5 — Source Data for Figure 2 [file EMBJ-42-e113475-s004.zip › sd_figure2/panel_b/whole_cell/16bit_220211_5389_2096_c2_rep2_wapl_dep_on_hemi_g2_zoom4_8-05-74.czi #1.tif_registered_all.tif]

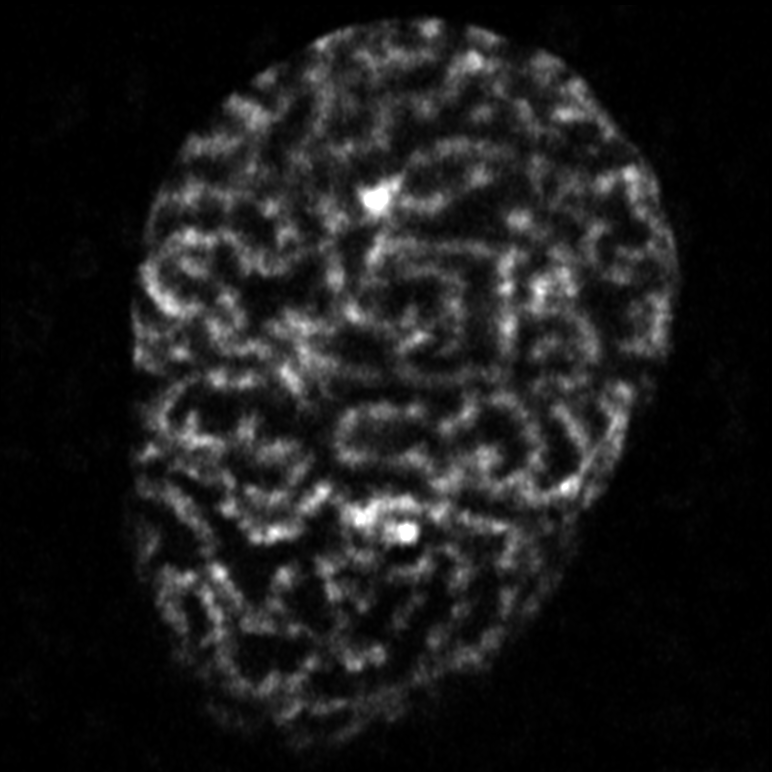

Supplement: Supplementary file 5 — Source Data for Figure 2 [file EMBJ-42-e113475-s004.zip › sd_figure2/panel_b/whole_cell/16bit_220211_5389_2096_c2_rep2_wapl_dep_on_hemi_g2_zoom4_8-05-74.czi #1.tif_registered_hoechst_ed.tif]

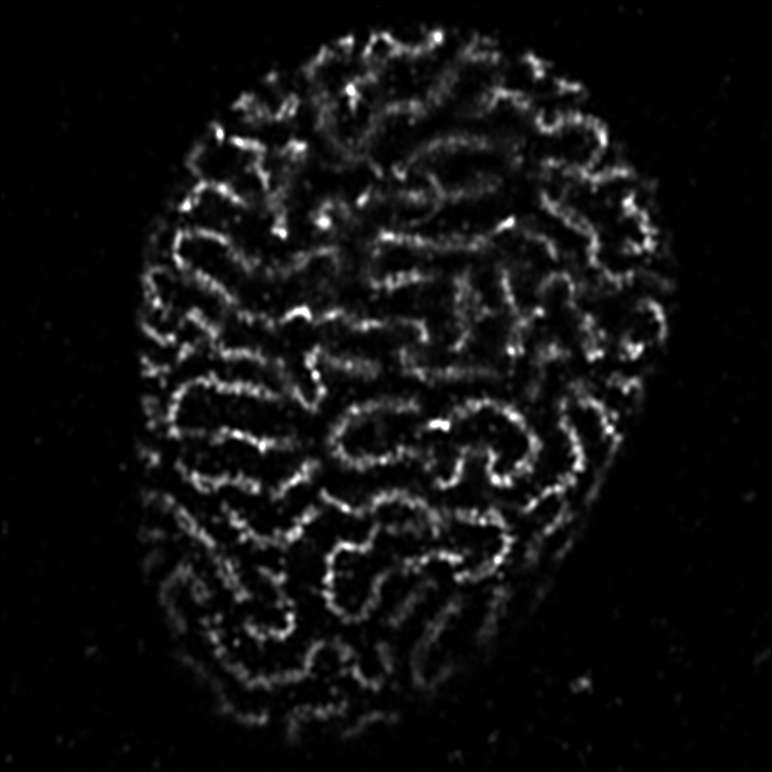

Supplement: Supplementary file 5 — Source Data for Figure 2 [file EMBJ-42-e113475-s004.zip › sd_figure2/panel_b/whole_cell/16bit_220211_5389_2096_c2_rep2_wapl_dep_on_hemi_g2_zoom4_8-05-74.czi #1.tif_registered_scc1.tif]

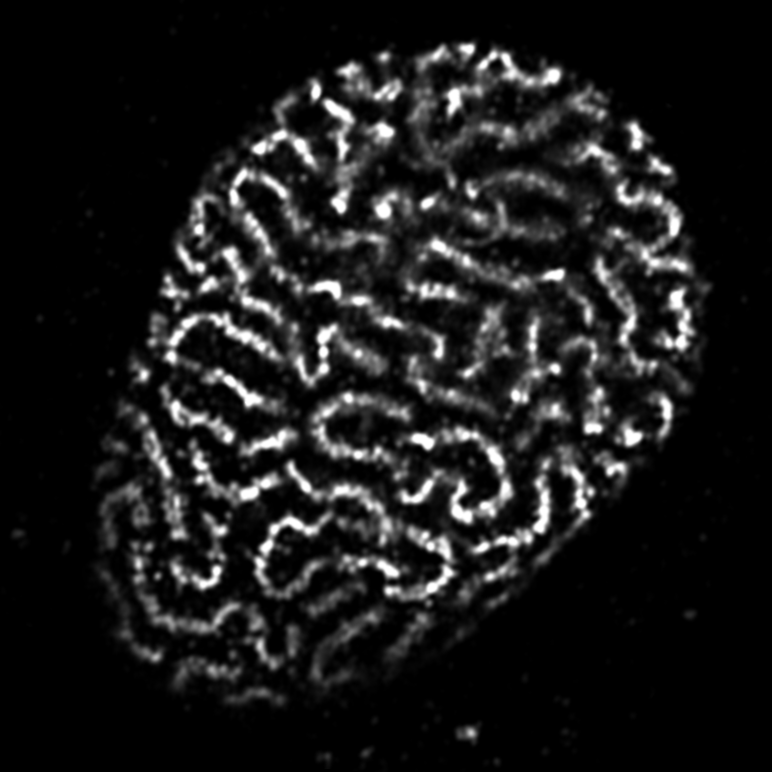

Supplement: Supplementary file 5 — Source Data for Figure 2 [file EMBJ-42-e113475-s004.zip › sd_figure2/panel_b/whole_cell/8bit_220211_5389_2096_c2_rep2_wapl_dep_on_hemi_g2_zoom4_8-05-74.czi #1.tif_registered_slice22_rotated_scc1.tif]

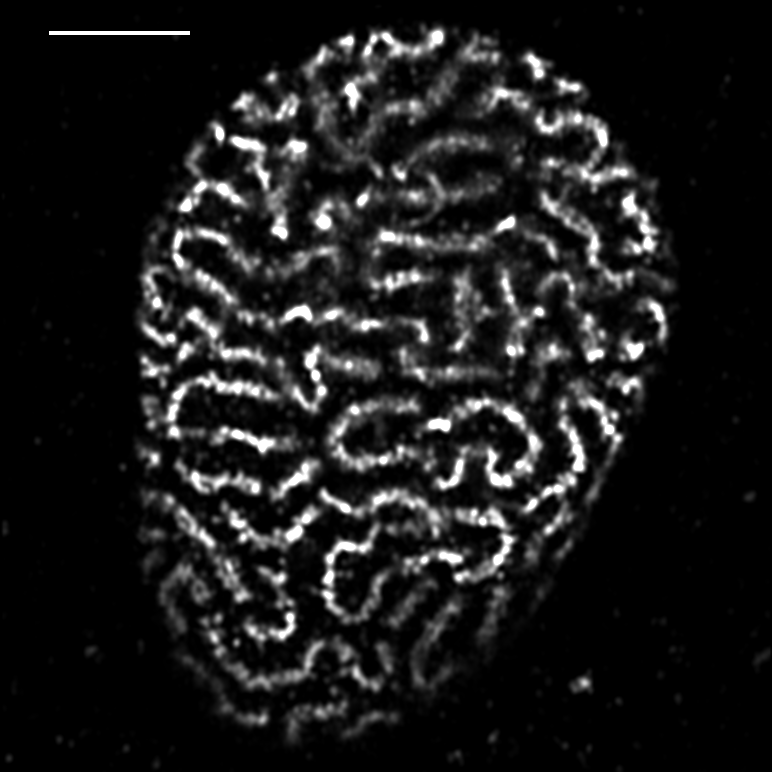

Supplement: Supplementary file 5 — Source Data for Figure 2 [file EMBJ-42-e113475-s004.zip › sd_figure2/panel_b/whole_cell/8bit_220211_5389_2096_c2_rep2_wapl_dep_on_hemi_g2_zoom4_8-05-74.czi #1.tif_registered_slice22_scc1_scalebar.tif]

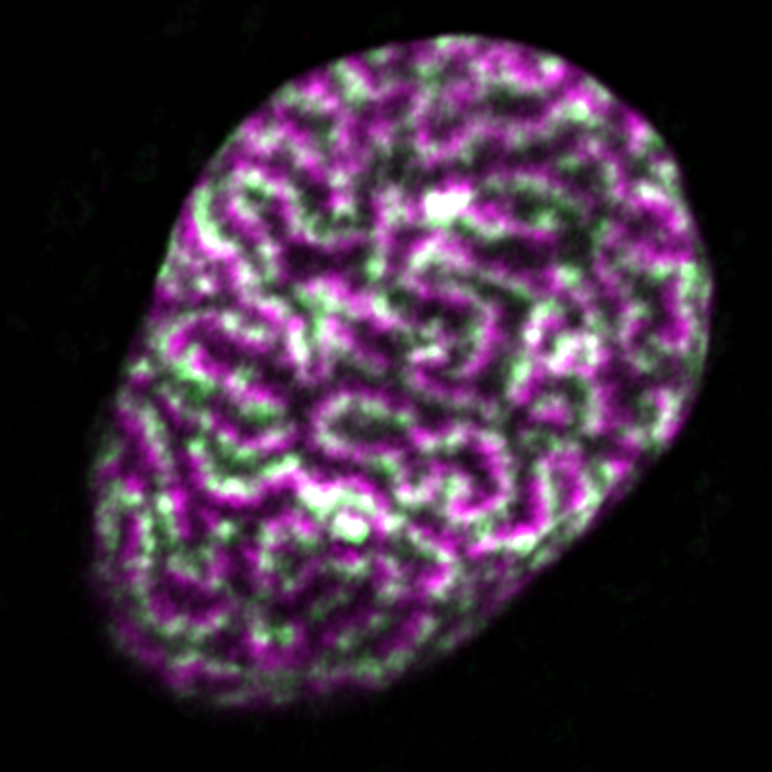

Supplement: Supplementary file 5 — Source Data for Figure 2 [file EMBJ-42-e113475-s004.zip › sd_figure2/panel_b/whole_cell/RGB_220211_5389_2096_c2_rep2_wapl_dep_on_hemi_g2_zoom4_8-05-74.czi #1.tif_registered_slice22_8bit_rotated_edu_hoechst.tif.tif]

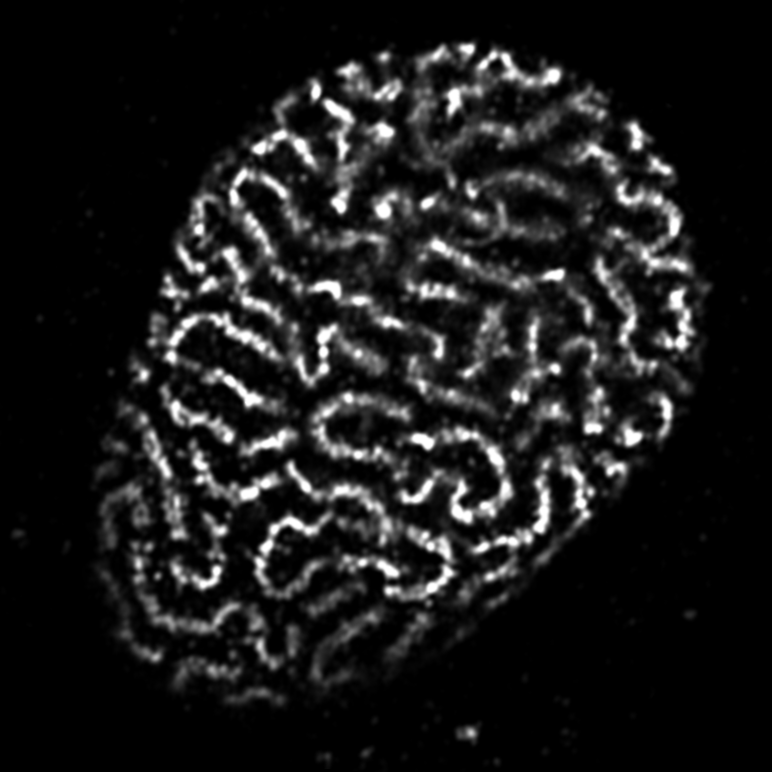

Supplement: Supplementary file 5 — Source Data for Figure 2 [file EMBJ-42-e113475-s004.zip › sd_figure2/panel_b/whole_cell/RGB_220211_5389_2096_c2_rep2_wapl_dep_on_hemi_g2_zoom4_8-05-74.czi #1.tif_registered_slice22_8bit_rotated_scc1.tif]

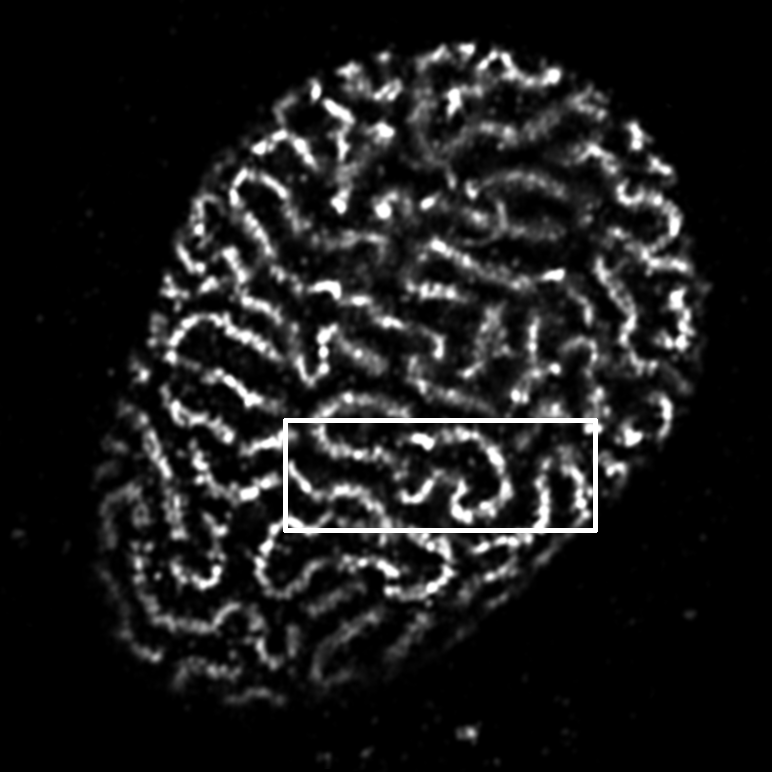

Supplement: Supplementary file 5 — Source Data for Figure 2 [file EMBJ-42-e113475-s004.zip › sd_figure2/panel_b/whole_cell/RGB_220211_5389_2096_c2_rep2_wapl_dep_on_hemi_g2_zoom4_8-05-74.czi #1.tif_registered_slice22_rotated.tif_draw_roi.tif]

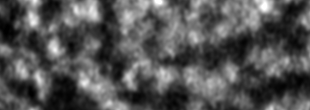

Supplement: Supplementary file 5 — Source Data for Figure 2 [file EMBJ-42-e113475-s004.zip › sd_figure2/panel_c/insets/16bit_220211_5389_2096_c2_rep2sor_dep_on_hemi_g2_zoom4_8-01-49.tif_registered_slice23_inset_310x110_hoechst_edu.tif]

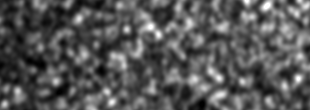

Supplement: Supplementary file 5 — Source Data for Figure 2 [file EMBJ-42-e113475-s004.zip › sd_figure2/panel_c/insets/16bit_220211_5389_2096_c2_rep2sor_dep_on_hemi_g2_zoom4_8-01-49.tif_registered_slice23_inset_310x110_scc1.tif]

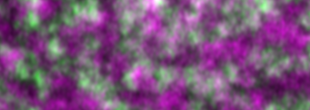

Supplement: Supplementary file 5 — Source Data for Figure 2 [file EMBJ-42-e113475-s004.zip › sd_figure2/panel_c/insets/RGB_220211_5389_2096_c2_rep2sor_dep_on_hemi_g2_zoom4_8-01-49.tif_registered_slice23_inset_310x110_hoechst_edu.tif.tif]

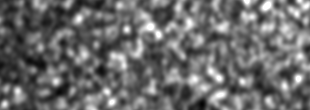

Supplement: Supplementary file 5 — Source Data for Figure 2 [file EMBJ-42-e113475-s004.zip › sd_figure2/panel_c/insets/RGB_220211_5389_2096_c2_rep2sor_dep_on_hemi_g2_zoom4_8-01-49.tif_registered_slice23_inset_310x110_scc1.tif]

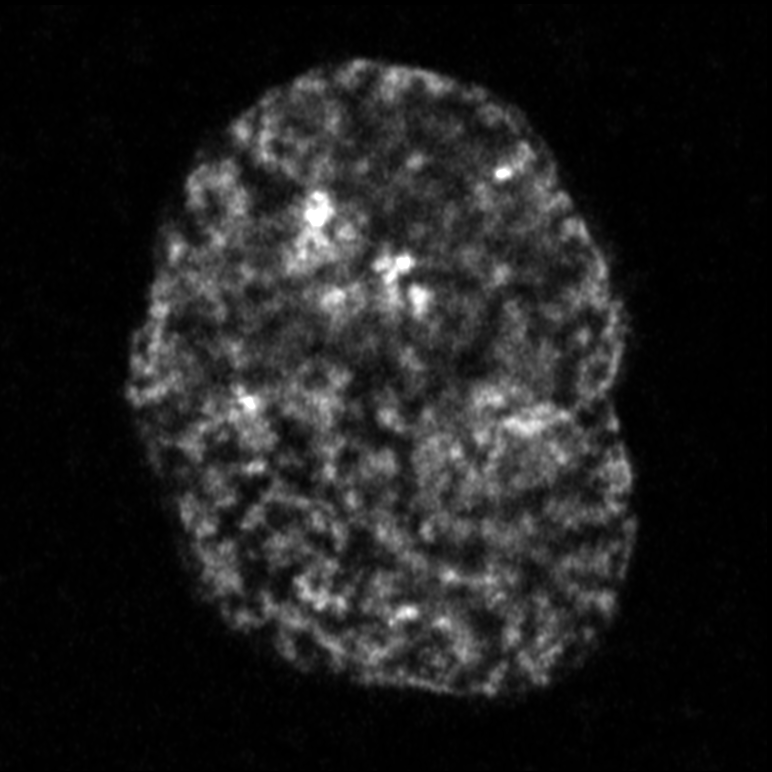

Supplement: Supplementary file 5 — Source Data for Figure 2 [file EMBJ-42-e113475-s004.zip › sd_figure2/panel_c/whole_cell/16bit_220211_5389_2096_c2_rep2sor_dep_on_hemi_g2_zoom4_8-01-49.tif_registered_slice24_hoechst_edu.tif]

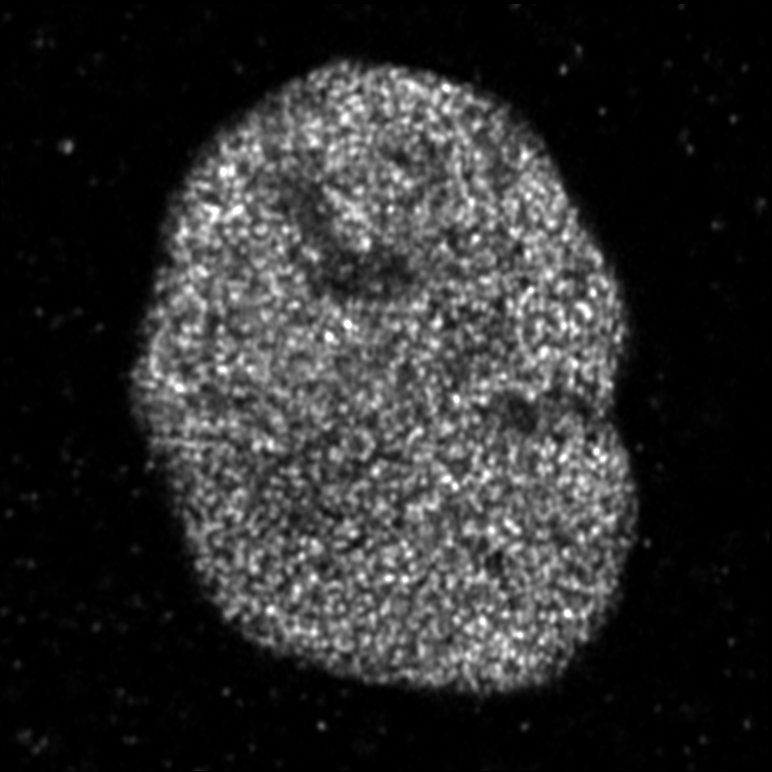

Supplement: Supplementary file 5 — Source Data for Figure 2 [file EMBJ-42-e113475-s004.zip › sd_figure2/panel_c/whole_cell/16bit_220211_5389_2096_c2_rep2sor_dep_on_hemi_g2_zoom4_8-01-49.tif_registered_slice24_scc1.tif]

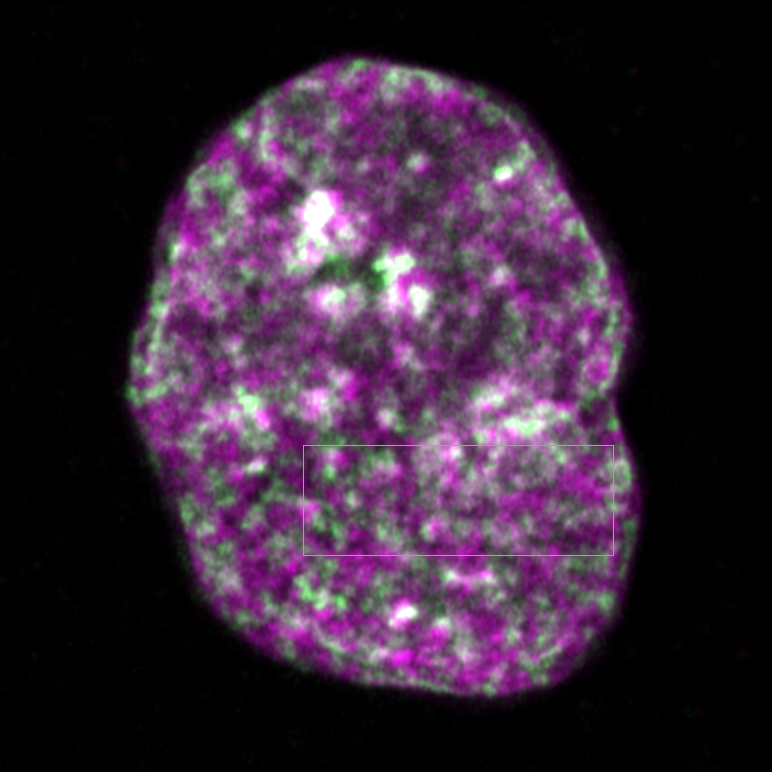

Supplement: Supplementary file 5 — Source Data for Figure 2 [file EMBJ-42-e113475-s004.zip › sd_figure2/panel_c/whole_cell/RGB_220211_5389_2096_c2_rep2sor_dep_on_hemi_g2_zoom4_8-01-49.tif_registered_slice24.tif (RGB)_draw_roi.tif]

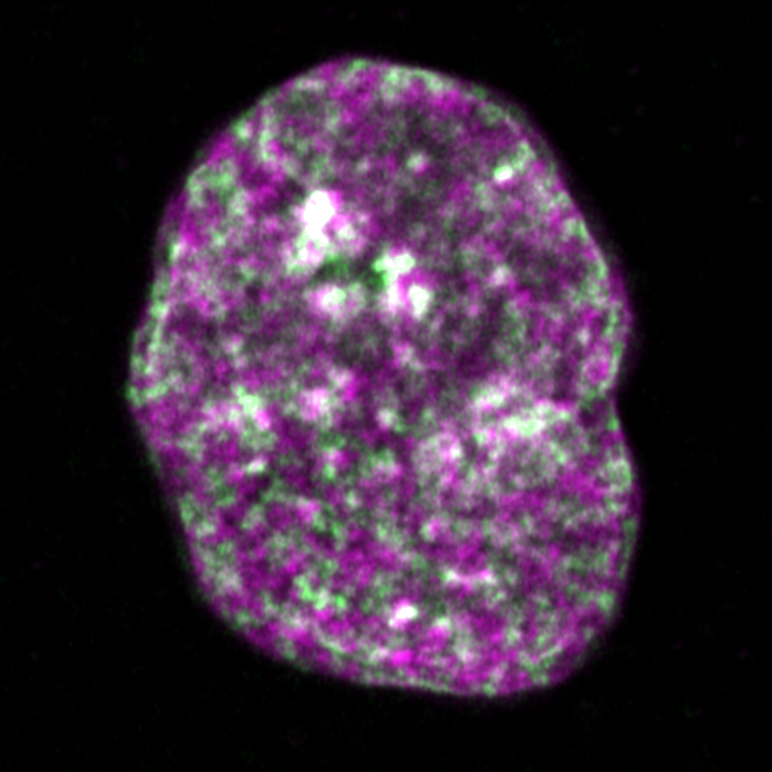

Supplement: Supplementary file 5 — Source Data for Figure 2 [file EMBJ-42-e113475-s004.zip › sd_figure2/panel_c/whole_cell/RGB_220211_5389_2096_c2_rep2sor_dep_on_hemi_g2_zoom4_8-01-49.tif_registered_slice24_hoechst_edu.tif (RGB).tif]

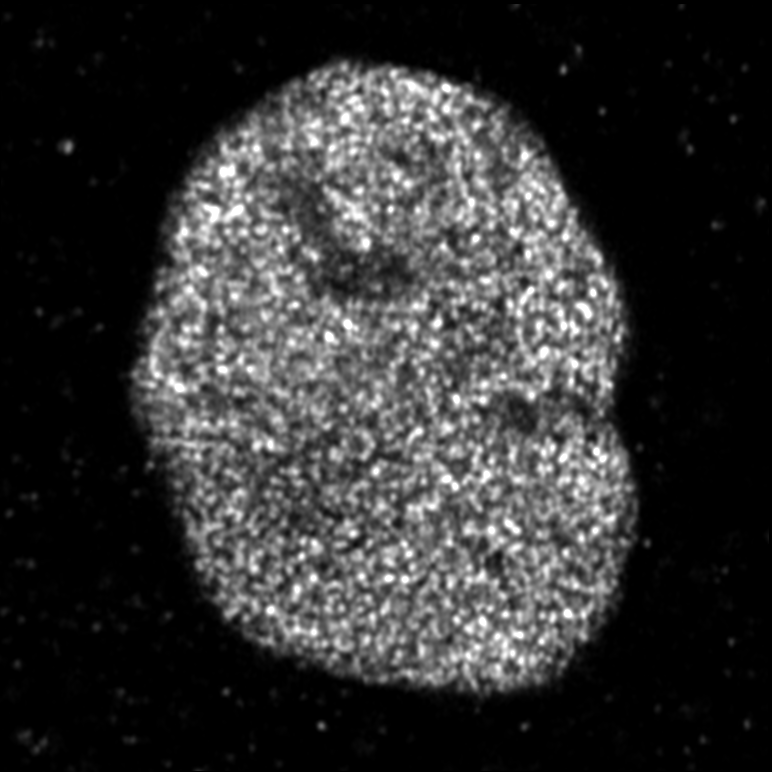

Supplement: Supplementary file 5 — Source Data for Figure 2 [file EMBJ-42-e113475-s004.zip › sd_figure2/panel_c/whole_cell/RGB_220211_5389_2096_c2_rep2sor_dep_on_hemi_g2_zoom4_8-01-49.tif_registered_slice24_scc1_rgb.tif]

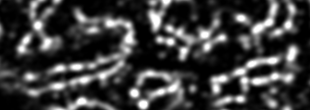

Supplement: Supplementary file 5 — Source Data for Figure 2 [file EMBJ-42-e113475-s004.zip › sd_figure2/panel_e/insets/8bit_220211_5389_2096_c2_rep1_wapl_sor_dep_on_hemi_g2_zoom4_8-02-38.czi #5.tif_registered_slice18_8bit_rotated2_730x730_all.tif]

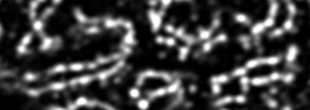

Supplement: Supplementary file 5 — Source Data for Figure 2 [file EMBJ-42-e113475-s004.zip › sd_figure2/panel_e/insets/8bit_220211_5389_2096_c2_rep1_wapl_sor_dep_on_hemi_g2_zoom4_8-02-38.czi #5.tif_registered_slice18_8bit_rotated2_730x730_scc1.tif]

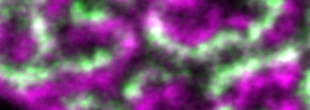

Supplement: Supplementary file 5 — Source Data for Figure 2 [file EMBJ-42-e113475-s004.zip › sd_figure2/panel_e/insets/RGB_220211_5389_2096_c2_rep1_wapl_sor_dep_on_hemi_g2_zoom4_8-02-38.czi #5.tif_registered_slice18_8bit_rotated2_730x730_hoechst_edu.tif (RGB).tif]
