# Supplementary figures and images for: Cohesin‐mediated DNA loop extrusion resolves sister chromatids in G2 phase (part 2 of 3)
Source: EMBO J. 2023 Jun 26;42(16):e113475. doi: 10.15252/embj.2023113475 (PMC10425840; doi:10.15252/embj.2023113475)

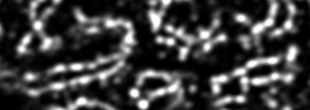

Supplement: Supplementary file 5 — Source Data for Figure 2 [file EMBJ-42-e113475-s004.zip › sd_figure2/panel_e/insets/RGB_220211_5389_2096_c2_rep1_wapl_sor_dep_on_hemi_g2_zoom4_8-02-38.czi #5.tif_registered_slice18_8bit_rotated2_730x730_scc1_rgb.tif]

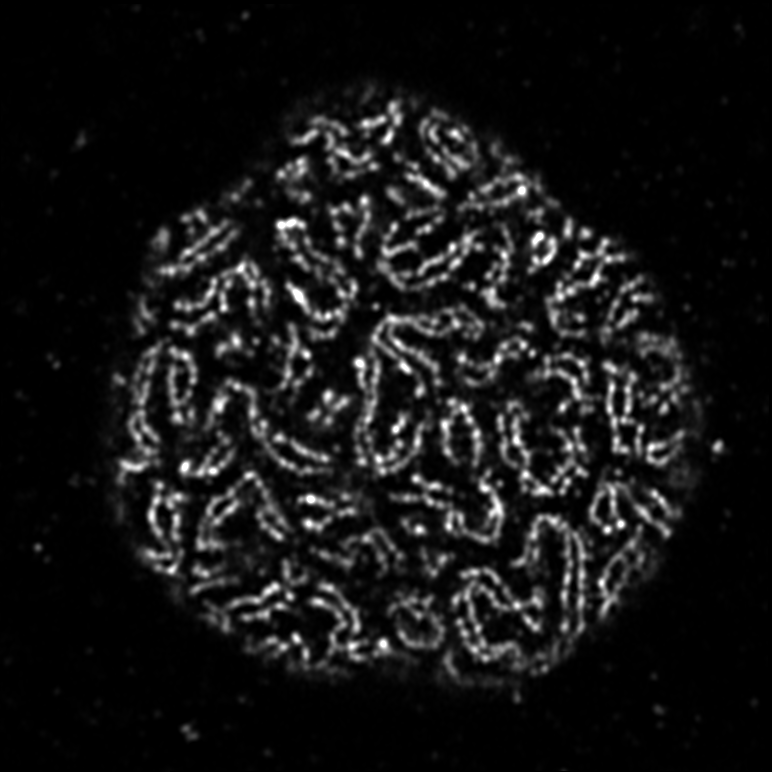

Supplement: Supplementary file 5 — Source Data for Figure 2 [file EMBJ-42-e113475-s004.zip › sd_figure2/panel_e/whole_cell/16bit_220211_5389_2096_c2_rep1_wapl_sor_dep_on_hemi_g2_zoom4_8-02-38.czi #5.tif_registered_slice18_all.tif]

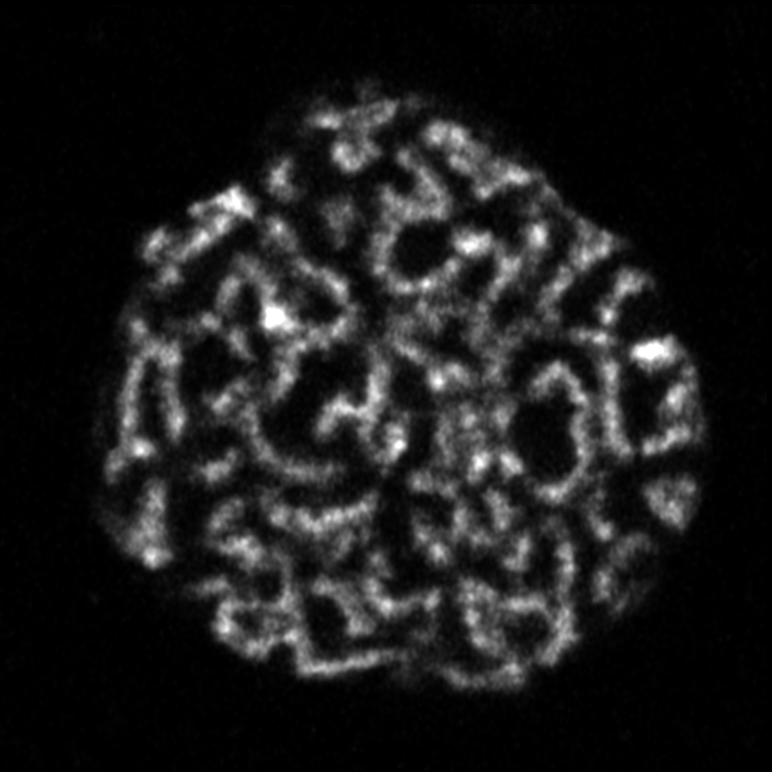

Supplement: Supplementary file 5 — Source Data for Figure 2 [file EMBJ-42-e113475-s004.zip › sd_figure2/panel_e/whole_cell/16bit_220211_5389_2096_c2_rep1_wapl_sor_dep_on_hemi_g2_zoom4_8-02-38.czi #5.tif_registered_slice18_hoechst_edu.tif]

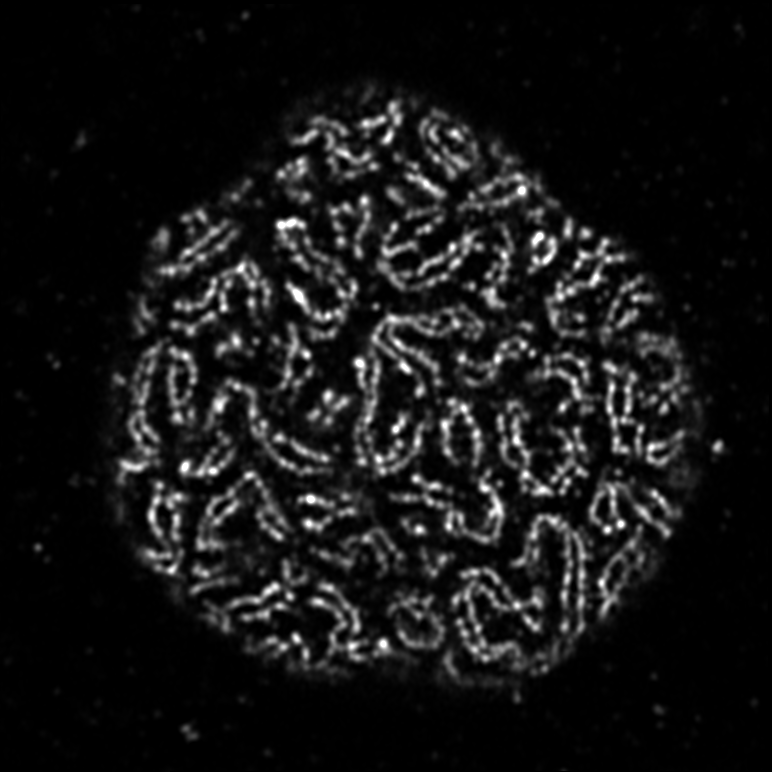

Supplement: Supplementary file 5 — Source Data for Figure 2 [file EMBJ-42-e113475-s004.zip › sd_figure2/panel_e/whole_cell/16bit_220211_5389_2096_c2_rep1_wapl_sor_dep_on_hemi_g2_zoom4_8-02-38.czi #5.tif_registered_slice18_scc1.tif]

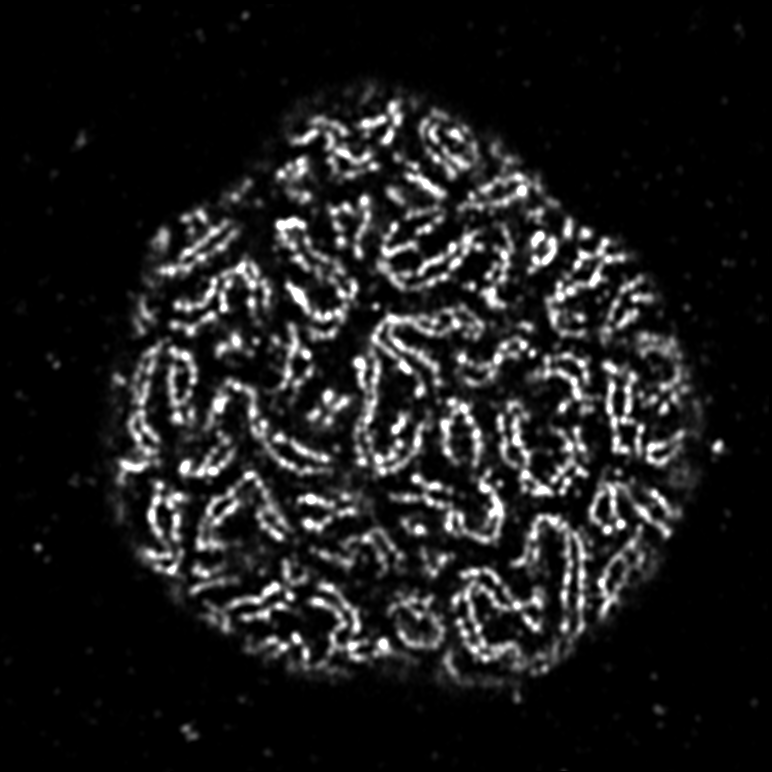

Supplement: Supplementary file 5 — Source Data for Figure 2 [file EMBJ-42-e113475-s004.zip › sd_figure2/panel_e/whole_cell/8bit_220211_5389_2096_c2_rep1_wapl_sor_dep_on_hemi_g2_zoom4_8-02-38.czi #5.tif_registered_slice18_8bit_all.tif]

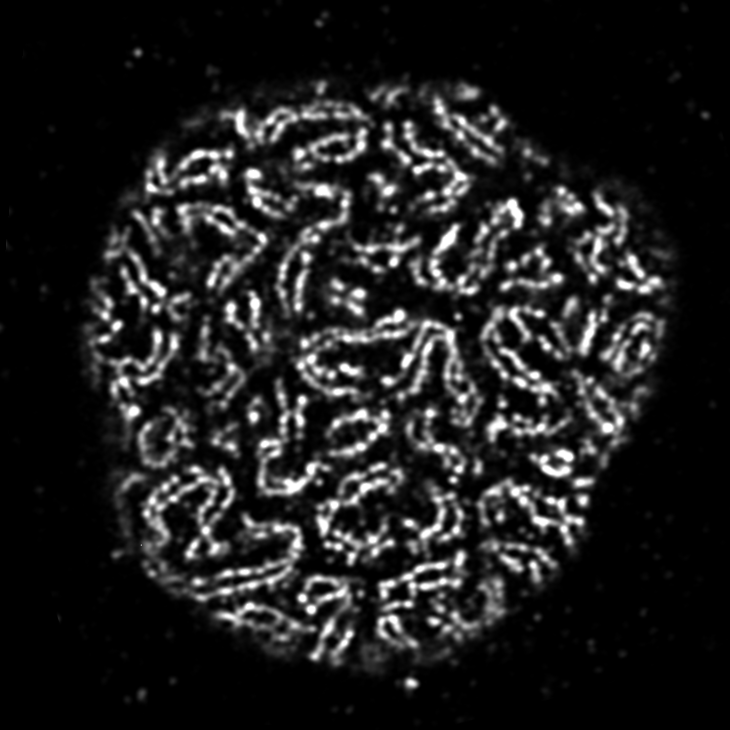

Supplement: Supplementary file 5 — Source Data for Figure 2 [file EMBJ-42-e113475-s004.zip › sd_figure2/panel_e/whole_cell/8bit_220211_5389_2096_c2_rep1_wapl_sor_dep_on_hemi_g2_zoom4_8-02-38.czi #5.tif_registered_slice18_8bit_rotated_cropped_all.tif]

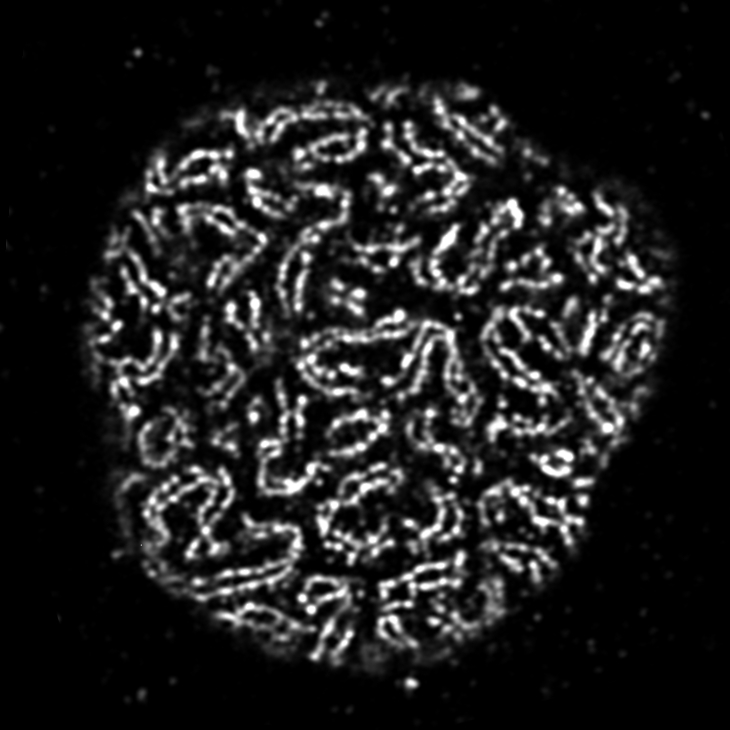

Supplement: Supplementary file 5 — Source Data for Figure 2 [file EMBJ-42-e113475-s004.zip › sd_figure2/panel_e/whole_cell/8bit_220211_5389_2096_c2_rep1_wapl_sor_dep_on_hemi_g2_zoom4_8-02-38.czi #5.tif_registered_slice18_8bit_rotated_cropped_scc1.tif]

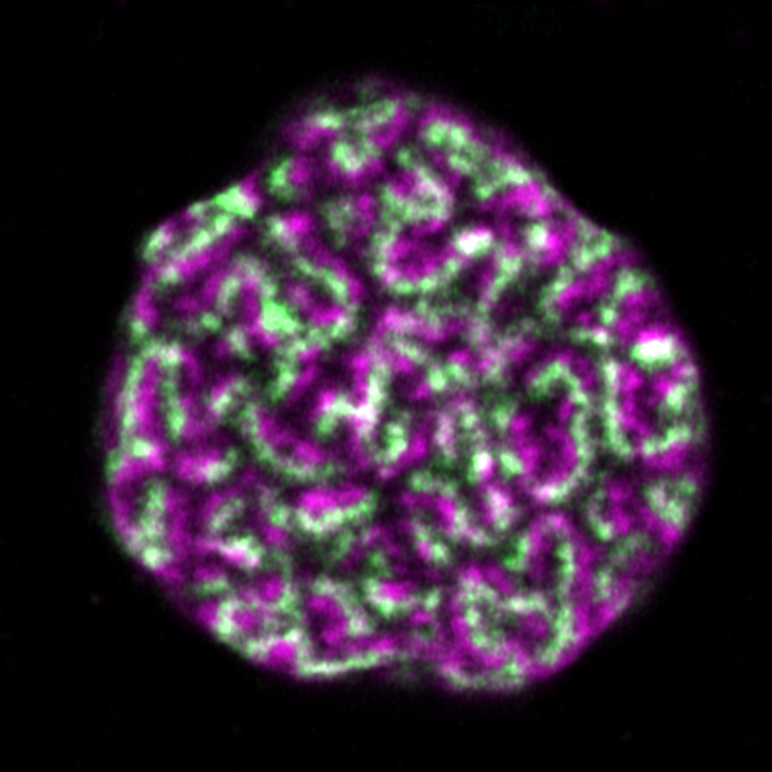

Supplement: Supplementary file 5 — Source Data for Figure 2 [file EMBJ-42-e113475-s004.zip › sd_figure2/panel_e/whole_cell/RGB_220211_5389_2096_c2_rep1_wapl_sor_dep_on_hemi_g2_zoom4_8-02-38.czi #5.tif_registered_slice18_8bit_hoechst_edu.tif.tif]

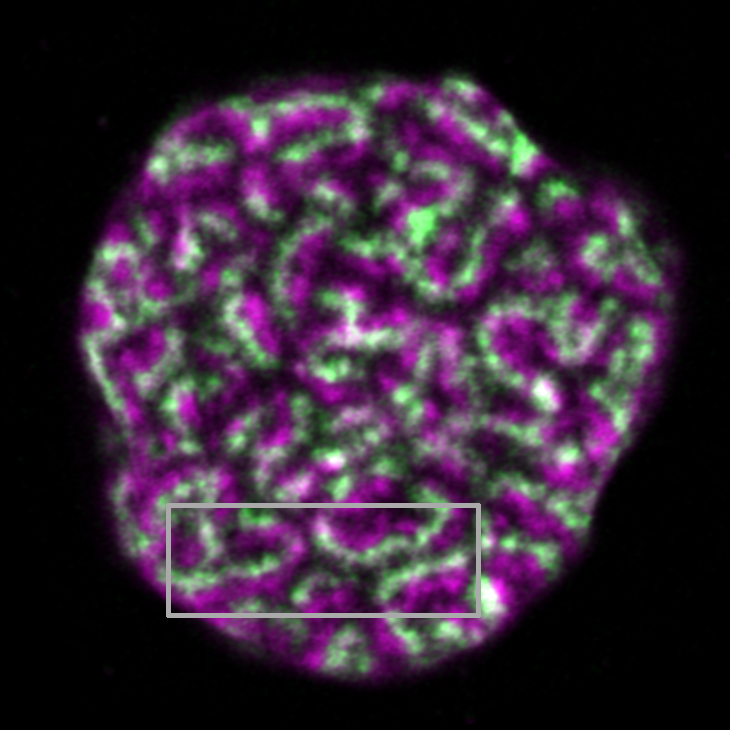

Supplement: Supplementary file 5 — Source Data for Figure 2 [file EMBJ-42-e113475-s004.zip › sd_figure2/panel_e/whole_cell/RGB_220211_5389_2096_c2_rep1_wapl_sor_dep_on_hemi_g2_zoom4_8-02-38.czi #5.tif_registered_slice18_8bit_rotated2_730x730.tif_draw_roi.tif]

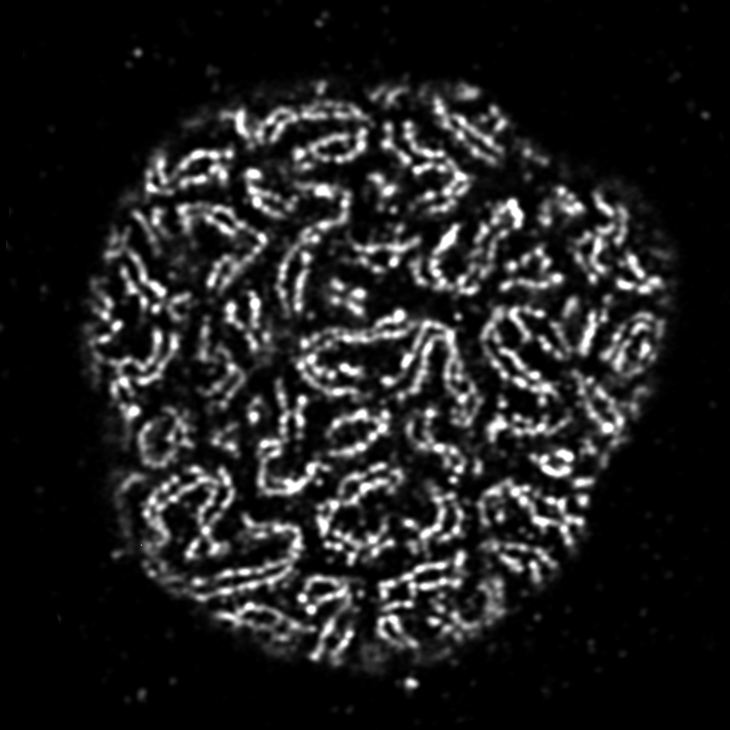

Supplement: Supplementary file 5 — Source Data for Figure 2 [file EMBJ-42-e113475-s004.zip › sd_figure2/panel_e/whole_cell/RGB_220211_5389_2096_c2_rep1_wapl_sor_dep_on_hemi_g2_zoom4_8-02-38.czi #5.tif_registered_slice18_8bit_rotated2_cropped_scc1.tif]

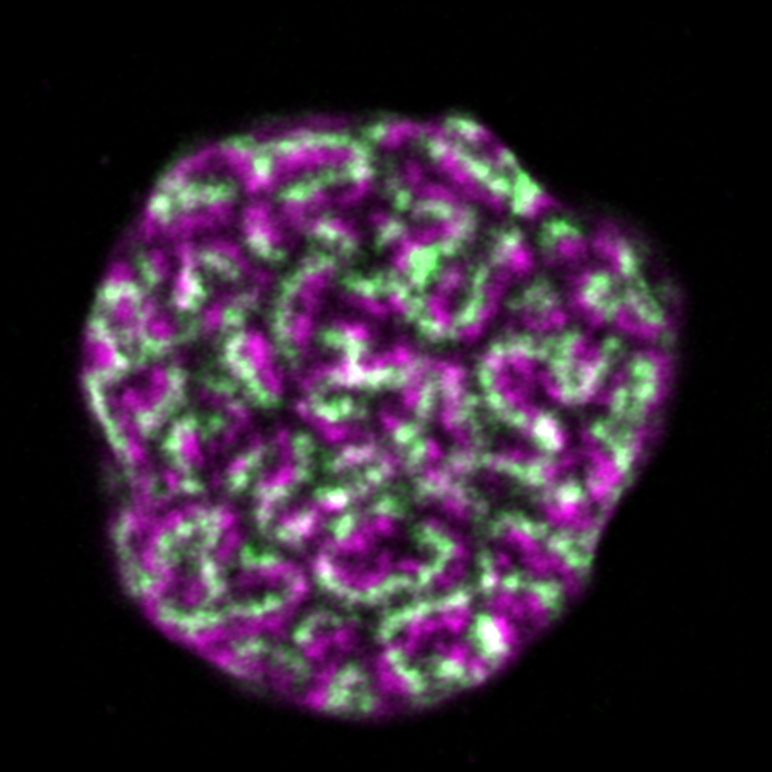

Supplement: Supplementary file 5 — Source Data for Figure 2 [file EMBJ-42-e113475-s004.zip › sd_figure2/panel_e/whole_cell/RGB_220211_5389_2096_c2_rep1_wapl_sor_dep_on_hemi_g2_zoom4_8-02-38.czi #5.tif_registered_slice18_8bit_rotated2_hoechst_edu.tif (RGB).tif]

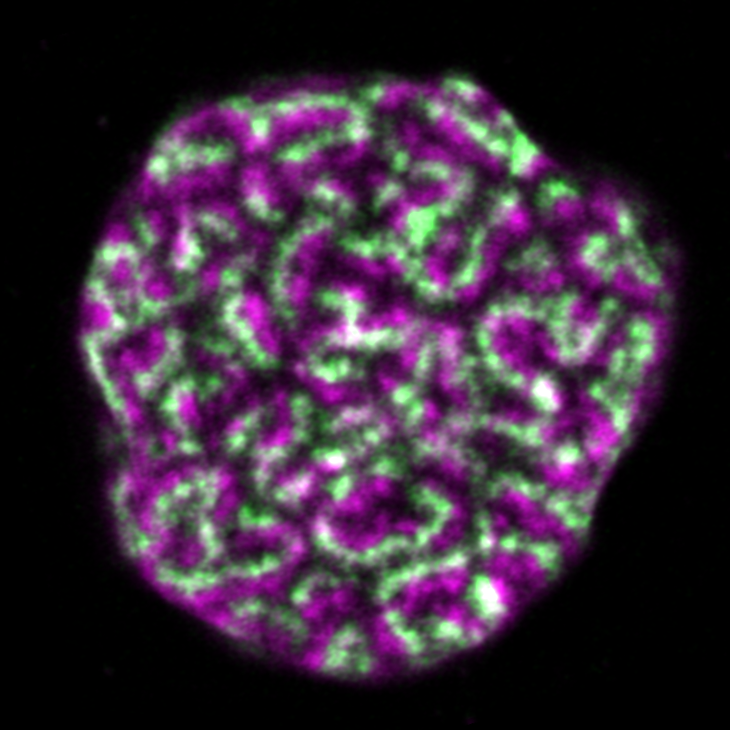

Supplement: Supplementary file 5 — Source Data for Figure 2 [file EMBJ-42-e113475-s004.zip › sd_figure2/panel_e/whole_cell/RGB_220211_5389_2096_c2_rep1_wapl_sor_dep_on_hemi_g2_zoom4_8-02-38.czi #5.tif_registered_slice18_8bit_rotated2_hoechst_edu_cropped.tif]

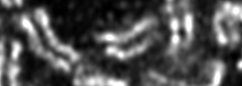

Supplement: Supplementary file 5 — Source Data for Figure 2 [file EMBJ-42-e113475-s004.zip › sd_figure2/panel_f/insets/8bit_220207_5389_WT_c1_rep1_prophase_smc4_axes_60min_stlc_hemi_zoom5-01-46.czi #3.tif_registered_slice18_600x600_8bit_roi2_242x86_all.tif]

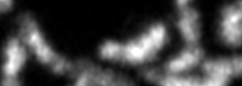

Supplement: Supplementary file 5 — Source Data for Figure 2 [file EMBJ-42-e113475-s004.zip › sd_figure2/panel_f/insets/8bit_220207_5389_WT_c1_rep1_prophase_smc4_axes_60min_stlc_hemi_zoom5-01-46.czi #3.tif_registered_slice18_600x600_8bit_roi2_242x86_hoechst_edu.tif]

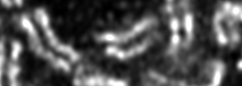

Supplement: Supplementary file 5 — Source Data for Figure 2 [file EMBJ-42-e113475-s004.zip › sd_figure2/panel_f/insets/8bit_220207_5389_WT_c1_rep1_prophase_smc4_axes_60min_stlc_hemi_zoom5-01-46.czi #3.tif_registered_slice18_600x600_8bit_roi2_242x86_smc4.tif]

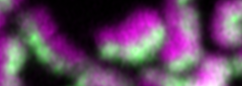

Supplement: Supplementary file 5 — Source Data for Figure 2 [file EMBJ-42-e113475-s004.zip › sd_figure2/panel_f/insets/RGB_220207_5389_WT_c1_rep1_prophase_smc4_axes_60min_stlc_hemi_zoom5-01-46.czi #3.tif_registered_slice18_600x600_8bit_roi2_242x86_hoechst_edu.tif (RGB).tif]

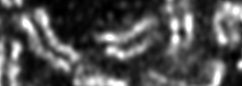

Supplement: Supplementary file 5 — Source Data for Figure 2 [file EMBJ-42-e113475-s004.zip › sd_figure2/panel_f/insets/RGB_220207_5389_WT_c1_rep1_prophase_smc4_axes_60min_stlc_hemi_zoom5-01-46.czi #3.tif_registered_slice18_600x600_8bit_roi2_242x86_smc4_Rgb.tif]

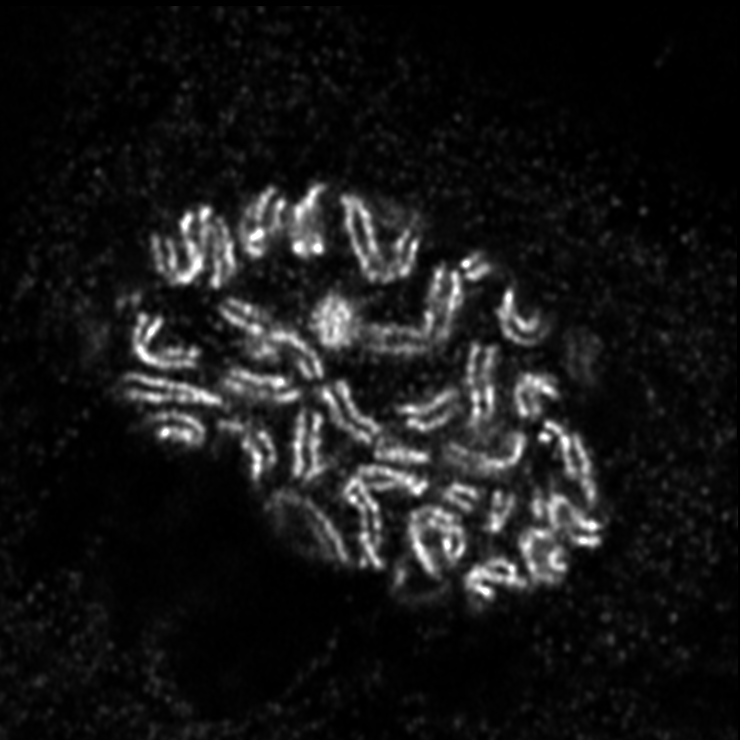

Supplement: Supplementary file 5 — Source Data for Figure 2 [file EMBJ-42-e113475-s004.zip › sd_figure2/panel_f/whole_cell/16bit_220207_5389_WT_c1_rep1_prophase_smc4_axes_60min_stlc_hemi_zoom5-01-46.czi #3.tif_registered_slice18_all.tif]

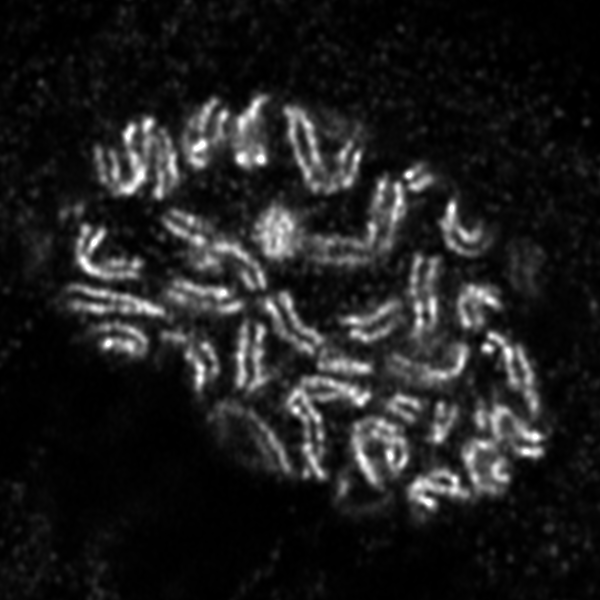

Supplement: Supplementary file 5 — Source Data for Figure 2 [file EMBJ-42-e113475-s004.zip › sd_figure2/panel_f/whole_cell/16bit_220207_5389_WT_c1_rep1_prophase_smc4_axes_60min_stlc_hemi_zoom5-01-46.czi #3.tif_registered_slice18_cropped_all.tif]

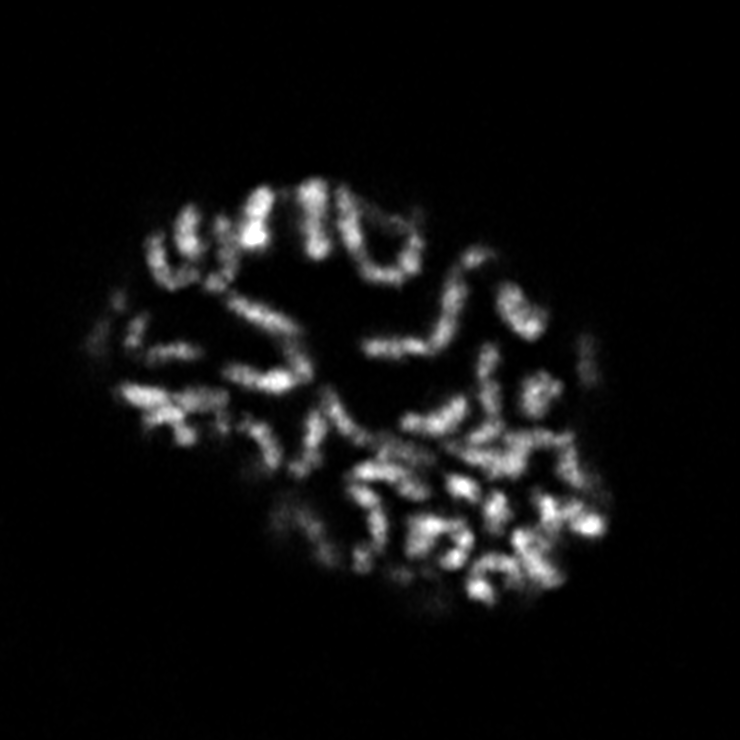

Supplement: Supplementary file 5 — Source Data for Figure 2 [file EMBJ-42-e113475-s004.zip › sd_figure2/panel_f/whole_cell/16bit_220207_5389_WT_c1_rep1_prophase_smc4_axes_60min_stlc_hemi_zoom5-01-46.czi #3.tif_registered_slice18_hoechst_edu.tif]

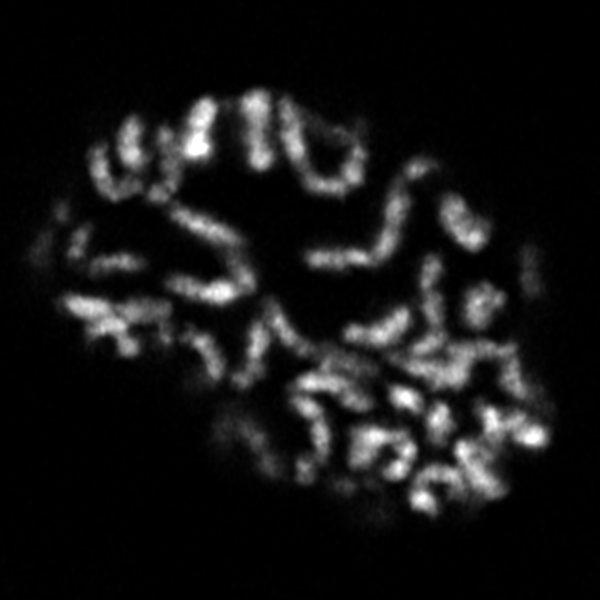

Supplement: Supplementary file 5 — Source Data for Figure 2 [file EMBJ-42-e113475-s004.zip › sd_figure2/panel_f/whole_cell/220207_5389_WT_c1_rep1_prophase_smc4_axes_60min_stlc_hemi_zoom5-01-46.czi #3.tif_registered_slice18_600x600_hoechst_edu.tif]

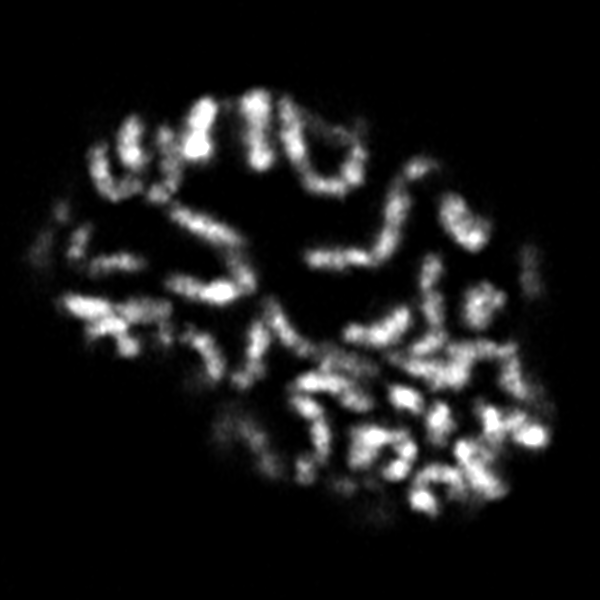

Supplement: Supplementary file 5 — Source Data for Figure 2 [file EMBJ-42-e113475-s004.zip › sd_figure2/panel_f/whole_cell/8bit_220207_5389_WT_c1_rep1_prophase_smc4_axes_60min_stlc_hemi_zoom5-01-46.czi #3.tif_registered_slice18_cropped_hoechst_edu.tif]

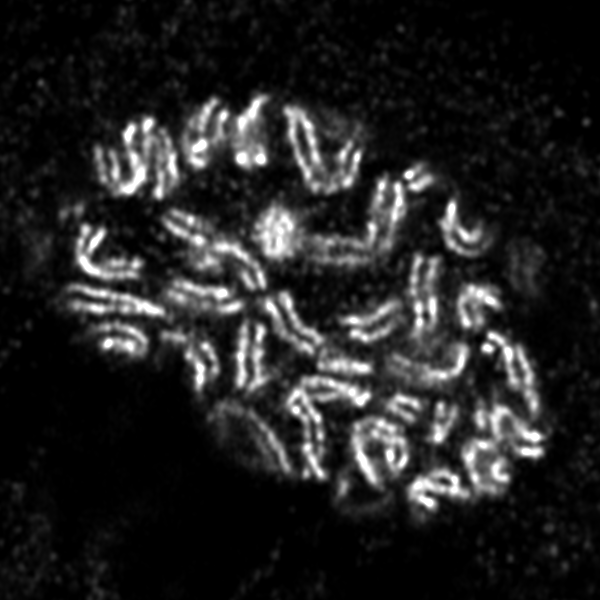

Supplement: Supplementary file 5 — Source Data for Figure 2 [file EMBJ-42-e113475-s004.zip › sd_figure2/panel_f/whole_cell/8bit_220207_5389_WT_c1_rep1_prophase_smc4_axes_60min_stlc_hemi_zoom5-01-46.czi #3.tif_registered_slice18_cropped_smc4.tif]

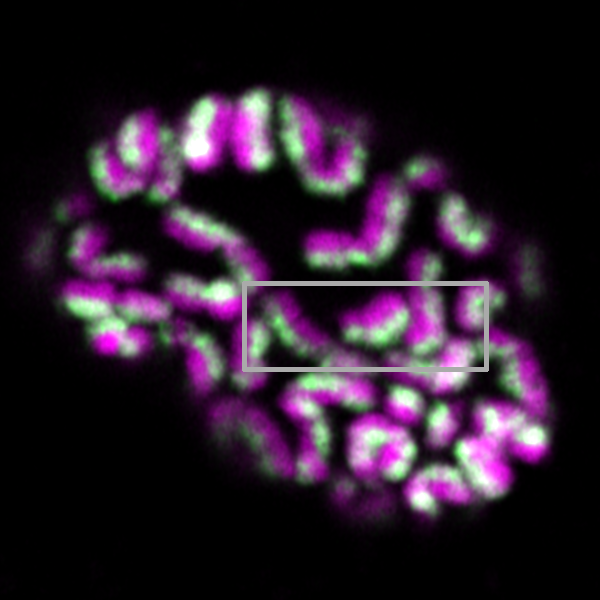

Supplement: Supplementary file 5 — Source Data for Figure 2 [file EMBJ-42-e113475-s004.zip › sd_figure2/panel_f/whole_cell/RGB_220207_5389_WT_c1_rep1_prophase_smc4_axes_60min_stlc_hemi_zoom5-01-46.czi #3.tif_registered_slice18_cropped_hoechst_edu_draw_roi.tif]

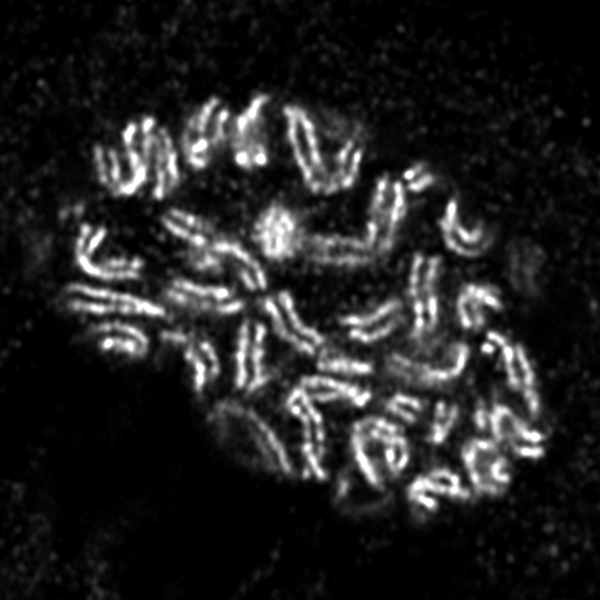

Supplement: Supplementary file 5 — Source Data for Figure 2 [file EMBJ-42-e113475-s004.zip › sd_figure2/panel_f/whole_cell/RGB_220207_5389_WT_c1_rep1_prophase_smc4_axes_60min_stlc_hemi_zoom5-01-46.czi #3.tif_registered_slice18_cropped_smc4.tif]

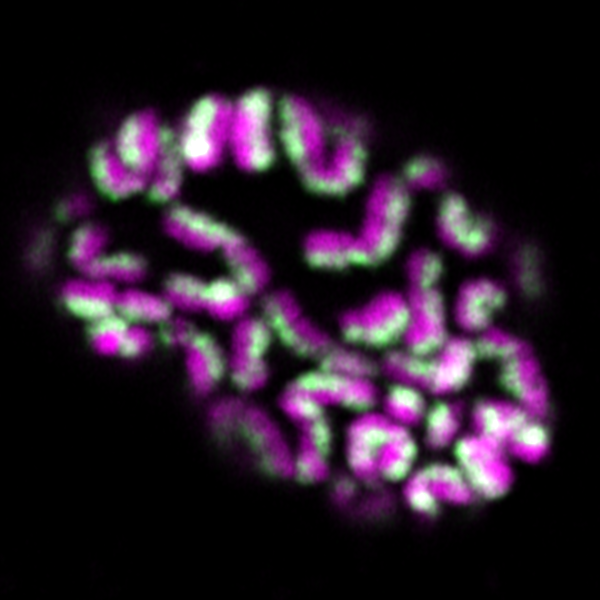

Supplement: Supplementary file 5 — Source Data for Figure 2 [file EMBJ-42-e113475-s004.zip › sd_figure2/panel_f/whole_cell/RGB_220207_5389_WT_c1_rep1_prophase_smc4_axes_60min_stlc_hemi_zoom5-01-46.czi #3.tif_registered_slice18_hoechst_edu.tif.tif]

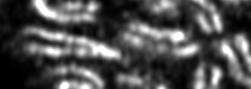

Supplement: Supplementary file 6 — Source Data for Figure 3 [file EMBJ-42-e113475-s005.zip › sd_figure3/panel_a/8bit_220207_5389_WT_c1_rep1_prophase_smc4_axes_60min_stlc_hemi_zoom5-01-46.czi #3.tif_registered_slice18_251x89_all.tif]

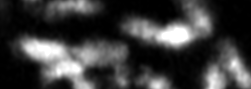

Supplement: Supplementary file 6 — Source Data for Figure 3 [file EMBJ-42-e113475-s005.zip › sd_figure3/panel_a/8bit_220207_5389_WT_c1_rep1_prophase_smc4_axes_60min_stlc_hemi_zoom5-01-46.czi #3.tif_registered_slice18_600x600_8bit_251x89_inset_hoechst_edu.tif]

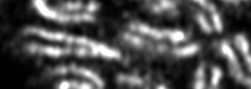

Supplement: Supplementary file 6 — Source Data for Figure 3 [file EMBJ-42-e113475-s005.zip › sd_figure3/panel_a/8bit_220207_5389_WT_c1_rep1_prophase_smc4_axes_60min_stlc_hemi_zoom5-01-46.czi #3.tif_registered_slice18_600x600_8bit_251x89_inset_smc4.tif]

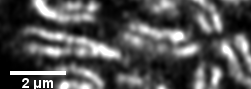

Supplement: Supplementary file 6 — Source Data for Figure 3 [file EMBJ-42-e113475-s005.zip › sd_figure3/panel_a/8bit_220207_5389_WT_c1_rep1_prophase_smc4_axes_60min_stlc_hemi_zoom5-01-46.czi #3.tif_registered_slice18_600x600_8bit_251x89_inset_smc4_sb2um.tif]

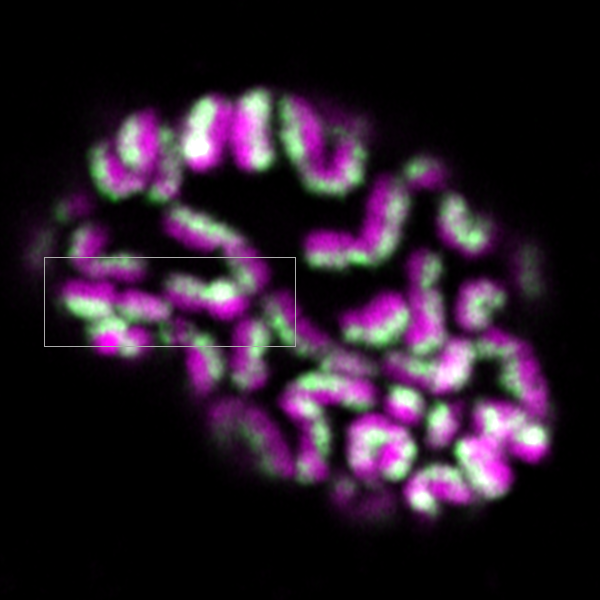

Supplement: Supplementary file 6 — Source Data for Figure 3 [file EMBJ-42-e113475-s005.zip › sd_figure3/panel_a/RGB_220207_5389_WT_c1_rep1_prophase_smc4_axes_60min_stlc_hemi_zoom5-01-46.czi #3.tif_registered_slice18_600x600_8bit.tif draw_roi_251x89.tif]

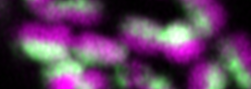

Supplement: Supplementary file 6 — Source Data for Figure 3 [file EMBJ-42-e113475-s005.zip › sd_figure3/panel_a/RGB_220207_5389_WT_c1_rep1_prophase_smc4_axes_60min_stlc_hemi_zoom5-01-46.czi #3.tif_registered_slice18_600x600_8bit_251x89_inset_hoechst_edu.tif.tif]

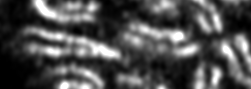

Supplement: Supplementary file 6 — Source Data for Figure 3 [file EMBJ-42-e113475-s005.zip › sd_figure3/panel_a/RGB_220207_5389_WT_c1_rep1_prophase_smc4_axes_60min_stlc_hemi_zoom5-01-46.czi #3.tif_registered_slice18_600x600_8bit_251x89_inset_smc4.tif]

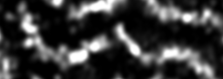

Supplement: Supplementary file 6 — Source Data for Figure 3 [file EMBJ-42-e113475-s005.zip › sd_figure3/panel_b/8bit_220211_5389_2096_c2_rep2_wapl_dep_on_hemi_g2_zoom4_8-05-74.czi #1.tif_registered_slice22_8bit_rotated_223x79_scc1.tif]

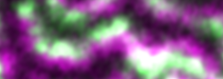

Supplement: Supplementary file 6 — Source Data for Figure 3 [file EMBJ-42-e113475-s005.zip › sd_figure3/panel_b/RGB_220211_5389_2096_c2_rep2_wapl_dep_on_hemi_g2_zoom4_8-05-74.czi #1.tif_registered_slice22_8bit_rotated_223x79_hoechst_edu.tif.tif]

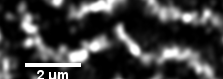

Supplement: Supplementary file 6 — Source Data for Figure 3 [file EMBJ-42-e113475-s005.zip › sd_figure3/panel_b/RGB_220211_5389_2096_c2_rep2_wapl_dep_on_hemi_g2_zoom4_8-05-74.czi #1.tif_registered_slice22_8bit_rotated_223x79_sb2.tif]

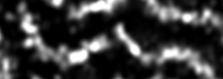

Supplement: Supplementary file 6 — Source Data for Figure 3 [file EMBJ-42-e113475-s005.zip › sd_figure3/panel_b/RGB_220211_5389_2096_c2_rep2_wapl_dep_on_hemi_g2_zoom4_8-05-74.czi #1.tif_registered_slice22_8bit_rotated_223x79_scc1.tif]

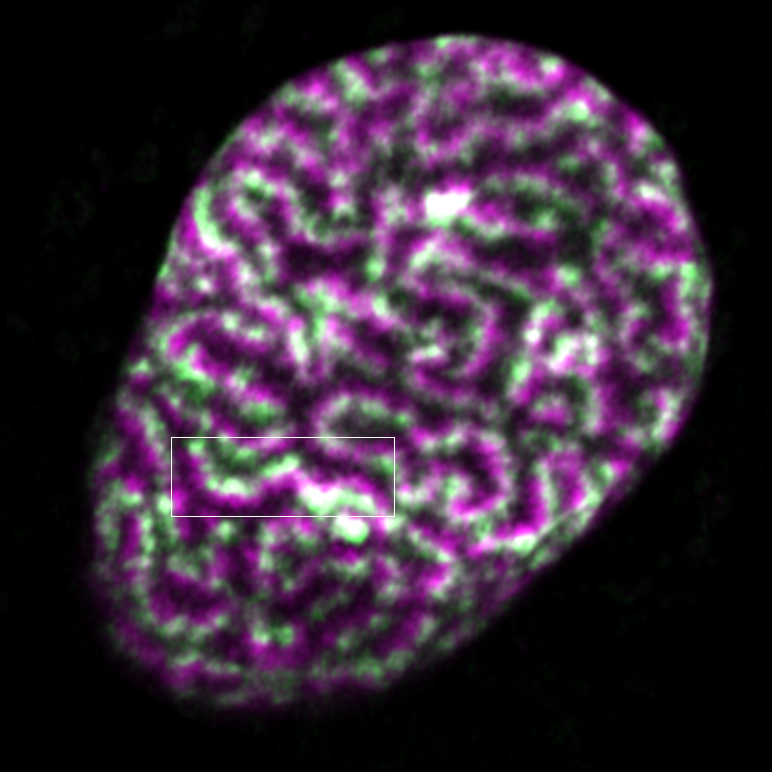

Supplement: Supplementary file 6 — Source Data for Figure 3 [file EMBJ-42-e113475-s005.zip › sd_figure3/panel_b/RGB_220211_5389_2096_c2_rep2_wapl_dep_on_hemi_g2_zoom4_8-05-74.czi #1.tif_registered_slice22_8bit_rotated-1.tif_draw_roi_223x79.tif]

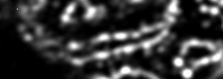

Supplement: Supplementary file 6 — Source Data for Figure 3 [file EMBJ-42-e113475-s005.zip › sd_figure3/panel_c/8bit_220211_5389_2096_c2_rep1_wapl_sor_dep_on_hemi_g2_zoom4_8-02-38.czi #5.tif_registered_slice18_8bit_rotated2_730x730_scc1.tif]

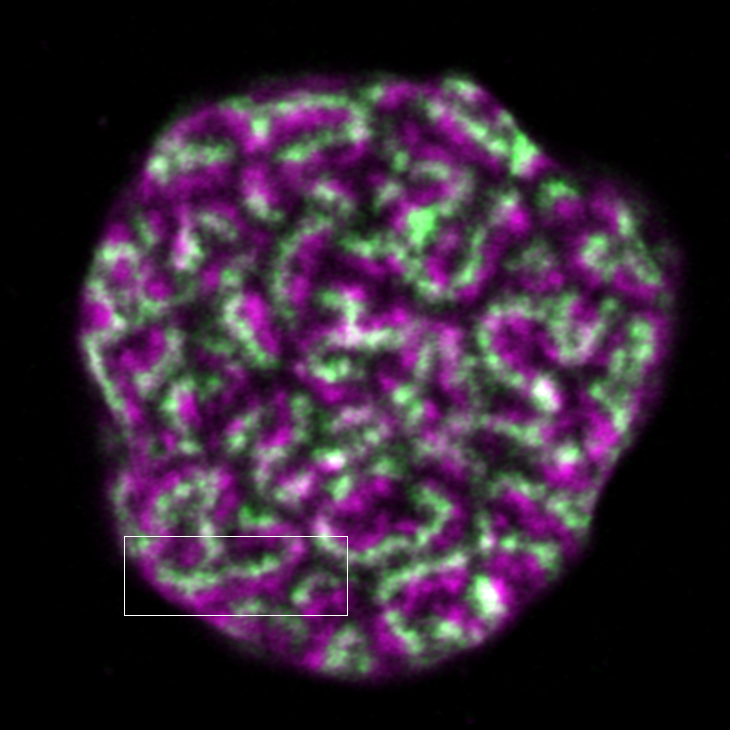

Supplement: Supplementary file 6 — Source Data for Figure 3 [file EMBJ-42-e113475-s005.zip › sd_figure3/panel_c/RGB_220211_5389_2096_c2_rep1_wapl_sor_dep_on_hemi_g2_zoom4_8-02-38.czi #5.tif_registered_slice18_8bit_rotated2_730x730.tif_draw_roi.tif]

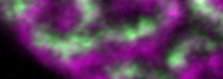

Supplement: Supplementary file 6 — Source Data for Figure 3 [file EMBJ-42-e113475-s005.zip › sd_figure3/panel_c/RGB_220211_5389_2096_c2_rep1_wapl_sor_dep_on_hemi_g2_zoom4_8-02-38.czi #5.tif_registered_slice18_8bit_rotated2_730x730-_hoechst_Edu.tif (RGB).tif]

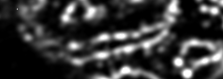

Supplement: Supplementary file 6 — Source Data for Figure 3 [file EMBJ-42-e113475-s005.zip › sd_figure3/panel_c/RGB_220211_5389_2096_c2_rep1_wapl_sor_dep_on_hemi_g2_zoom4_8-02-38.czi #5.tif_registered_slice18_8bit_rotated2_730x730_scc1.tif]

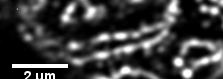

Supplement: Supplementary file 6 — Source Data for Figure 3 [file EMBJ-42-e113475-s005.zip › sd_figure3/panel_c/RGB_220211_5389_2096_c2_rep1_wapl_sor_dep_on_hemi_g2_zoom4_8-02-38.czi #5.tif_registered_slice18_8bit_rotated2_730x730_scc1_sb.tif]

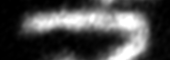

Supplement: Supplementary file 7 — Source Data for Figure 4 [file EMBJ-42-e113475-s001.zip › sd_figure4/panel_a/wapl_dep_prometa/insets/8bit_5303_2108_wapl_depleted_ON_G2_hemi_prometa_zoom5_rep2stain_smc4_cycb1-06-25.tif_registered_slice45_650x650_edu_gray.tif]

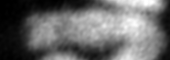

Supplement: Supplementary file 7 — Source Data for Figure 4 [file EMBJ-42-e113475-s001.zip › sd_figure4/panel_a/wapl_dep_prometa/insets/8bit_5303_2108_wapl_depleted_ON_G2_hemi_prometa_zoom5_rep2stain_smc4_cycb1-06-25.tif_registered_slice45_650x650_hoechst_gray.tif]

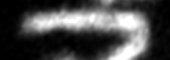

Supplement: Supplementary file 7 — Source Data for Figure 4 [file EMBJ-42-e113475-s001.zip › sd_figure4/panel_a/wapl_dep_prometa/insets/RGB_5303_2108_wapl_depleted_ON_G2_hemi_prometa_zoom5_rep2stain_smc4_cycb1-06-25.tif_registered_slice45_650x650_edu_gray.tif]

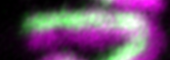

Supplement: Supplementary file 7 — Source Data for Figure 4 [file EMBJ-42-e113475-s001.zip › sd_figure4/panel_a/wapl_dep_prometa/insets/RGB_5303_2108_wapl_depleted_ON_G2_hemi_prometa_zoom5_rep2stain_smc4_cycb1-06-25.tif_registered_slice45_650x650_hoechst_edu_rotated_inset.tif.tif]

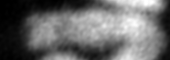

Supplement: Supplementary file 7 — Source Data for Figure 4 [file EMBJ-42-e113475-s001.zip › sd_figure4/panel_a/wapl_dep_prometa/insets/RGB_5303_2108_wapl_depleted_ON_G2_hemi_prometa_zoom5_rep2stain_smc4_cycb1-06-25.tif_registered_slice45_650x650_hoechst_gray.tif]

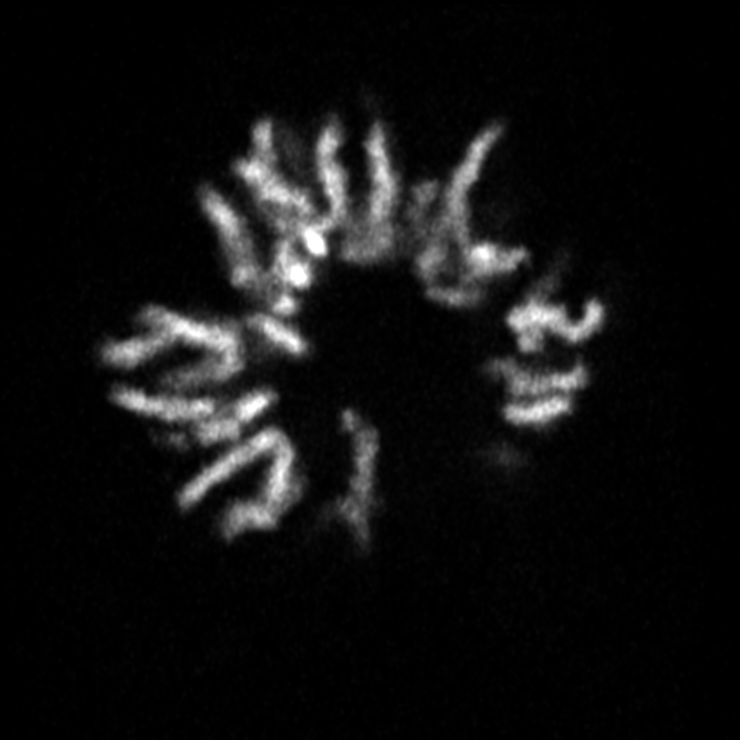

Supplement: Supplementary file 7 — Source Data for Figure 4 [file EMBJ-42-e113475-s001.zip › sd_figure4/panel_a/wapl_dep_prometa/whole_cell/16bit_5303_2108_wapl_depleted_ON_G2_hemi_prometa_zoom5_rep2stain_smc4_cycb1-06-25.tif_registered_slice45_hoechst_edu.tif]

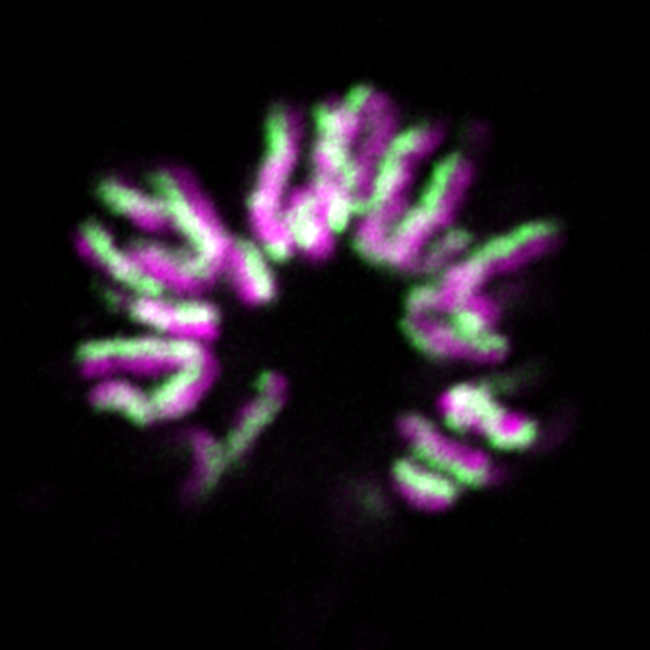

Supplement: Supplementary file 7 — Source Data for Figure 4 [file EMBJ-42-e113475-s001.zip › sd_figure4/panel_a/wapl_dep_prometa/whole_cell/RGB_5303_2108_wapl_depleted_ON_G2_hemi_prometa_zoom5_rep2stain_smc4_cycb1-06-25.tif_registered_slice45_650x650_hoechst_edu_rotated.tif (RGB).tif]

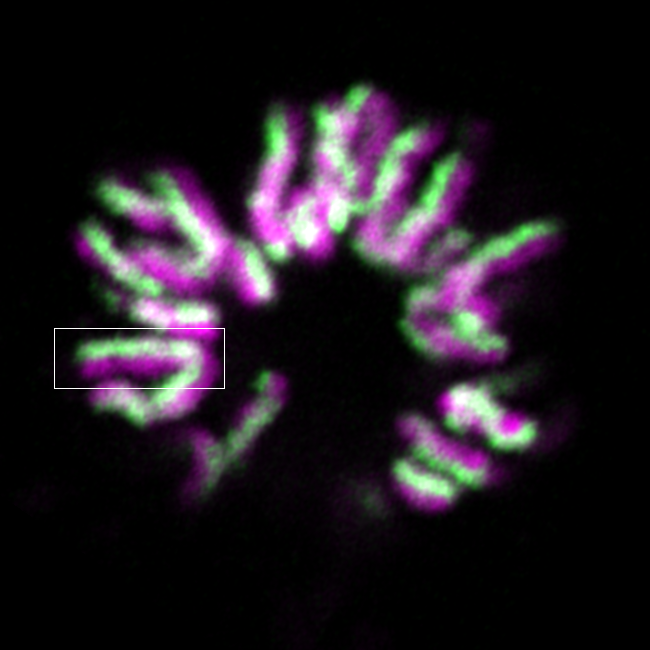

Supplement: Supplementary file 7 — Source Data for Figure 4 [file EMBJ-42-e113475-s001.zip › sd_figure4/panel_a/wapl_dep_prometa/whole_cell/RGB_5303_2108_wapl_depleted_ON_G2_hemi_prometa_zoom5_rep2stain_smc4_cycb1-06-25.tif_registered_slice45_650x650_hoechst_edu_rotated.tif_draw_roi.tif]

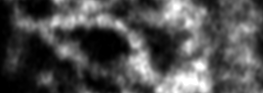

Supplement: Supplementary file 7 — Source Data for Figure 4 [file EMBJ-42-e113475-s001.zip › sd_figure4/panel_a/wapl_smc4_dep_prometa/insets/8bit_5303_2108_ON_G2_ctrl_siRNA_wapl_smc4_depleted_prometa_hemi_zoom4.5_rep1_stain_scc1_cycb1-06-19.czi #2.tif_registered_slice53_edu.tif]

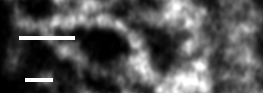

Supplement: Supplementary file 7 — Source Data for Figure 4 [file EMBJ-42-e113475-s001.zip › sd_figure4/panel_a/wapl_smc4_dep_prometa/insets/8bit_5303_2108_ON_G2_ctrl_siRNA_wapl_smc4_depleted_prometa_hemi_zoom4.5_rep1_stain_scc1_cycb1-06-19.czi #2.tif_registered_slice53_edu_sb.tif]

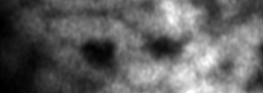

Supplement: Supplementary file 7 — Source Data for Figure 4 [file EMBJ-42-e113475-s001.zip › sd_figure4/panel_a/wapl_smc4_dep_prometa/insets/8bit_5303_2108_ON_G2_ctrl_siRNA_wapl_smc4_depleted_prometa_hemi_zoom4.5_rep1_stain_scc1_cycb1-06-19.czi #2.tif_registered_slice53_hoechst.tif]

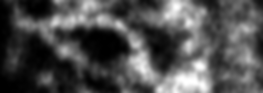

Supplement: Supplementary file 7 — Source Data for Figure 4 [file EMBJ-42-e113475-s001.zip › sd_figure4/panel_a/wapl_smc4_dep_prometa/insets/RGB_5303_2108_ON_G2_ctrl_siRNA_wapl_smc4_depleted_prometa_hemi_zoom4.5_rep1_stain_scc1_cycb1-06-19.czi #2.tif_registered_slice53_edu.tif]

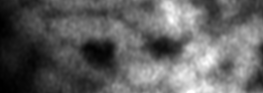

Supplement: Supplementary file 7 — Source Data for Figure 4 [file EMBJ-42-e113475-s001.zip › sd_figure4/panel_a/wapl_smc4_dep_prometa/insets/RGB_5303_2108_ON_G2_ctrl_siRNA_wapl_smc4_depleted_prometa_hemi_zoom4.5_rep1_stain_scc1_cycb1-06-19.czi #2.tif_registered_slice53_hoechst.tif]

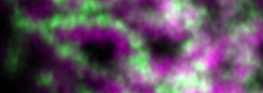

Supplement: Supplementary file 7 — Source Data for Figure 4 [file EMBJ-42-e113475-s001.zip › sd_figure4/panel_a/wapl_smc4_dep_prometa/insets/RGB_5303_2108_ON_G2_ctrl_siRNA_wapl_smc4_depleted_prometa_hemi_zoom4.5_rep1_stain_scc1_cycb1-06-19.czi #2.tif_registered_slice53_hoechst_edu.tif.tif]

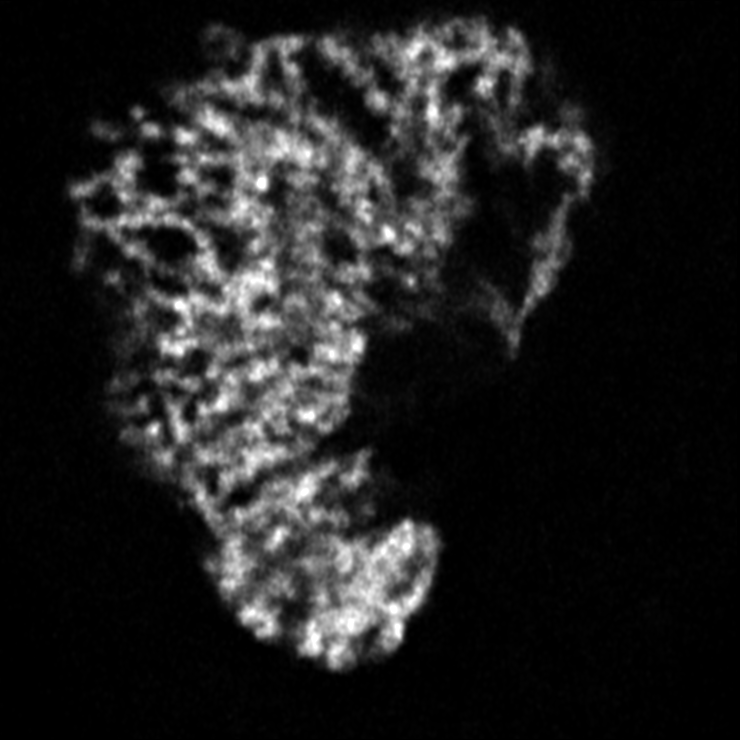

Supplement: Supplementary file 7 — Source Data for Figure 4 [file EMBJ-42-e113475-s001.zip › sd_figure4/panel_a/wapl_smc4_dep_prometa/whole_cell/16bit_5303_2108_ON_G2_ctrl_siRNA_wapl_smc4_depleted_prometa_hemi_zoom4.5_rep1_stain_scc1_cycb1-06-19.czi #2.tif_registered_slice53_hoechst_edu.tif]

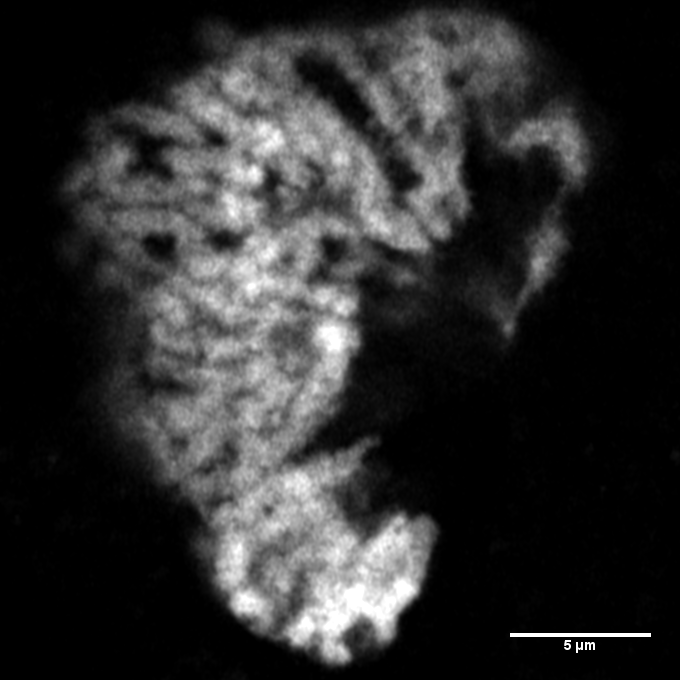

Supplement: Supplementary file 7 — Source Data for Figure 4 [file EMBJ-42-e113475-s001.zip › sd_figure4/panel_a/wapl_smc4_dep_prometa/whole_cell/5303_2108_ON_G2_ctrl_siRNA_wapl_smc4_depleted_prometa_hemi_zoom4.5_rep1_stain_scc1_cycb1-06-19.czi #2.tif_registered_slice53_cropped_hoechst_gray_sb.tif]

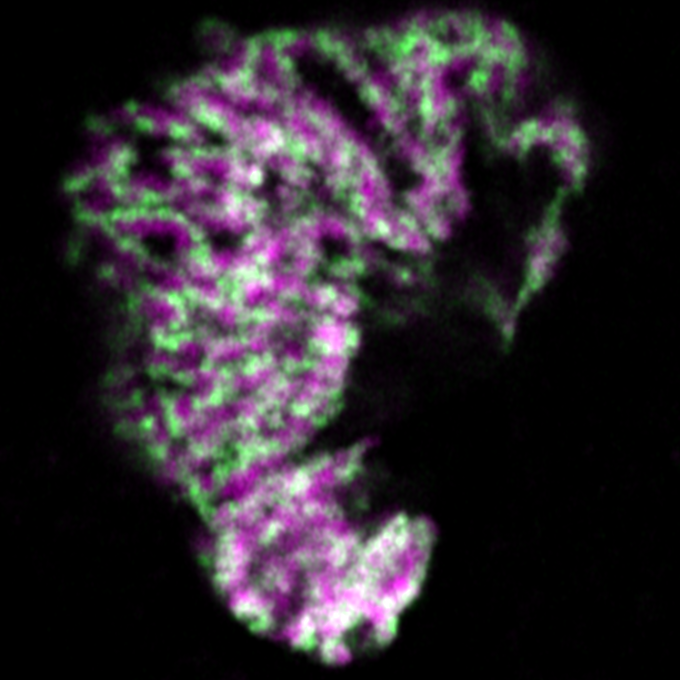

Supplement: Supplementary file 7 — Source Data for Figure 4 [file EMBJ-42-e113475-s001.zip › sd_figure4/panel_a/wapl_smc4_dep_prometa/whole_cell/RGB_5303_2108_ON_G2_ctrl_siRNA_wapl_smc4_depleted_prometa_hemi_zoom4.5_rep1_stain_scc1_cycb1-06-19.czi #2.tif_registered_slice53_crop_hoechst_edu.tif (RGB).tif]

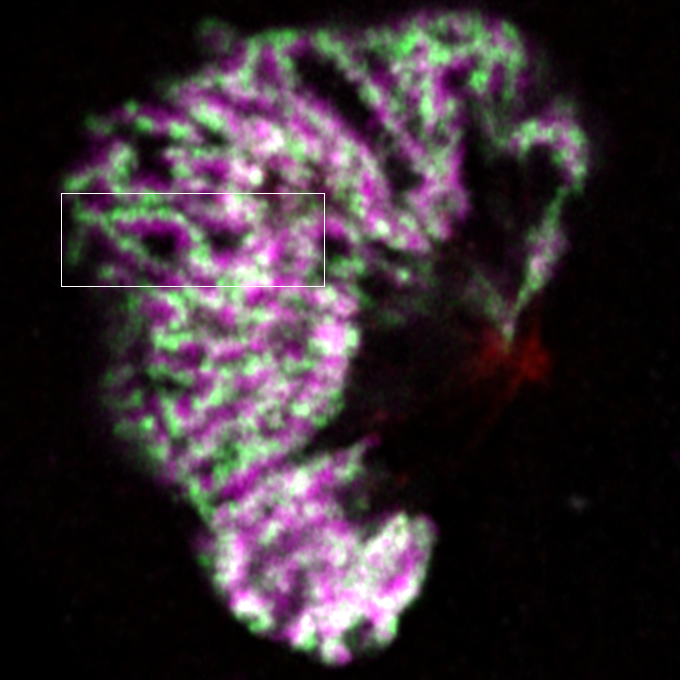

Supplement: Supplementary file 7 — Source Data for Figure 4 [file EMBJ-42-e113475-s001.zip › sd_figure4/panel_a/wapl_smc4_dep_prometa/whole_cell/RGB_5303_2108_ON_G2_ctrl_siRNA_wapl_smc4_depleted_prometa_hemi_zoom4.5_rep1_stain_scc1_cycb1-06-19.czi #2.tif_registered_slice53_cropped.tif draw_roi.tif]

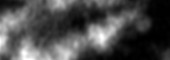

Supplement: Supplementary file 7 — Source Data for Figure 4 [file EMBJ-42-e113475-s001.zip › sd_figure4/panel_a/wapl_sor_smc4_dep_prometa/insets/16bit_5303_2108_wapl_sor_smc4_depleted_hemi_prometa_zoom5_rep2_stain_smc4_cycb1-02-08.czi #6.tif_registered_slice28_edu.tif]

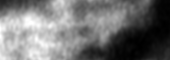

Supplement: Supplementary file 7 — Source Data for Figure 4 [file EMBJ-42-e113475-s001.zip › sd_figure4/panel_a/wapl_sor_smc4_dep_prometa/insets/16bit_5303_2108_wapl_sor_smc4_depleted_hemi_prometa_zoom5_rep2_stain_smc4_cycb1-02-08.czi #6.tif_registered_slice28_hoechst.tif]

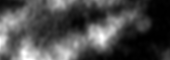

Supplement: Supplementary file 7 — Source Data for Figure 4 [file EMBJ-42-e113475-s001.zip › sd_figure4/panel_a/wapl_sor_smc4_dep_prometa/insets/16bit_5303_2108_wapl_sor_smc4_depleted_hemi_prometa_zoom5_rep2_stain_smc4_cycb1-02-08.czi #6.tif_registered_slice28_hoechst_edu.tif]

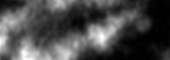

Supplement: Supplementary file 7 — Source Data for Figure 4 [file EMBJ-42-e113475-s001.zip › sd_figure4/panel_a/wapl_sor_smc4_dep_prometa/insets/RGB_5303_2108_wapl_sor_smc4_depleted_hemi_prometa_zoom5_rep2_stain_smc4_cycb1-02-08.czi #6.tif_registered_slice28_edu.tif]

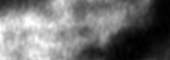

Supplement: Supplementary file 7 — Source Data for Figure 4 [file EMBJ-42-e113475-s001.zip › sd_figure4/panel_a/wapl_sor_smc4_dep_prometa/insets/RGB_5303_2108_wapl_sor_smc4_depleted_hemi_prometa_zoom5_rep2_stain_smc4_cycb1-02-08.czi #6.tif_registered_slice28_hoechst.tif]

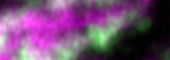

Supplement: Supplementary file 7 — Source Data for Figure 4 [file EMBJ-42-e113475-s001.zip › sd_figure4/panel_a/wapl_sor_smc4_dep_prometa/insets/RGB_5303_2108_wapl_sor_smc4_depleted_hemi_prometa_zoom5_rep2_stain_smc4_cycb1-02-08.czi #6.tif_registered_slice28_hoechst_edu.tif]

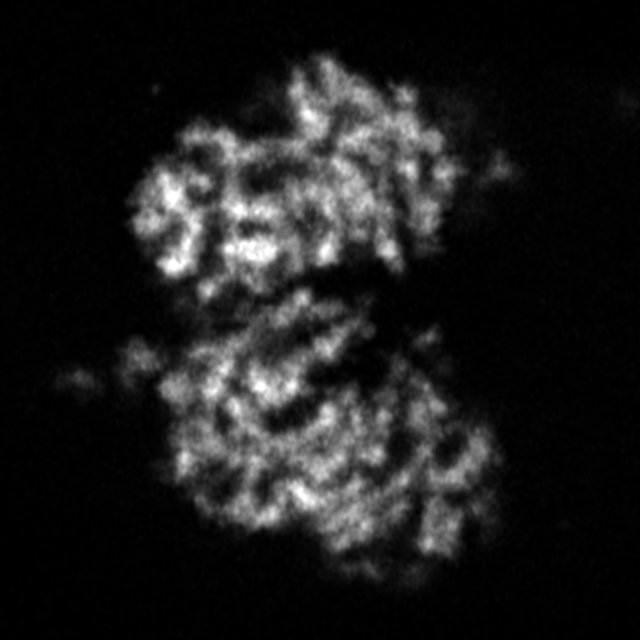

Supplement: Supplementary file 7 — Source Data for Figure 4 [file EMBJ-42-e113475-s001.zip › sd_figure4/panel_a/wapl_sor_smc4_dep_prometa/whole_cell/16bit_5303_2108_wapl_sor_smc4_depleted_hemi_prometa_zoom5_rep2_stain_smc4_cycb1-02-08.czi #6.tif_registered_slice28_crop_hoechst_edu.tif]

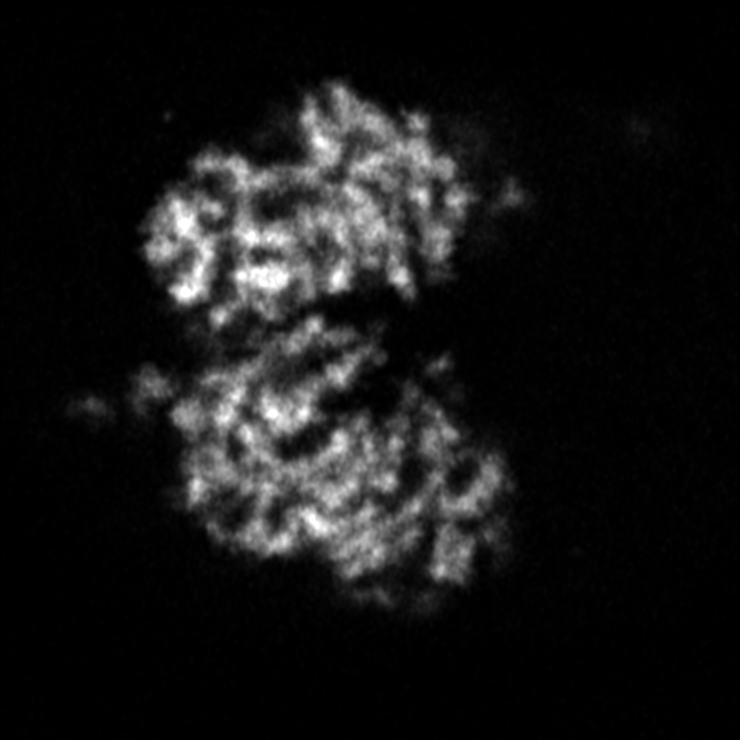

Supplement: Supplementary file 7 — Source Data for Figure 4 [file EMBJ-42-e113475-s001.zip › sd_figure4/panel_a/wapl_sor_smc4_dep_prometa/whole_cell/16bit_5303_2108_wapl_sor_smc4_depleted_hemi_prometa_zoom5_rep2_stain_smc4_cycb1-02-08.czi #6.tif_registered_slice28_hoechst_edu.tif]

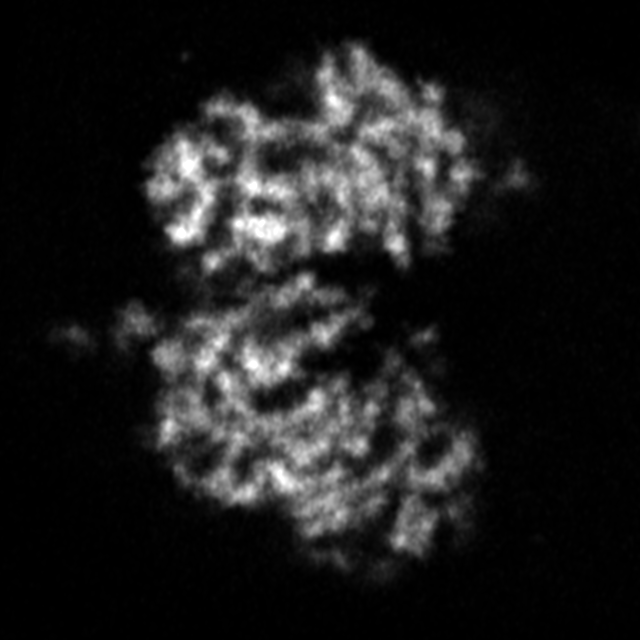

Supplement: Supplementary file 7 — Source Data for Figure 4 [file EMBJ-42-e113475-s001.zip › sd_figure4/panel_a/wapl_sor_smc4_dep_prometa/whole_cell/16bit_5303_2108_wapl_sor_smc4_depleted_hemi_prometa_zoom5_rep2_stain_smc4_cycb1-02-08.czi #6.tif_registered_slice28_rotate_crop_hoechst_edu.tif]

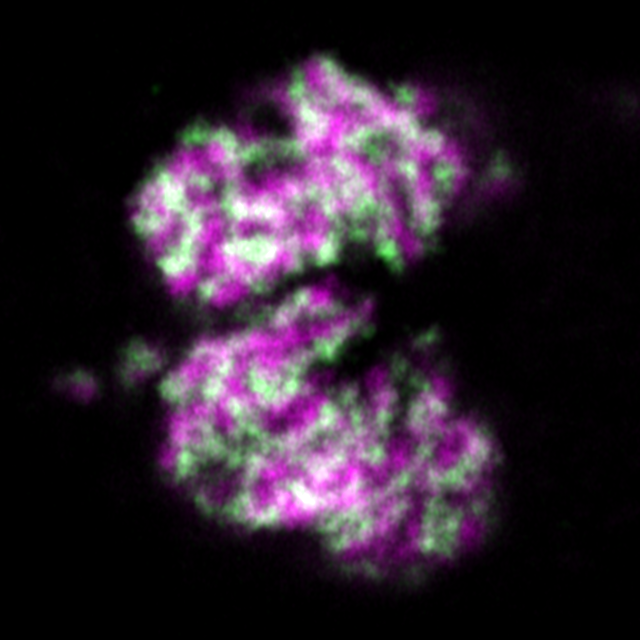

Supplement: Supplementary file 7 — Source Data for Figure 4 [file EMBJ-42-e113475-s001.zip › sd_figure4/panel_a/wapl_sor_smc4_dep_prometa/whole_cell/RGB_5303_2108_wapl_sor_smc4_depleted_hemi_prometa_zoom5_rep2_stain_smc4_cycb1-02-08.czi #6.tif_registered_slice28_crop_hoechst_edu.tif.tif]

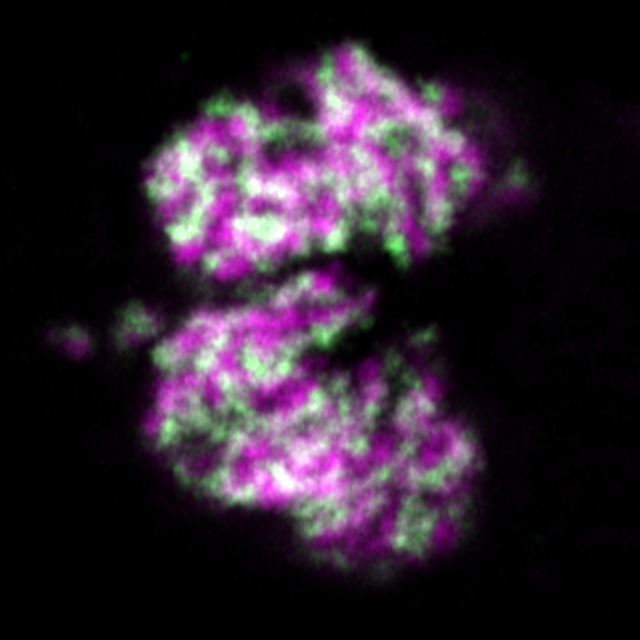

Supplement: Supplementary file 7 — Source Data for Figure 4 [file EMBJ-42-e113475-s001.zip › sd_figure4/panel_a/wapl_sor_smc4_dep_prometa/whole_cell/RGB_5303_2108_wapl_sor_smc4_depleted_hemi_prometa_zoom5_rep2_stain_smc4_cycb1-02-08.czi #6.tif_registered_slice28_rotate_crop_hoechst_edu.tif]

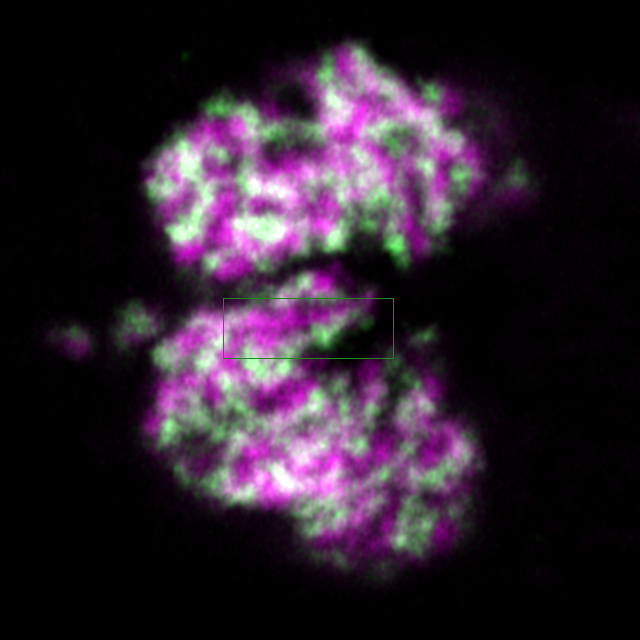

Supplement: Supplementary file 7 — Source Data for Figure 4 [file EMBJ-42-e113475-s001.zip › sd_figure4/panel_a/wapl_sor_smc4_dep_prometa/whole_cell/RGB_5303_2108_wapl_sor_smc4_depleted_hemi_prometa_zoom5_rep2_stain_smc4_cycb1-02-08.czi #6.tif_registered_slice28_rotate_draw_roi.tif]

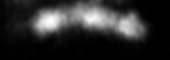

Supplement: Supplementary file 7 — Source Data for Figure 4 [file EMBJ-42-e113475-s001.zip › sd_figure4/panel_c/control/insets/16bit_230316_2108_ctrl_rep1_hemi_stlc_60min_zoom5-06-04.tif_registered_slice59_rotated_600x600_edu_inset_170x60.tif]

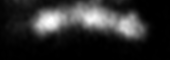

Supplement: Supplementary file 7 — Source Data for Figure 4 [file EMBJ-42-e113475-s001.zip › sd_figure4/panel_c/control/insets/16bit_230316_2108_ctrl_rep1_hemi_stlc_60min_zoom5-06-04.tif_registered_slice59_rotated_600x600_hoechst_edu_inset_170x60.tif]

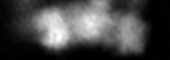

Supplement: Supplementary file 7 — Source Data for Figure 4 [file EMBJ-42-e113475-s001.zip › sd_figure4/panel_c/control/insets/16bit_230316_2108_ctrl_rep1_hemi_stlc_60min_zoom5-06-04.tif_registered_slice59_rotated_600x600_hoechst_inset_170x60.tif]

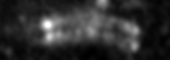

Supplement: Supplementary file 7 — Source Data for Figure 4 [file EMBJ-42-e113475-s001.zip › sd_figure4/panel_c/control/insets/16bit_230316_2108_ctrl_rep1_hemi_stlc_60min_zoom5-06-04.tif_registered_slice59_rotated_600x600_inset_smc4.tif]

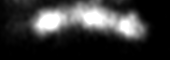

Supplement: Supplementary file 7 — Source Data for Figure 4 [file EMBJ-42-e113475-s001.zip › sd_figure4/panel_c/control/insets/RGB_230316_2108_ctrl_rep1_hemi_stlc_60min_zoom5-06-04.tif_registered_slice59_rotated_600x600_edu_inset_170x60.tif]

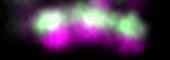

Supplement: Supplementary file 7 — Source Data for Figure 4 [file EMBJ-42-e113475-s001.zip › sd_figure4/panel_c/control/insets/RGB_230316_2108_ctrl_rep1_hemi_stlc_60min_zoom5-06-04.tif_registered_slice59_rotated_600x600_hoechst_edu_inset_170x60.tif.tif]

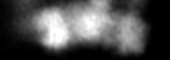

Supplement: Supplementary file 7 — Source Data for Figure 4 [file EMBJ-42-e113475-s001.zip › sd_figure4/panel_c/control/insets/RGB_230316_2108_ctrl_rep1_hemi_stlc_60min_zoom5-06-04.tif_registered_slice59_rotated_600x600_hoechst_inset_170x60.tif]

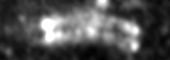

Supplement: Supplementary file 7 — Source Data for Figure 4 [file EMBJ-42-e113475-s001.zip › sd_figure4/panel_c/control/insets/RGB_230316_2108_ctrl_rep1_hemi_stlc_60min_zoom5-06-04.tif_registered_slice59_rotated_600x600_inset_smc4.tif]

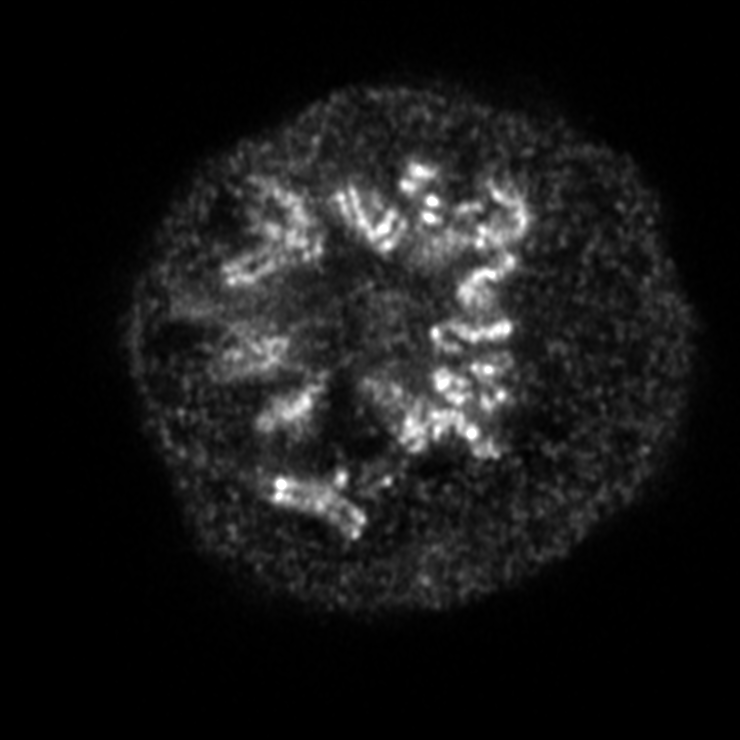

Supplement: Supplementary file 7 — Source Data for Figure 4 [file EMBJ-42-e113475-s001.zip › sd_figure4/panel_c/control/whole_cell/16bit_230316_2108_ctrl_rep1_hemi_stlc_60min_zoom5-06-04.tif_registered_slice59_all.tif]

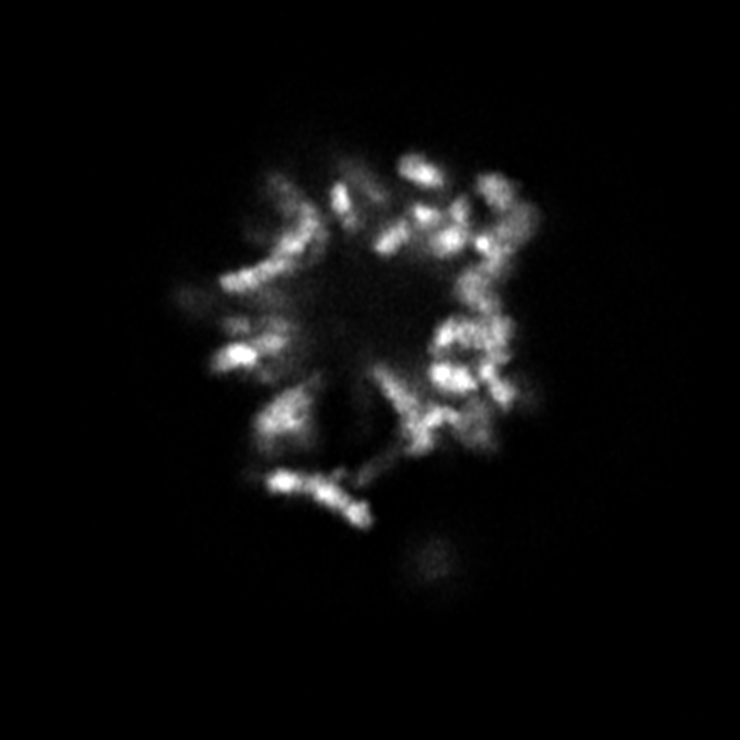

Supplement: Supplementary file 7 — Source Data for Figure 4 [file EMBJ-42-e113475-s001.zip › sd_figure4/panel_c/control/whole_cell/16bit_230316_2108_ctrl_rep1_hemi_stlc_60min_zoom5-06-04.tif_registered_slice59_hoechst_edu.tif]

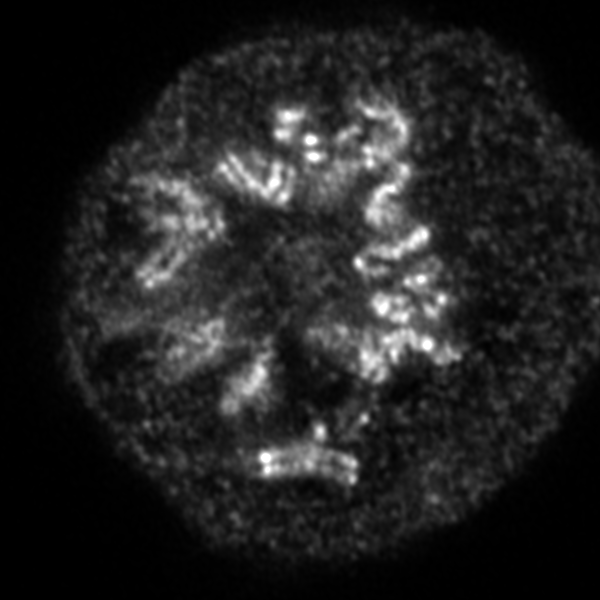

Supplement: Supplementary file 7 — Source Data for Figure 4 [file EMBJ-42-e113475-s001.zip › sd_figure4/panel_c/control/whole_cell/16bit_230316_2108_ctrl_rep1_hemi_stlc_60min_zoom5-06-04.tif_registered_slice59_rotated_600x600_smc4.tif]

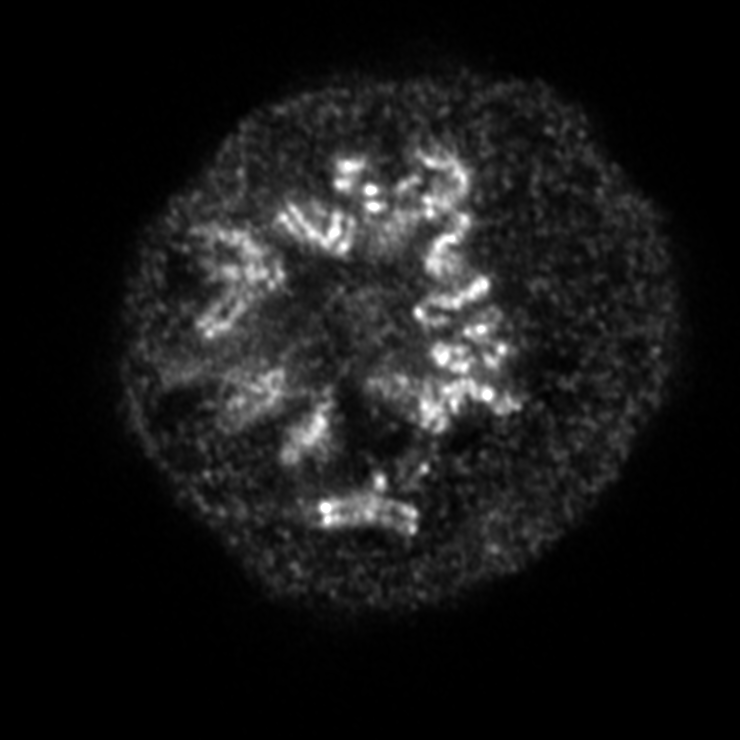

Supplement: Supplementary file 7 — Source Data for Figure 4 [file EMBJ-42-e113475-s001.zip › sd_figure4/panel_c/control/whole_cell/16bit_230316_2108_ctrl_rep1_hemi_stlc_60min_zoom5-06-04.tif_registered_slice59_rotated_all.tif]

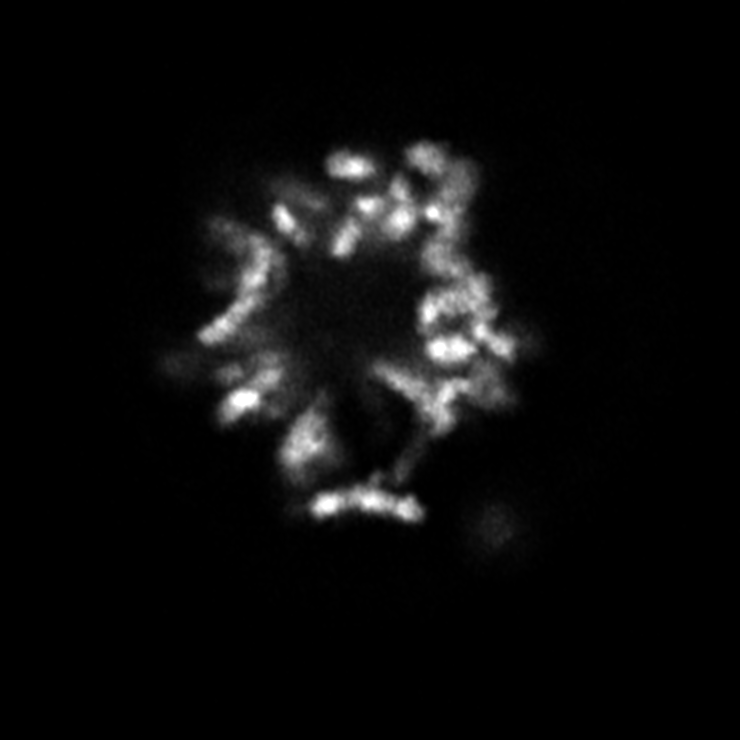

Supplement: Supplementary file 7 — Source Data for Figure 4 [file EMBJ-42-e113475-s001.zip › sd_figure4/panel_c/control/whole_cell/16bit_230316_2108_ctrl_rep1_hemi_stlc_60min_zoom5-06-04.tif_registered_slice59_rotated_hoechst_edu.tif]

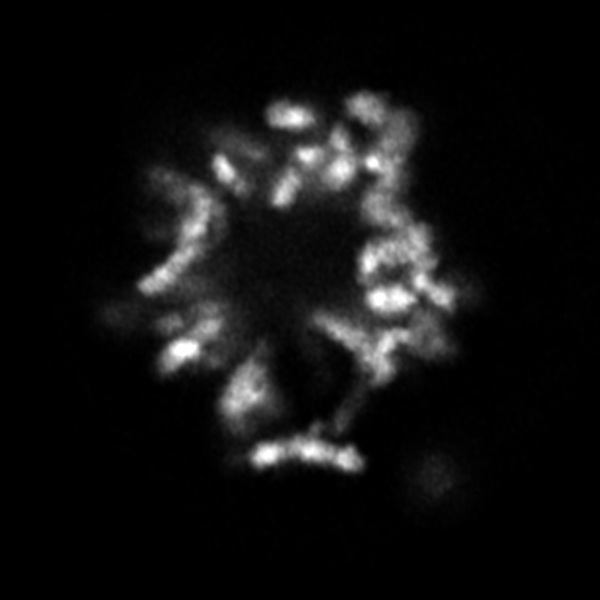

Supplement: Supplementary file 7 — Source Data for Figure 4 [file EMBJ-42-e113475-s001.zip › sd_figure4/panel_c/control/whole_cell/230316_2108_ctrl_rep1_hemi_stlc_60min_zoom5-06-04.tif_registered_slice59_rotated_600x600_hoechst_edu.tif]

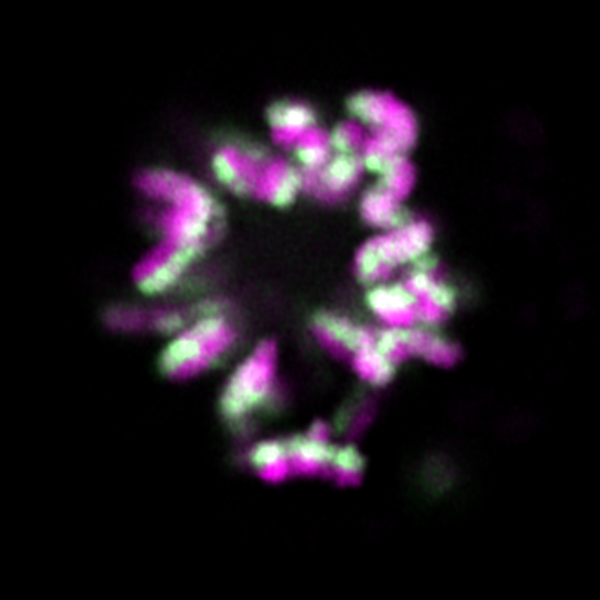

Supplement: Supplementary file 7 — Source Data for Figure 4 [file EMBJ-42-e113475-s001.zip › sd_figure4/panel_c/control/whole_cell/RGB_230316_2108_ctrl_rep1_hemi_stlc_60min_zoom5-06-04.tif_registered_slice59_rotated_600x600_hoechst_edu.tif.tif]

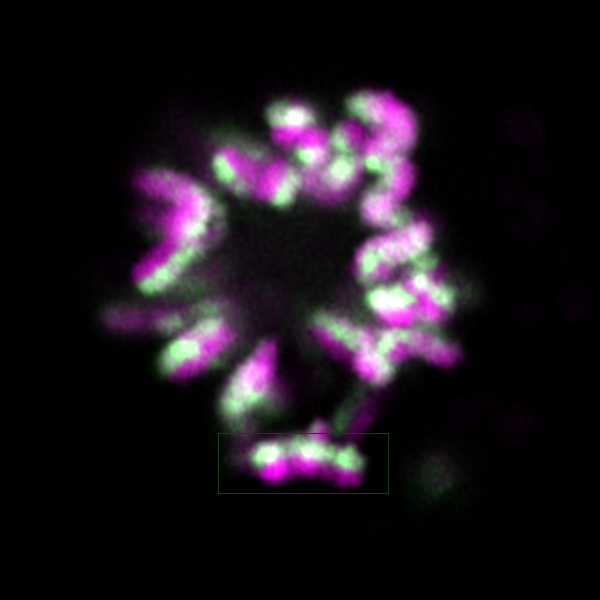

Supplement: Supplementary file 7 — Source Data for Figure 4 [file EMBJ-42-e113475-s001.zip › sd_figure4/panel_c/control/whole_cell/RGB_230316_2108_ctrl_rep1_hemi_stlc_60min_zoom5-06-04.tif_registered_slice59_rotated_600x600_hoechst_edu.tif_draw_roi.tif]

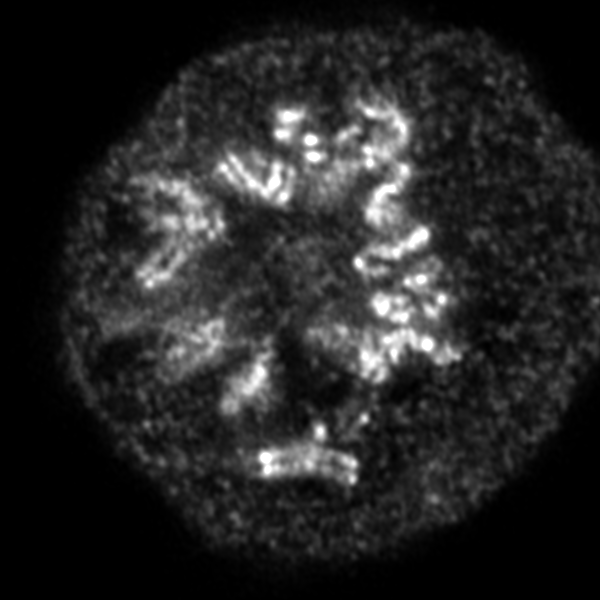

Supplement: Supplementary file 7 — Source Data for Figure 4 [file EMBJ-42-e113475-s001.zip › sd_figure4/panel_c/control/whole_cell/RGB_230316_2108_ctrl_rep1_hemi_stlc_60min_zoom5-06-04.tif_registered_slice59_rotated_600x600_smc4.tif]

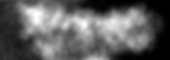

Supplement: Supplementary file 7 — Source Data for Figure 4 [file EMBJ-42-e113475-s001.zip › sd_figure4/panel_c/smc4_dep_120min/insets/16bit_230316_2108_ctrl_2h_smc4_dep_rep1_hemi_stlc_60min_zoom5-05-41.czi #2.tif_registered_slice43_rotated2_edu_170x60inset.tif]

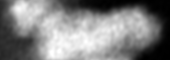

Supplement: Supplementary file 7 — Source Data for Figure 4 [file EMBJ-42-e113475-s001.zip › sd_figure4/panel_c/smc4_dep_120min/insets/16bit_230316_2108_ctrl_2h_smc4_dep_rep1_hemi_stlc_60min_zoom5-05-41.czi #2.tif_registered_slice43_rotated2_hoechst_170x60inset.tif]

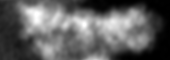

Supplement: Supplementary file 7 — Source Data for Figure 4 [file EMBJ-42-e113475-s001.zip › sd_figure4/panel_c/smc4_dep_120min/insets/16bit_230316_2108_ctrl_2h_smc4_dep_rep1_hemi_stlc_60min_zoom5-05-41.czi #2.tif_registered_slice43_rotated2_hoechst_edu_170x60inset.tif]

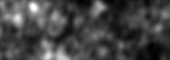

Supplement: Supplementary file 7 — Source Data for Figure 4 [file EMBJ-42-e113475-s001.zip › sd_figure4/panel_c/smc4_dep_120min/insets/16bit_230316_2108_ctrl_2h_smc4_dep_rep1_hemi_stlc_60min_zoom5-05-41.czi #2.tif_registered_slice43_rotated2_inset_all.tif]

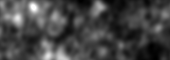

Supplement: Supplementary file 7 — Source Data for Figure 4 [file EMBJ-42-e113475-s001.zip › sd_figure4/panel_c/smc4_dep_120min/insets/16bit_230316_2108_ctrl_2h_smc4_dep_rep1_hemi_stlc_60min_zoom5-05-41.czi #2.tif_registered_slice43_rotated2_inset_smc4.tif]

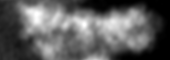

Supplement: Supplementary file 7 — Source Data for Figure 4 [file EMBJ-42-e113475-s001.zip › sd_figure4/panel_c/smc4_dep_120min/insets/RGB_230316_2108_ctrl_2h_smc4_dep_rep1_hemi_stlc_60min_zoom5-05-41.czi #2.tif_registered_slice43_rotated2_edu_170x60inset_rgb.tif]

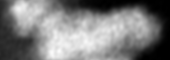

Supplement: Supplementary file 7 — Source Data for Figure 4 [file EMBJ-42-e113475-s001.zip › sd_figure4/panel_c/smc4_dep_120min/insets/RGB_230316_2108_ctrl_2h_smc4_dep_rep1_hemi_stlc_60min_zoom5-05-41.czi #2.tif_registered_slice43_rotated2_hoechst_170x60inset_rgb.tif]

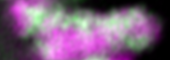

Supplement: Supplementary file 7 — Source Data for Figure 4 [file EMBJ-42-e113475-s001.zip › sd_figure4/panel_c/smc4_dep_120min/insets/RGB_230316_2108_ctrl_2h_smc4_dep_rep1_hemi_stlc_60min_zoom5-05-41.czi #2.tif_registered_slice43_rotated2_hoechst_edu_170x60inset.tif (RGB).tif]

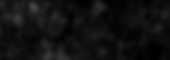

Supplement: Supplementary file 7 — Source Data for Figure 4 [file EMBJ-42-e113475-s001.zip › sd_figure4/panel_c/smc4_dep_120min/insets/RGB_230316_2108_ctrl_2h_smc4_dep_rep1_hemi_stlc_60min_zoom5-05-41.czi #2.tif_registered_slice43_rotated2_inset_smc4.tif]

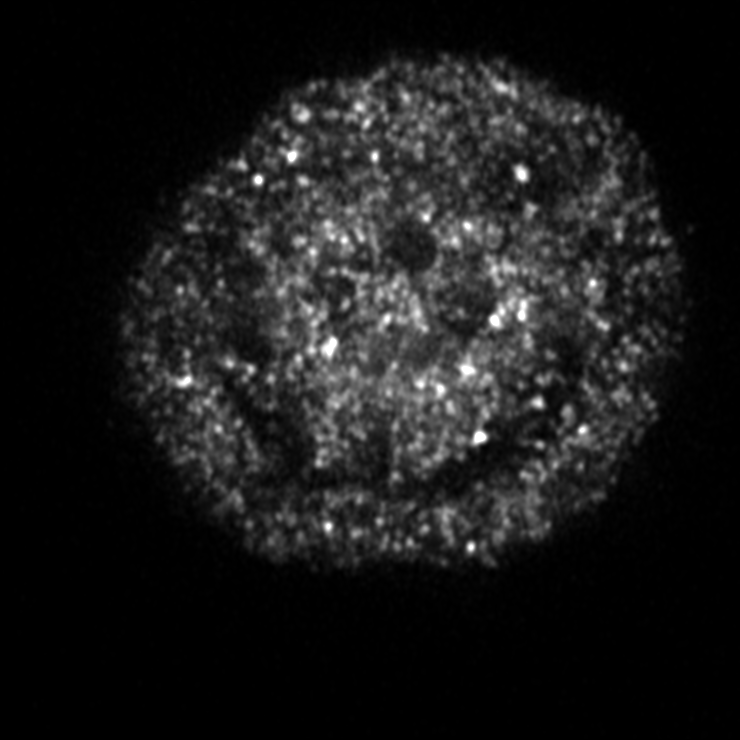

Supplement: Supplementary file 7 — Source Data for Figure 4 [file EMBJ-42-e113475-s001.zip › sd_figure4/panel_c/smc4_dep_120min/whole_cell/16bit_230316_2108_ctrl_2h_smc4_dep_rep1_hemi_stlc_60min_zoom5-05-41.czi #2.tif_registered_slice43_all.tif]
