# Supplementary figures and images for: Cohesin‐mediated DNA loop extrusion resolves sister chromatids in G2 phase (part 3 of 3)
Source: EMBO J. 2023 Jun 26;42(16):e113475. doi: 10.15252/embj.2023113475 (PMC10425840; doi:10.15252/embj.2023113475)

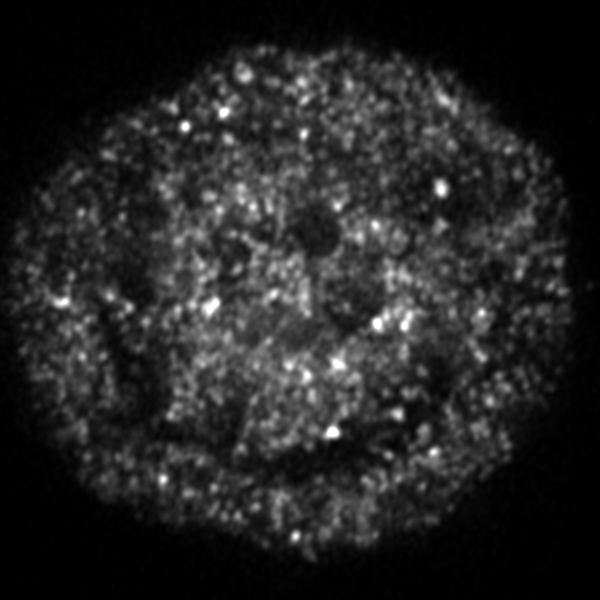

Supplement: Supplementary file 7 — Source Data for Figure 4 [file EMBJ-42-e113475-s001.zip › sd_figure4/panel_c/smc4_dep_120min/whole_cell/16bit_230316_2108_ctrl_2h_smc4_dep_rep1_hemi_stlc_60min_zoom5-05-41.czi #2.tif_registered_slice43_rotated_cropped.tif]

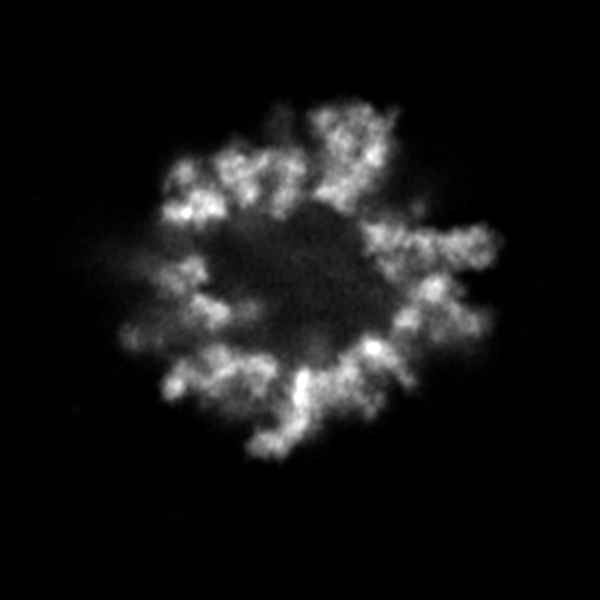

Supplement: Supplementary file 7 — Source Data for Figure 4 [file EMBJ-42-e113475-s001.zip › sd_figure4/panel_c/smc4_dep_120min/whole_cell/16bit_230316_2108_ctrl_2h_smc4_dep_rep1_hemi_stlc_60min_zoom5-05-41.czi #2.tif_registered_slice43_rotated2_hoechst_edu.tif]

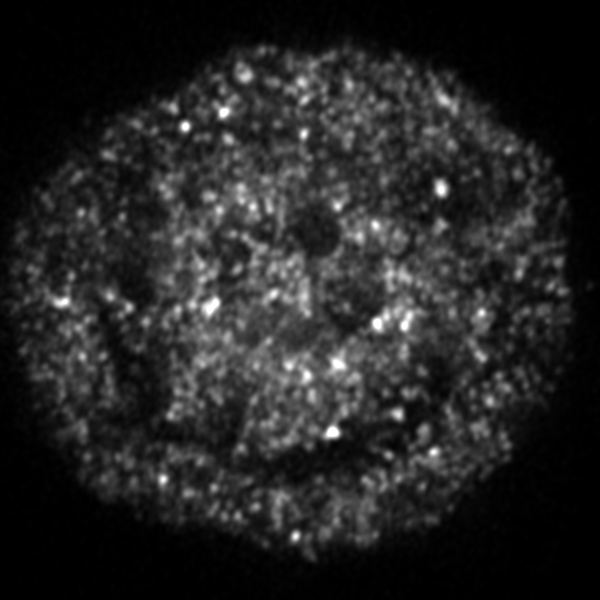

Supplement: Supplementary file 7 — Source Data for Figure 4 [file EMBJ-42-e113475-s001.zip › sd_figure4/panel_c/smc4_dep_120min/whole_cell/16bit_230316_2108_ctrl_2h_smc4_dep_rep1_hemi_stlc_60min_zoom5-05-41.czi #2.tif_registered_slice43_rotated2_smc4.tif]

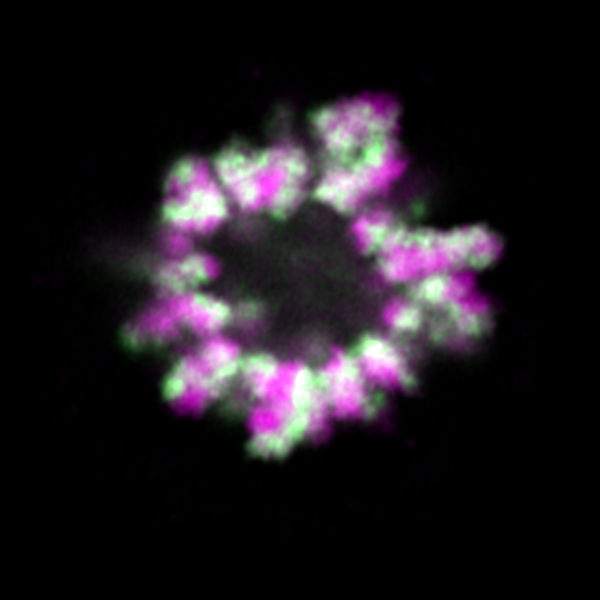

Supplement: Supplementary file 7 — Source Data for Figure 4 [file EMBJ-42-e113475-s001.zip › sd_figure4/panel_c/smc4_dep_120min/whole_cell/RGB_230316_2108_ctrl_2h_smc4_dep_rep1_hemi_stlc_60min_zoom5-05-41.czi #2.tif_registered_slice43_rotated2_hoechst_edu.tif.tif]

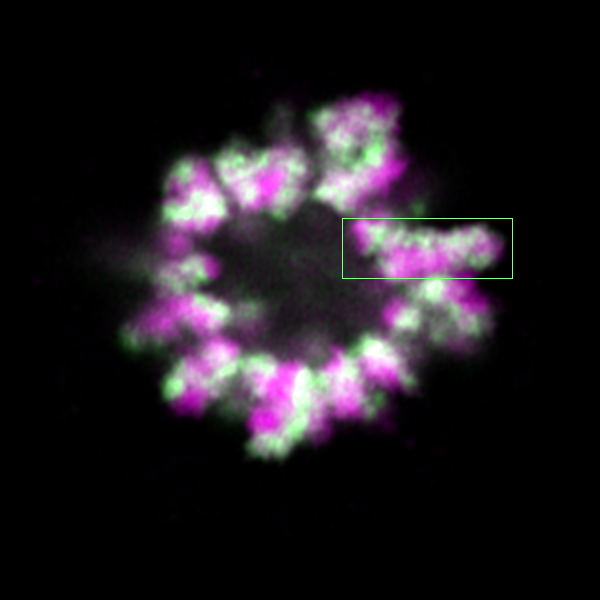

Supplement: Supplementary file 7 — Source Data for Figure 4 [file EMBJ-42-e113475-s001.zip › sd_figure4/panel_c/smc4_dep_120min/whole_cell/RGB_230316_2108_ctrl_2h_smc4_dep_rep1_hemi_stlc_60min_zoom5-05-41.czi #2.tif_registered_slice43_rotated2_hoechst_edu.tif_draw_roi.tif]

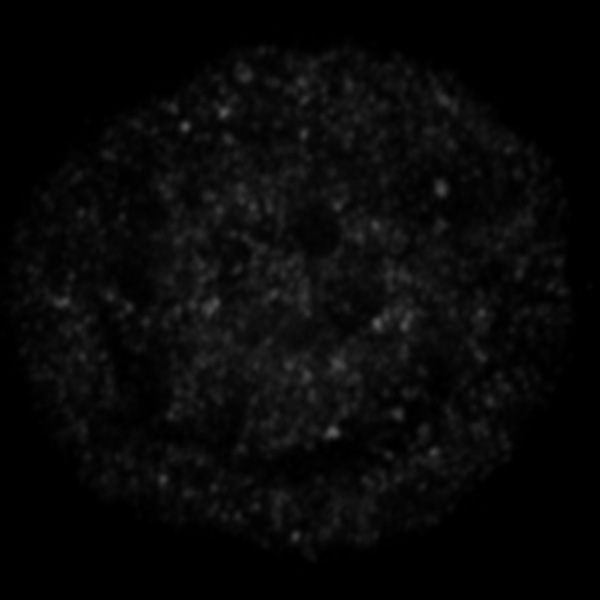

Supplement: Supplementary file 7 — Source Data for Figure 4 [file EMBJ-42-e113475-s001.zip › sd_figure4/panel_c/smc4_dep_120min/whole_cell/RGB_230316_2108_ctrl_2h_smc4_dep_rep1_hemi_stlc_60min_zoom5-05-41.czi #2.tif_registered_slice43_rotated2_smc4.tif]

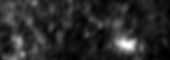

Supplement: Supplementary file 7 — Source Data for Figure 4 [file EMBJ-42-e113475-s001.zip › sd_figure4/panel_c/smc4_dep_240min/insets/16bit_230316_2108_ctrl_4h_smc4_dep_rep1_hemi_stlc_60min_zoom5-19-27.czi #2.tif_registered_inset_170x60_all.tif]

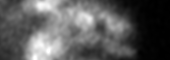

Supplement: Supplementary file 7 — Source Data for Figure 4 [file EMBJ-42-e113475-s001.zip › sd_figure4/panel_c/smc4_dep_240min/insets/16bit_230316_2108_ctrl_4h_smc4_dep_rep1_hemi_stlc_60min_zoom5-19-27.czi #2.tif_registered_inset_170x60_edu.tif]

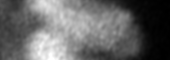

Supplement: Supplementary file 7 — Source Data for Figure 4 [file EMBJ-42-e113475-s001.zip › sd_figure4/panel_c/smc4_dep_240min/insets/16bit_230316_2108_ctrl_4h_smc4_dep_rep1_hemi_stlc_60min_zoom5-19-27.czi #2.tif_registered_inset_170x60_hoechst.tif]

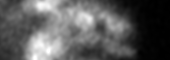

Supplement: Supplementary file 7 — Source Data for Figure 4 [file EMBJ-42-e113475-s001.zip › sd_figure4/panel_c/smc4_dep_240min/insets/16bit_230316_2108_ctrl_4h_smc4_dep_rep1_hemi_stlc_60min_zoom5-19-27.czi #2.tif_registered_inset_170x60_hoechst_edu.tif]

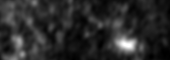

Supplement: Supplementary file 7 — Source Data for Figure 4 [file EMBJ-42-e113475-s001.zip › sd_figure4/panel_c/smc4_dep_240min/insets/16bit_230316_2108_ctrl_4h_smc4_dep_rep1_hemi_stlc_60min_zoom5-19-27.czi #2.tif_registered_inset_170x60_Smc4.tif]

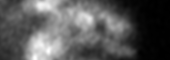

Supplement: Supplementary file 7 — Source Data for Figure 4 [file EMBJ-42-e113475-s001.zip › sd_figure4/panel_c/smc4_dep_240min/insets/RGB_230316_2108_ctrl_4h_smc4_dep_rep1_hemi_stlc_60min_zoom5-19-27.czi #2.tif_registered_inset_170x60_edu.tif]

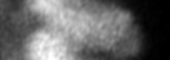

Supplement: Supplementary file 7 — Source Data for Figure 4 [file EMBJ-42-e113475-s001.zip › sd_figure4/panel_c/smc4_dep_240min/insets/RGB_230316_2108_ctrl_4h_smc4_dep_rep1_hemi_stlc_60min_zoom5-19-27.czi #2.tif_registered_inset_170x60_hoechst.tif]

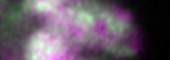

Supplement: Supplementary file 7 — Source Data for Figure 4 [file EMBJ-42-e113475-s001.zip › sd_figure4/panel_c/smc4_dep_240min/insets/RGB_230316_2108_ctrl_4h_smc4_dep_rep1_hemi_stlc_60min_zoom5-19-27.czi #2.tif_registered_inset_170x60_hoechst_edu.tif (RGB).tif]

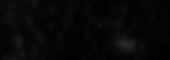

Supplement: Supplementary file 7 — Source Data for Figure 4 [file EMBJ-42-e113475-s001.zip › sd_figure4/panel_c/smc4_dep_240min/insets/RGB_230316_2108_ctrl_4h_smc4_dep_rep1_hemi_stlc_60min_zoom5-19-27.czi #2.tif_registered_inset_170x60_Smc4.tif]

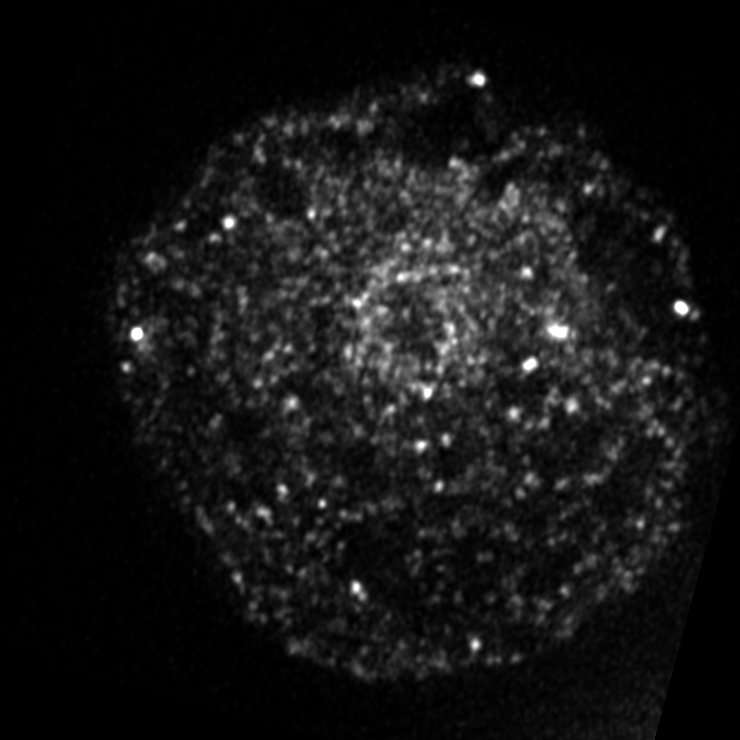

Supplement: Supplementary file 7 — Source Data for Figure 4 [file EMBJ-42-e113475-s001.zip › sd_figure4/panel_c/smc4_dep_240min/whole_cell/16bit_230316_2108_ctrl_4h_smc4_dep_rep1_hemi_stlc_60min_zoom5-19-27.czi #2.tif_registered_rotate_all.tif]

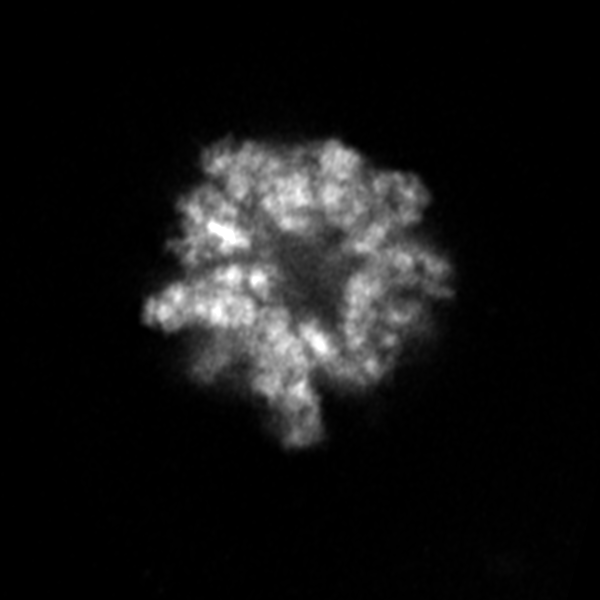

Supplement: Supplementary file 7 — Source Data for Figure 4 [file EMBJ-42-e113475-s001.zip › sd_figure4/panel_c/smc4_dep_240min/whole_cell/16bit_230316_2108_ctrl_4h_smc4_dep_rep1_hemi_stlc_60min_zoom5-19-27.czi #2.tif_registered_rotate_hoechst_edu.tif]

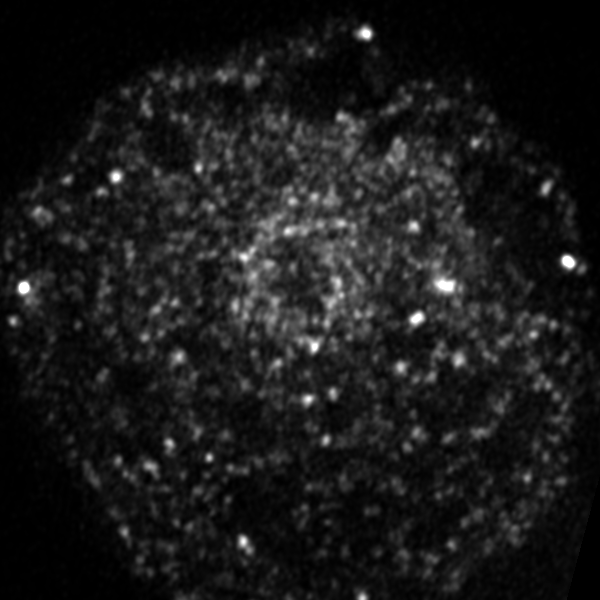

Supplement: Supplementary file 7 — Source Data for Figure 4 [file EMBJ-42-e113475-s001.zip › sd_figure4/panel_c/smc4_dep_240min/whole_cell/16bit_230316_2108_ctrl_4h_smc4_dep_rep1_hemi_stlc_60min_zoom5-19-27.czi #2.tif_registered_rotate_smc4.tif]

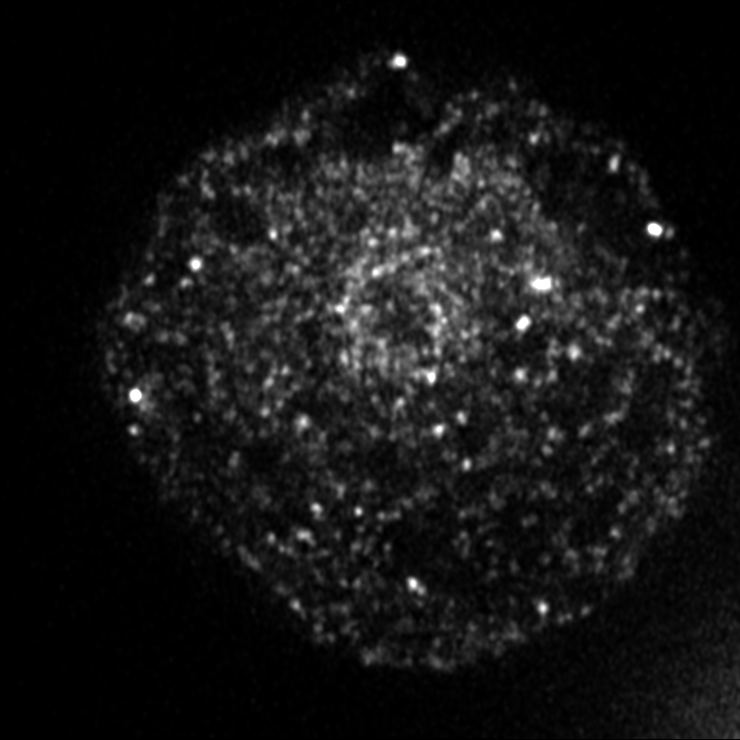

Supplement: Supplementary file 7 — Source Data for Figure 4 [file EMBJ-42-e113475-s001.zip › sd_figure4/panel_c/smc4_dep_240min/whole_cell/16bit_230316_2108_ctrl_4h_smc4_dep_rep1_hemi_stlc_60min_zoom5-19-27.czi #2.tif_registered_slice51_all.tif]

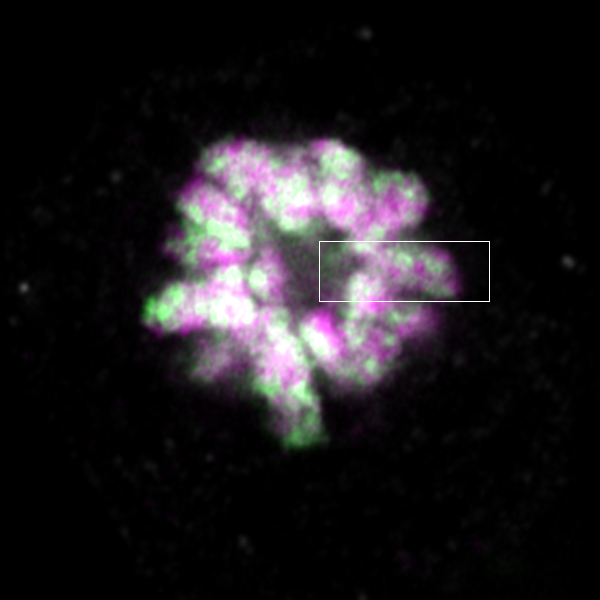

Supplement: Supplementary file 7 — Source Data for Figure 4 [file EMBJ-42-e113475-s001.zip › sd_figure4/panel_c/smc4_dep_240min/whole_cell/RGB_230316_2108_ctrl_4h_smc4_dep_rep1_hemi_stlc_60min_zoom5-19-27.czi #2.tif_registered_rotate.tif_draw_roi.tif]

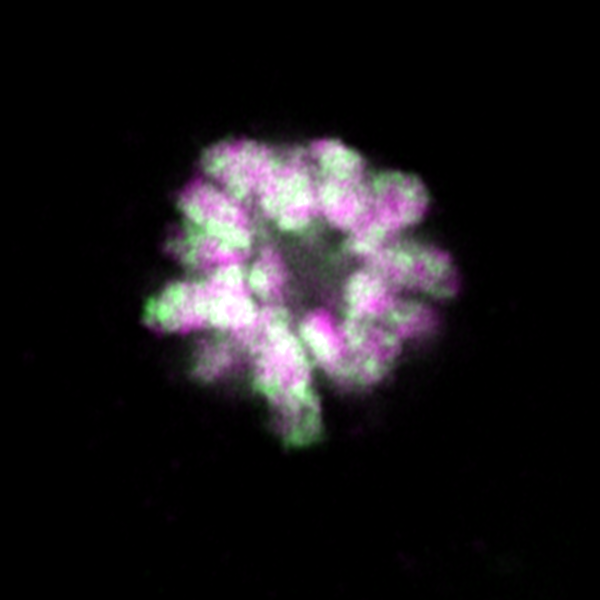

Supplement: Supplementary file 7 — Source Data for Figure 4 [file EMBJ-42-e113475-s001.zip › sd_figure4/panel_c/smc4_dep_240min/whole_cell/RGB_230316_2108_ctrl_4h_smc4_dep_rep1_hemi_stlc_60min_zoom5-19-27.czi #2.tif_registered_rotate_hoechst_edu.tif.tif]

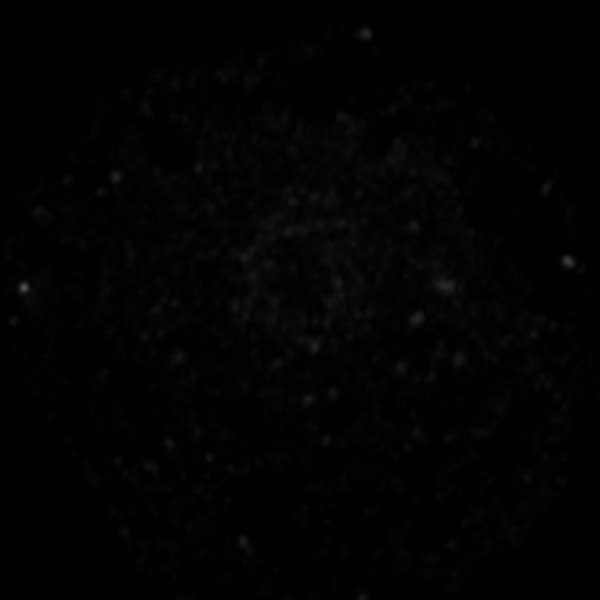

Supplement: Supplementary file 7 — Source Data for Figure 4 [file EMBJ-42-e113475-s001.zip › sd_figure4/panel_c/smc4_dep_240min/whole_cell/RGB_230316_2108_ctrl_4h_smc4_dep_rep1_hemi_stlc_60min_zoom5-19-27.czi #2.tif_registered_rotate_smc4.tif]
