# Supplementary material for: Cohesin‐mediated DNA loop extrusion resolves sister chromatids in G2 phase
Source: EMBO J. 2023 Jun 26;42(16):e113475. doi: 10.15252/embj.2023113475 (PMC10425840; doi:10.15252/embj.2023113475)
Supplement: Supplementary file 9 [file EMBJ-42-e113475-s009.pdf]

THE EMBO JOURNAL

Cohesin-mediated DNA loop extrusion resolves sister chromatids in G2 phase

Paul Batty, Christoph Langer, Zsuzsanna Takacs, Wen Tang, Claudia Blaukopf, Jan-Michael Peters, and Daniel Gerlich
DOI: 10.15252/embj.2023113475

Corresponding author(s): Daniel Gerlich (daniel.gerlich@imba.oeaw.ac.at)

Review Timeline:

Submission Date:	10th Jan 23
Editorial Decision:	9th Feb 23
Revision Received:	29th Mar 23
Editorial Decision:	23rd May 23
Revision Received:	26th May 23
Accepted:	14th Jun 23

Editor: Hartmut Vodermaier

Transaction Report:

(Note: With the exception of the correction of typographical or spelling errors that could be a source of ambiguity, letters and reports are not edited. Depending on transfer agreements, referee reports obtained elsewhere may or may not be included in this compilation. Referee reports are anonymous unless the Referee chooses to sign their reports.)

Dr. Daniel W. Gerlich
Institute of Molecular Biotechnology of the Austrian Academy of Sciences (IMBA)
Dr. Bohrgasse 3
Vienna 1030
Austria

9th Feb 2023

Re: EMBOJ-2023-113475
Cohesin-mediated DNA loop extrusion resolves sister chromatids in G2 phase

Dear Daniel,

Thank you for submitting your study on cohesin-driven G2 sister chromatid resolution to The EMBO Journal. It has now been assessed by three expert referees, whose comments are copied below for your information. As you will see, all referees consider your work interesting and important, and are therefore supportive of publication pending revision of a limited number of specific points noted in the three reports. I would therefore like to invite you to address these points and resubmit a revised manuscript, taking into account the detailed guidelines below and on our website.

I should remind you that it is our policy to allow only a single round of (major) revision, making it important to carefully respond to all points raised at this stage; therefore, please do not hesitate to contact me in case you would like to discuss any of the issues raised by the reviewers. Also, should revision require more time than our default three-months revision period, we would be open to offering an extension, during which our 'scooping protection' (meaning that competing work appearing elsewhere in the meantime will not affect our considerations of your study) would of course remain valid.

Thank you again for the opportunity to consider this work for The EMBO Journal, and I look forward to your revision!

With kind regards,

Hartmut

Hartmut Vodermaier, PhD
Senior Editor, The EMBO Journal
h.vodermaier@embojournal.org

*** PLEASE NOTE: All revised manuscript are subject to initial checks for completeness and adherence to our formatting guidelines. Revisions may be returned to the authors and delayed in their editorial re-evaluation if they fail to comply to the following requirements (see also our Guide to Authors for further information):

1) Every manuscript requires a Data Availability section (even if only stating that no deposited datasets are included). Primary datasets or computer code produced in the current study have to be deposited in appropriate public repositories prior to resubmission, and reviewer access details provided in case that public access is not yet allowed. Further information: embopress.org/page/journal/14602075/authorguide#dataavailability

2) Each figure legend must specify
- size of the scale bars that are mandatory for all micrograph panels
- the statistical test used to generate error bars and P-values
- the type error bars (e.g., S.E.M., S.D.)
- the number (n) and nature (biological or technical replicate) of independent experiments underlying each data point
- Figures may not include error bars for experiments with n<3; scatter plots showing individual data points should be used instead.

3) Revised manuscript text (including main tables, and figure legends for main and EV figures) has to be submitted as editable text file (e.g., .docx format). We encourage highlighting of changes (e.g., via text color) for the referees' reference.

4) Each main and each Expanded View (EV) figure should be uploaded as individual production-quality files (preferably in .eps, .tif, .jpg formats). For suggestions on figure preparation/layout, please refer to our Figure Preparation Guidelines: http://bit.ly/EMBOPressFigurePreparationGuideline

5) Point-by-point response letters should include the original referee comments in full together with your detailed responses to them (and to specific editor requests if applicable), and also be uploaded as editable (e.g., .docx) text files.

6) Please complete our Author Checklist, and make sure that information entered into the checklist is also reflected in the manuscript; the checklist will be available to readers as part of the Review Process File. A download link is found at the top of our Guide to Authors: embopress.org/page/journal/14602075/authorguide

7) All authors listed as (co-)corresponding need to deposit, in their respective author profiles in our submission system, a unique ORCiD identifier linked to their name. Please see our Guide to Authors for detailed instructions.

8) Please note that supplementary information at EMBO Press has been superseded by the 'Expanded View' for inclusion of additional figures, tables, movies or datasets; with up to five EV Figures being typeset and directly accessible in the HTML version of the article. For details and guidance, please refer to:
embopress.org/page/journal/14602075/authorguide#expandedview

9) Digital image enhancement is acceptable practice, as long as it accurately represents the original data and conforms to community standards. If a figure has been subjected to significant electronic manipulation, this must be clearly noted in the figure legend and/or the 'Materials and Methods' section. The editors reserve the right to request original versions of figures and the original images that were used to assemble the figure. Finally, we generally encourage uploading of numerical as well as gel/blot image source data; for details see: embopress.org/page/journal/14602075/authorguide#sourcedata

At EMBO Press, we ask authors to provide source data for the main manuscript figures. Our source data coordinator will contact you to discuss which figure panels we would need source data for and will also provide you with helpful tips on how to upload and organize the files.

Further information is available in our Guide For Authors:
https://www.embopress.org/page/journal/14602075/authorguide

In the interest of ensuring the conceptual advance provided by the work, we recommend submitting a revision within 3 months (10th May 2023). Please discuss the revision progress ahead of this time with the editor if you require more time to complete the revisions. Use the link below to submit your revision:

Link Not Available

Referee #1:

This manuscript by the Gerlich laboratory explores a role of cohesin in resolving sister chromatids prior to mitosis. Cohesin has initially been identified as a key factor for sister chromatid cohesion from S phase to mitosis. Later on, it was appreciated that cohesin also supports DNA loop extrusion, which raises the possibility that cohesin has antagonistic activities by resolving sister DNA molecules as well as holding them together. Separating the two functions however is challenging. Here, the authors elegantly combine complementary technologies (for DNA labelling, protein depletion, cell synchronization and the measurement of DNA organization) to demonstrate cohesin's ability to resolve sister DNA molecules and suggest that this function initiates chromosome segregation in the G2 phase of the cell cycle. While the physiological relevance of sister DNA unmixing in G2 remains unclear, the findings strongly support the notion that DNA loop extrusion promotes DNA unmixing and-when arm cohesion is weakened-is sufficient for the formation and separation of mitotic chromosome-like axial structures. This capacity thus appears more widely shared (possibly universal) amongst SMC complexes.
The experiments are conducted and analyzed at a high technical level. The work is presented clearly and concisely. Altogether, this is an excellent study that delineates the minimal requirements for the formation of mitotic chromosomes. Only minor issues should be addressed before publication (see further below).

Can the authors provide an estimate for the level of cohesive cohesin remaining after sororin depletion? Can those levels be titrated? How much chromosome arm cohesion would be needed to block axis separation?

Does condensin depletion reduce 'genomic resolution' similarly in G2 and in prometaphase? (it is hard to read the small bar in fig 4b). If not, what might be the explanation for any difference? Related to this point: Can the authors address whether continuous loop extrusion is needed to maintain resolved sister DNA molecules (e.g. by removing LE-cohesin from G2 cells or condensin from mitotic cells)?

The reference table contains an incomplete entry (Schneider et al. 2022; journal name lacking).

The authors may want to consider citing a paper showing that bacterial SMC complexes (MukBEF) (in addition to helping to resolve chromosomes) can also form axis-like structures when slightly overexpressed: Mäkelä and Sherratt, 2020.

The first parts of the results section use the word 'extensive' multiple times to describe the extent of sister chromatid resolution, but without qualifying what 'extensive' means. Adding numbers or a reference to the results of the second part (scs-HiC) might be helpful already at this point.

Page 6, top of 2nd column: confusing statement. Again, it would be helpful to provide numbers (separation score for wapl and wapl/nipbl) or alternatively move this statement to the discussion.

The order of panels in fig 1 (prometaphase before G2 cells) is potentially confusing. Possibly, splitting the figure into two separate figures (one showing the proof of principle in prometaphase; and another extending the findings to G2 cells) might be helpful.

In fig 2D: the order of the mutations does not match their order in panels a, b, and c. Consider reordering.

Referee #2:

The work of Patty et al. "Cohesin-mediated DNA loop extrusion resolves sister chromatids in G2 phase" reveals a role for cohesin in promoting the segregation of individual sister chromatids during G2.
During the cell cycle, the 3D organization of chromatin undergoes profound changes that depend in part on two SMC complexes, cohesin and condensin. Cohesin was initially identified as the promoter of sister chromatid attachment during replication. This complex, like condensin, is also capable of generating DNA loops. These loops are thought to be mediated by a process called loop extrusion. Cohesin-dependent loops divide the genome into TADs during interphase, whereas loops established by condensin during mitosis are required to condense chromosomes.
At the beginning of mitosis, cohesin dissociates from the entire chromosome arm by a process mediated by WAPL. During mitosis, condensin associates with chromatids to ensure chromosome condensation. Condensin-mediated chromosome condensation is accompanied by sister chromatid resolution. However, it remains unclear how condensin and cohesin activities contribute to sister chromatid resolution.

In the present study, the authors apply a microscopic approach that exploits click chemistry to label DNA strands that have incorporated F-ara-EdU (as developed in Hao et al, genome biology, 2021 that could be cited), allowing them to image individual sister chromatids during interphase and mitosis. Using a simple but robust correlation analysis, they computed "separation scores" between individual sister chromatids in G2 and prometa cells in WT and mutant conditions. They use this metric to quantify the segregation of individual chromatids at different stages of the cell cycle and in cohesive regulatory factors. They back up the imaging approach with a previously developed sister chromatid Hi-C protocol to monitor sister DNA interaction frequencies and provide evidence that cohesin is capable of segregating sister chromatids during G2. This is likely through its DNA extrusion activity.

An elegant experiment performed by the authors further revealed that hyperactive cohesin (induced by WAPL depletion) produces prophase-like sister chromatid resolution in G2-arrested D sororin cells. These data unequivocally demonstrate that, during mitosis, sister chromatid resolution is induced by forces applied by the loop extrusion process. The authors thus provide a comprehensive body of evidence on the contribution of cohesin-mediated loop formation in mammalian chromosome segregation.

The manuscript is very interesting, concise, clearly written and seems of high technical quality. I have no specific major concerns. One could wonder what happen to the cohesins involved in cohesion maintenance upon WAPL depletion. Are they removed ? or pushed by extruding cohesins? The experiment may be too long to tackle but maybe the authors could comment on that.

The discussion regarding other species is not that convincing. Freeman 2000 supports segregation of rDNA by condensin, but doesn't show cohesin-mediated segregation (nor resolution, unless I'm mistaken). In general, I am not sure about papers demonstrating that cohesin contribute to segregation, though they do compact G2 chromosomes (Lazar 2017, Schalbeter 2017). Condensin was shown to actively contribute to segregation through the formation of loops and possibly removal of entanglements (Lazar 2017, Paul 2018) and potentially also play a role in decatenation of DNA (eg Dyson 2021). Also, I am not sure that any experiment supports SMC-mediated segregation in archaea (the Palecek 2015 reference shows that SMCs are conserved), though SMCs shape chromosomes in archaea (Cockram 2021, Takemata 2019). In bacteria, Marbouty 2015 also show SMC-related segregation by HiC and imaging in B subtilis. Perhaps some work regarding segregation in E. coli could also be mentioned if relevant. The discussion could (and should) therefore be enriched by making more specific statements about other domains of life rather than broad generalizations based on a single set of experiments.

Referee #3:

The resolution of sister chromatids is a fundamental requirement for faithful chromosome segregation in higher eukaryotes. Several studies focused on the resolution mechanisms during early mitosis, but whether and how sister chromatids are resolved before mitotic entry is underexplored.

In this exciting manuscript, Batty and co-workers provide compelling evidence that cohesin complexes can resolve sister chromatids to a considerable extent before mitotic entry. Distinct SMC complexes are considered critical to different aspects of genome organisation. However, they all share some biochemical activities, which raises the intriguing question of how the functional divergence is brought about in vivo. Here, the authors use an elegant experimental layout, combined with unique approaches to distinguish genetically identical sister chromatids, to demonstrate that cohesin complexes can resolve DNA molecules to a large extent, even before mitotic entry. They observe that chromatids separation is decreased upon loss of cohesin loop-extrusion (ΔNIPBL) and enhanced in the presence of hyperactive cohesin (ΔWAPL), with condensed and resolved chromatids organised around a single cohesin-axis. Strikingly, enhanced loop extrusion coupled with the loss of cohesion (ΔSororin- ΔWAPL) causes the axes of sister chromatids to split, resulting in G2 chromosomes that highly resemble late prophase chromosomes. Collectively, these findings support that a regulated balance of cohesive and loop-extruding cohesin molecules can resolve sister chromatids before mitosis, an activity previously attributed to condensins and topoisomerases during mitosis.

Overall, the experiments are very well designed, the technologies applied are top-edge, and the data is high quality. The conclusions are novel and challenge current dogmas: 1) They provide evidence that sister chromatid resolution is quite significant before mitotic entry, and 2) they support that this process is primarily mediated by cohesin complexes, previously believed to counteract the resolution process (via cohesive cohesins).

These findings have broad implications for our understanding of sister chromatid resolution and how the activities associated with SMC complexes are regulated in vivo. The concepts that emerge from this study will significantly interest the chromosome biology and genome organization fields. In a broader context, the conclusions are also thought-provoking to anyone interested in the functional divergence of related proteins/complexes.

We do have a few issues that we would like the authors to consider before publication:

Major points

1) The authors established a new protocol for imaging and distinction of sister chromatids. Whereas the presented protocol is an improvement over previously established methods (e.g. Nagasaka et al., 2016), it has the significant caveat that the presence of "two objects" is inferred based on the absence of a signal. This methodology could be problematic if staining is less optimal for unknown reasons. If experimentally possible, the authors should validate their major findings using a strategy where both are individually labelled. Or at least properly acknowledge this intrinsic caveat of their methodology when comparing their method with previous ones.

2) The analysis lacks a reference point to what would be "no resolution" in interphase chromatin so that the significance of the extent of the reduction observed upon NIBL depletion can be better understood. E.g., use top2 depletion or ICRF treatment to estimate what would be the "unresolved" state. Comparing it to the prometaphase values upon depletion of SMC4 may not be the best reference, as the state of chromosome compaction may impact the quantification output.

3) It remains untested how much of the enhanced cohesin activity in G2 could compensate for the "canonical resolution machinery" in mitosis. The authors performed the proper experiment to address this issue (EV5c) but restricted their analysis to G2 cells. What happens in mitosis in cells double depleted for SMC4 and WAPL? Can WAPL depletion restore the absence of resolution observed by metaphase in the absence of SMC4 (even if around a "single cohesin-axis")? It would further validate the author's claim of extensive DNA resolution (to the maximal level required) if it does. If not, it would raise interesting questions on how mitosis may bring about special requirements for chromatid resolution. Either result would be interesting. The same could be asked for the WAPL+Sor depletion (although performing the triple depletion may be more experimentally demanding). Substantiating how much of their findings in G2 can rescue condensin-mediated mitotic resolution would significantly improve their striking observations.

Minor issues:

1) Although the rationale is very well designed for what the authors wish to test, each figure presents experiments in parallel that were performed differently. For example, in Fig. 1, depletion of NIBL is timed to after S-phase (a must to allow for cohesion establishment), but SMC4 depletion occurs before S-phase. The differences in experimental layout do not preclude author's conclusions (on the contrary) but we would suggest that each figure includes a small scheme so that each experimental layout

for each condition is more readily identified. This would actually do better justice to the clever experimental designs employed throughout the manuscript.

2) The double-depleted WAPL-Sororin cells display sister chromatid axis separation. From the images in Fig2, it looks like the axes overlap in some regions. Do the authors know if those regions are cohered and, if so, whether they coincide with the centromeres? It may be interesting to add a comment on this and how these regions could be brought about (cohesin protection will certainly be different in interphase).

3) "Thus, in the presence of arm cohesion, DNA loop extrusion promotes a highly ASYMMETRIC displacement of sister chromatid DNA away from a central cohesin axis." From Fig3b, the displacement of sister chromatids looks rather symmetrical, as the two peaks localise at a similar distance from the central cohesin axis. We suggest rephrasing the sentence to improve clarity.

4) The authors state that depletion of WAPL (hyperactive cohesin) is more robust in promoting resolution than Sororin depletion (removing cohesin). However, the graph in Fig. 2d does not present statistical analysis between these two conditions.

5) The authors use Mann Whitney U test for comparative analysis throughout the manuscript, which is ideal to compare 2 groups. Using a non-parametric one-way ANOVA equivalent (e.g. Kruskal-Wallis) would be possibly more appropriate for multiple comparisons between 3 or more experimental conditions.

6) In the caption of Fig3, the last line reports: "Yellow boxes indicate inset regions". There are no yellow boxes in the figure.

7) In page 7, the last sentence of the "Genomic range of sister chromatids resolution" section says: " [...] chromatin loop extrusion is a fundamental mechanism by which genomes are segregated." The sentence can be misinterpreted as segregation is often referred to the poleward movement of chromatids during anaphase. Maybe using "separated" or "resolved" instead would be more appropriate.

We thank the referees for their interest in our work and for their helpful comments regarding how to improve our manuscript. We have addressed each of the referee comments in a point-by-point response below.

We have provided referee access to the deposited scsHi-C data. To access the data deposited with the GEO accession number GSE227694, the referees should go to:

https://www.ncbi.nlm.nih.gov/geo/query/acc.cgi?acc=GSE227694

Referee #1:

This manuscript by the Gerlich laboratory explores a role of cohesin in resolving sister chromatids prior to mitosis. Cohesin has initially been identified as a key factor for sister chromatid cohesion from S phase to mitosis. Later on, it was appreciated that cohesin also supports DNA loop extrusion, which raises the possibility that cohesin has antagonistic activities by resolving sister DNA molecules as well as holding them together. Separating the two functions however is challenging. Here, the authors elegantly combine complementary technologies (for DNA labelling, protein depletion, cell synchronization and the measurement of DNA organization) to demonstrate cohesin's ability to resolve sister DNA molecules and suggest that this function initiates chromosome segregation in the G2 phase of the cell cycle. While the physiological relevance of sister DNA unmixing in G2 remains unclear, the findings strongly support the notion that DNA loop extrusion promotes DNA unmixing and-when arm cohesion is weakened-is sufficient for the formation and separation of mitotic chromosome-like axial structures. This capacity thus appears more widely shared (possibly universal) amongst SMC complexes.

The experiments are conducted and analyzed at a high technical level. The work is presented clearly and concisely. Altogether, this is an excellent study that delineates the minimal requirements for the formation of mitotic chromosomes.

Only minor issues should be addressed before publication (see further below).

Can the authors provide an estimate for the level of cohesive cohesin remaining after sororin depletion? Can those levels be titrated? How much chromosome arm cohesion would be needed to block axis separation?

To estimate the level of cohesive cohesin after Sororin depletion, we performed quantitative immunofluorescence, using the same RNAi conditions as in our original experiments, and a previously published Sororin-AID cell line treated with auxin as a reference control (Fig. EV7b, c). These experiments show that after Sororin RNAi nuclear Sororin levels are reduced to 16 ± 5.8 % of the levels of control cells, which we can use as an estimate for the amount of cohesive cohesin remaining in these cells. The new data are presented in the main text and the new data figure.

Regarding the amount of cohesion required to block axis separation, we performed an experiment to deplete Sororin to varying extents in WAPL depleted G2 cells and subsequently quantified the Sororin protein abundance by immunofluorescence, in addition to the extent of cohesin axis separation by measuring segment lengths of chromosomes that are split or non-split. At intermediate Sororin levels, local regions within the cohesin axes begin to split, but when Sororin protein levels are reduced to around 30 % of the levels of control cells, still more than 50 % of cohesin axes are single axes. At lower Sororin levels, most sister axes split. These observations are presented in the main text and the detailed quantitative data is shown in the new data figure (Fig. EV7d, e).

Does condensin depletion reduce 'genomic resolution' similarly in G2 and in prometaphase? (it is hard to read the small bar in fig 4b). If not, what might be the explanation for any difference?

We thank the referee for their suggestion to compare sister chromatid resolution of condensin-depleted cells in G2 and prometaphase. The genomic resolution for SMC4 depleted G2 cells is 2.6 Mb, compared to 1.3 Mb for SMC4 depleted prometaphase cells. We do not know what causes this two-fold difference, but speculate that due to the continued presence of cohesin-mediated loop extrusion in G2 cells depleted of condensins, sister chromatids are resolved more than in prometaphase cells depleted of condensins, where most cohesin has dissociated from chromatin arms.

Related to this point:

Can the authors address whether continuous loop extrusion is needed to maintain resolved sister DNA molecules (e.g. by removing LE-cohesin from G2 cells or condensin from mitotic cells)?

We thank the referee for their suggestion to test whether continuous loop extrusion is necessary to maintain as well as generate resolved sister chromatids. To address this point, we first generated prometaphase cells with resolved sister chromatids and normal chromosome morphology, before acutely depleting condensins to assess how sister chromatid resolution changes over time in the absence of a factor that is able to continuously extrude chromatin loops (Fig. 4c-e). Using our sister-chromatid labelling assay and separation score quantification, we found that upon condensin depletion in prometaphase cells, sister chromatid resolution was strongly reduced both 2 and 4 h after induction of depletion, and sister chromatid intermixing was increased. These results demonstrate that the continued presence of condensins, and likely the loop extruding activity that condensins confer, is therefore required not only to generate resolved sister chromatids, but also for their maintenance. The new data and quantification are presented in the main text and the new data figure.

The reference table contains an incomplete entry (Schneider et al. 2022; journal name lacking).

We have updated the reference with the journal information.

The authors may want to consider citing a paper showing that bacterial SMC complexes (MukBEF) (in addition to helping to resolve chromosomes) can also form axis-like structures when slightly overexpressed: Mäkelä and Sherratt, 2020.

We thank the referee for pointing out this reference and refer to it in our revised discussion. We also extended the discussion to give more specific examples of how SMC proteins in bacteria relate to our observations.

The first parts of the results section use the word 'extensive' multiple times to describe the extent of sister chromatid resolution, but without qualifying what 'extensive' means. Adding numbers or a reference to the results of the second part (scs-HiC) might be helpful already at this point.

We have qualified the use of the word 'extensively' in the requisite section and provided numbers to quantify the extent of resolution where appropriate.

Page 6, top of 2nd column: confusing statement. Again, it would be helpful to provide numbers (separation score for wapl and wapl/nipbl) or alternatively move this statement to the discussion.

We have added a qualifying sentence with numbers comparing WAPL depleted and WAPL/Sororin depleted G2 cells with late prophase cells to clarify this statement.

The order of panels in fig 1 (prometaphase before G2 cells) is potentially confusing. Possibly, splitting the figure into two separate figures (one showing the proof of principle in prometaphase; and another extending the findings to G2 cells) might be helpful.

We thank the referee for their suggestion. However, we would like to show side-by-side comparisons of control and perturbations in prometaphase and G2 next to each other, to facilitate comparison of the different phenotypes.

In fig 2D: the order of the mutations does not match their order in panels a, b, and c. Consider reordering.

We thank the referee for noticing this inconsistency and have changed the order of the perturbations in the quantification panel such that it matches the order in the microscopy images.

Referee #2:

The work of Batty et al. "Cohesin-mediated DNA loop extrusion resolves sister chromatids in G2 phase" reveals a role for cohesin in promoting the segregation of individual sister chromatids during G2.
During the cell cycle, the 3D organization of chromatin undergoes profound changes that depend in part on two SMC complexes, cohesin and condensin. Cohesin was initially identified as the promoter of sister chromatid attachment during replication. This complex, like condensin, is also capable of generating DNA loops. These loops are thought to be mediated by a process called loop extrusion. Cohesin-dependent loops divide the genome into TADs during interphase, whereas loops established by condensin during mitosis are required to condense chromosomes. At the beginning of mitosis, cohesin dissociates from the entire chromosome arm by a process mediated by WAPL. During mitosis, condensin associates with chromatids to ensure chromosome condensation. Condensin-mediated chromosome condensation is accompanied by sister chromatid resolution. However, it remains unclear how condensin and cohesin activities contribute to sister chromatid resolution.

In the present study, the authors apply a microscopic approach that exploits click chemistry to label DNA strands that have incorporated F-ara-EdU (as developed in Hao et al, genome biology, 2021 that could be cited), allowing them to image individual sister chromatids during interphase and mitosis. Using a simple but robust correlation analysis, they computed "separation scores" between individual sister chromatids in G2 and prometa cells in WT and mutant conditions. They use this metric to quantify the segregation of individual chromatids at different stages of the cell cycle and in cohesive regulatory factors. They back up the imaging approach with a previously developed sister chromatid Hi-C protocol to monitor sister DNA interaction frequencies and provide evidence that cohesin is capable of segregating sister chromatids during G2. This is likely through its DNA extrusion activity.

An elegant experiment performed by the authors further revealed that hyperactive cohesin (induced by WAPL depletion) produces prophase-like sister chromatid resolution in G2-arrested D sororin cells. These data unequivocally demonstrate that, during mitosis, sister chromatid resolution is induced by forces applied by the loop extrusion process. The authors thus provide a comprehensive body of evidence on the contribution of cohesin-mediated loop formation in mammalian chromosome segregation.

The manuscript is very interesting, concise, clearly written and seems of high technical quality. I have no specific major concerns.

One could wonder what happen to the cohesins involved in cohesion maintenance upon WAPL depletion. Are they removed? or pushed by extruding cohesins? The experiment may be too long to tackle but maybe the authors could comment on that.

We thank the referee for the suggestion and mention this idea as a future perspective in the discussion. That we see a single cohesin axis upon WAPL depletion would argue against a significant reduction in cohesion between sisters. The extent to which cohesive cohesin can be pushed by the looping pool in vertebrate cells is an interesting open question that would be best addressed using a combination of genomics, biochemistry, and polymer modelling approaches. We refer to this point in the main text as an interesting avenue of future studies.

The discussion regarding other species is not that convincing. Freeman 2000 supports segregation of rDNA by condensin but doesn't show cohesin-mediated segregation (nor resolution, unless I'm mistaken). In general, I am not sure about papers demonstrating that cohesin contribute to segregation, though they do compact G2 chromosomes (Lazar 2017, Schalbeter 2017). Condensin was shown to actively contribute to segregation through the formation of loops and possibly removal of entanglements (Lazar 2017, Paul 2018) and potentially also play a role in decatenation of DNA (eg Dyson 2021).
Also, I am not sure that any experiment supports SMC-mediated segregation in archaea (the Palecek 2015 reference shows that SMCs are conserved), though SMCs shape chromosomes in archaea

(Cockram 2021, Takemata 2019). In bacteria, Marbouty 2015 also show SMC-related segregation by HiC and imaging in B subtilis. Perhaps some work regarding segregation in E. coli could also be mentioned if relevant. The discussion could (and should) therefore be enriched by making more specific statements about other domains of life rather than broad generalizations based on a single set of experiments.

We thank the referee for the suggestions how to improve the discussion related to other species. To address these points, we extended the discussion section to give more specific examples of how SMC proteins in other life forms relate to chromosome segregation and to our observations, and provide more comprehensive citations of prior literature.

Referee #3:

The resolution of sister chromatids is a fundamental requirement for faithful chromosome segregation in higher eukaryotes. Several studies focused on the resolution mechanisms during early mitosis, but whether and how sister chromatids are resolved before mitotic entry is underexplored.

In this exciting manuscript, Batty and co-workers provide compelling evidence that cohesin complexes can resolve sister chromatids to a considerable extent before mitotic entry. Distinct SMC complexes are considered critical to different aspects of genome organisation. However, they all share some biochemical activities, which raises the intriguing question of how the functional divergence is brought about in vivo. Here, the authors use an elegant experimental layout, combined with unique approaches to distinguish genetically identical sister chromatids, to demonstrate that cohesin complexes can resolve DNA molecules to a large extent, even before mitotic entry. They observe that chromatids separation is decreased upon loss of cohesin loop-extrusion (ΔNIPBL) and enhanced in the presence of hyperactive cohesin (ΔWAPL), with condensed and resolved chromatids organised around a single cohesin-axis. Strikingly, enhanced loop extrusion coupled with the loss of cohesion (ΔSororin- ΔWAPL) causes the axes of sister chromatids to split, resulting in G2 chromosomes that highly resemble late prophase chromosomes. Collectively, these findings support that a regulated balance of cohesive and loop-extruding cohesin molecules can resolve sister chromatids before mitosis, an activity previously attributed to condensins and topoisomerases during mitosis.

Overall, the experiments are very well designed, the technologies applied are top-edge, and the data is high quality. The conclusions are novel and challenge current dogmas: 1) They provide evidence that sister chromatid resolution is quite significant before mitotic entry, and 2) they support that this process is primarily mediated by cohesin complexes, previously believed to counteract the resolution process (via cohesive cohesins).

These findings have broad implications for our understanding of sister chromatid resolution and how the activities associated with SMC complexes are regulated in vivo. The concepts that emerge from this study will significantly interest the chromosome biology and genome organization fields. In a broader context, the conclusions are also thought-provoking to anyone interested in the functional divergence of related proteins/complexes.

We do have a few issues that we would like the authors to consider before publication:

Major points

1) The authors established a new protocol for imaging and distinction of sister chromatids. Whereas the presented protocol is an improvement over previously established methods (e.g., Nagasaka et al., 2016), it has the significant caveat that the presence of "two objects" is inferred based on the absence of a signal. This methodology could be problematic if staining is less optimal for unknown reasons. If experimentally possible, the authors should validate their major findings using a strategy where both

are individually labelled. Or at least properly acknowledge this intrinsic caveat of their methodology when comparing their method with previous ones.

We thank the referee for their comment and recognise that this is a limitation of our approach. Previously published methods that individually label both sister chromatids using BrdU as a second nucleotide analogue (e.g. Nagasaka et al, 2016), however, revealed limitations resulting from harsh treatment of cells required to visualise BrdU, which has been shown to change chromosome structure substantially (Beckwith et al, 2022). Moreover, BrdU labels chromatids quite inhomogeneously, such that sister chromatid resolution phenotypes cannot be assessed reliably in interphase cells (e.g., see Nagasaka et al, 2016). We are not aware of any secondary labeling method that would allow visualisation of both sister chromatids in a way that is compatible with the analysis of fine structures as required for our study. Nonetheless, we tried to address this point in the following ways:

Firstly, we explained our approach in more detail and referred to this limitation of our methodology in the text (changes indicated in red):

"To validate our approach, we first assessed the efficiency and specificity of sister chromatid labelling. Due to the semi-conservative nature of DNA replication, F-ara-EdU should be incorporated into each of the newly synthesised DNA strands during S phase when cells are cultured in the presence of F-ara-EdU. Indeed, in the first G2 phase and mitosis after culturing cells in F-ara-EdU, we observed a strong overlap between F-ara-EdU fluorescence and the general DNA dye Hoechst 33342, such that mitotic chromosome arms were labelled on both sister chromatids (Fig. 1b, two-sister labelled; Fig. EV1b). Mitotic cells that had progressed through an additional S phase in the absence of F-ara-EdU, such that the newly replicated strands did not contain F-ara-EdU, had chromosome arms labelled only on a single sister (Fig. 1b, one-sister labelled; Fig. EV1c). Thus, our procedure specifically and completely labels one sister chromatid per chromosome."

"To quantify the resolution of sister chromatids we developed an automated analysis pipeline to calculate the pixel-wise correlation between F-ara-EdU fluorescence and Hoechst 33342 (Fig. EV1d) (see Materials and Methods for details). We reasoned that although our approach allows specific labelling of only a single sister chromatid, by measuring the reduction in correlation between F-ara-EdU and Hoechst in cells labelled on one sister chromatid compared to reference cells labelled on both sisters, we would be able to quantitatively assess changes in sister chromatid resolution in both interphase and mitotic cells. In the first mitosis after labelling, where a high degree of colocalisation between Hoechst and F-ara-EdU is expected, the Spearman correlation coefficient was close to 1, demonstrating homogeneous and complete labeling of both sister chromatids. In the second mitosis, after cells had progressed through one S phase in the absence of F-ara-EdU, the Spearman correlation coefficient dropped to 0.5, as only the F-ara-EdU labelled chromatid colocalised with the Hoechst counterstain, indicating that sister chromatids were highly resolved (Fig. EV1e-g). Based on these two reference conditions, we calculated a normalised sister chromatid separation score (Fig. 1d, e; Fig. EV1h), thereby allowing investigation of sister chromatid resolution under various experimental conditions."

Secondly, in each round of experiments that we performed, a two-sister labelling control was performed to validate that the staining procedure gave the expected results. As validation for our staining procedure, we therefore added additional images and quantifications of the separation score for two-sister labelled prometaphase and G2 cells for all conditions shown in Fig. 1 (see Fig. EV1h, i, EV3g, h, EV4g, h).

2) The analysis lacks a reference point to what would be "no resolution" in interphase chromatin so that the significance of the extent of the reduction observed upon NIBL depletion can be better understood. E.g., use top2 depletion or ICRF treatment to estimate what would be the "unresolved" state. Comparing

it to the prometaphase values upon depletion of SMC4 may not be the best reference, as the state of chromosome compaction may impact the quantification output.

We thank the referee for their suggestion to include a reference condition where no resolution occurs in order to better evaluate the NIPBL depletion phenotype in G2 cells.

To address this point, we have included additional datasets for two-sister labelled prometaphase and G2 cells, for wild type, ΔSMC4, and ΔNIPBL cells, as reference conditions where no resolution is expected, to give a baseline to which the one-sister labelled cells can be compared. We find this a more appropriate control than inhibition/depletion of Topoisomerase II, as prior work showed that sister chromatids still resolve to a certain extent after inhibition/depletion of TopoII, presumably through the activity of condensins (Nagasaka et al; 2016).

3) It remains untested how much of the enhanced cohesin activity in G2 could compensate for the "canonical resolution machinery" in mitosis. The authors performed the proper experiment to address this issue (EV5c) but restricted their analysis to G2 cells. What happens in mitosis in cells double depleted for SMC4 and WAPL? Can WAPL depletion restore the absence of resolution observed by metaphase in the absence of SMC4 (even if around a "single cohesin-axis")? It would further validate the author's claim of extensive DNA resolution (to the maximal level required) if it does. If not, it would raise interesting questions on how mitosis may bring about special requirements for chromatid resolution. Either result would be interesting. The same could be asked for the WAPL+Sor depletion (although performing the triple depletion may be more experimentally demanding). Substantiating how much of their findings in G2 can rescue condensin-mediated mitotic resolution would significantly improve their striking observations.

We thank the referee for their suggestion to investigate the extent to which cohesin can promote sister chromatid resolution in condensin-depleted cells not only in G2 cells, but also in mitosis. To address this point, we performed an experiment where we let cells progress through S and G2 phase in the absence of condensins, depleted WAPL in G2 to increase cohesin's looping processivity, before releasing cells from G2 into mitosis where they were arrested in prometaphase (Fig. 4a, b). We found that although thread-shaped structures can form to a certain extent, chromosome morphology was irregular under these conditions and the sister chromatid separation score was reduced by approximately one third compared to cells where WAPL alone was depleted. Additional co-depletion of Sororin also increased the separation score, as in G2 cells, but chromosome structure was still perturbed. Thus, cohesin retention on mitotic chromosomes can to some extent promote sister chromatid resolution in mitotic cells lacking condensins, but chromosomes do not resolve to their full extent.

That retention of cohesin on condensin-depleted mitotic chromosomes results in irregular chromosome morphology and reduced sister chromatid resolution compared to control cells demonstrates that condensin confers a key functionality in mitosis that cohesin cannot replace. In the main text and discussion, we speculate that the cohesin retained on mitotic chromosomes might no longer able to actively extrude chromatin loops, as NIPBL, which has been shown to be required for cohesin looping both in vitro and in cells, dissociates from chromosomes in mitotic prophase. This could explain the perturbed chromatin structure and inability of cohesin to resolve sister chromatids to their full extent in condensin depleted mitotic chromosomes. Consistent with such a hypothesis, we found that acute depletion of condensins in prometaphase cells that had already resolved their chromosomes resulted in increased sister chromatid intermixing and reduced sister chromatid resolution over time (Fig. 4c-e), consistent with continuous loop extrusion being required both to resolve and maintain resolved sister chromatids.

The new data and quantification are presented in the main text and the new figure.

Minor issues:

1) Although the rationale is very well designed for what the authors wish to test, each figure presents experiments in parallel that were performed differently. For example, in Fig. 1, depletion of NIBL is timed to after S-phase (a must to allow for cohesion establishment), but SMC4 depletion occurs before S-phase. The differences in experimental layout do not preclude author's conclusions (on the contrary) but we would suggest that each figure includes a small scheme so that each experimental layout for each condition is more readily identified. This would actually do better justice to the clever experimental designs employed throughout the manuscript.

We agree that illustrating the experimental schedules facilitates understanding our data. We have provided schematical illustrations of the synchronisation and perturbations in extended data figure panels, which we now reference in the respective main figure legends. This will make it easier to determine the synchronisation protocol and protein depletion regime used for each experimental condition. We also added a brief explanation of the rationale for the timing of protein depletion to the relevant figure legends.

2) The double-depleted WAPL-Sororin cells display sister chromatid axis separation. From the images in Fig2, it looks like the axes overlap in some regions. Do the authors know if those regions are cohered and, if so, whether they coincide with the centromeres? It may be interesting to add a comment on this and how these regions could be brought about (cohesin protection will certainly be different in interphase).

We thank the referee for pointing out that there are some regions where sister chromatid axes do not appear to be split in WAPL/Sororin depleted cells. To evaluate the extent to which cohesin axes split upon Sororin depletion in WAPL depleted G2 cells, we quantified the length of sister chromatid segments with split or non-split axes, finding that 86 ± 3.1 % of cohesin axes are split in WAPL/Sororin depleted cells, compared to 91 ± 4.1 % of condensin axes in late prophase (Fig. EV7a). We further quantified the depletion efficiency using quantitative immunofluorescence, finding nuclear Sororin levels in siRNA-transfected cells to be 16 ± 5.8 % of the levels observed in control cells (Fig. EV7b, c). We speculate that the residual Sororin protein provides cohesion at some small regions between sister chromatids, potentially at centromeric regions, and now mention this in the main text.

3) "Thus, in the presence of arm cohesion, DNA loop extrusion promotes a highly ASYMMETRIC displacement of sister chromatid DNA away from a central cohesin axis." From Fig3b, the displacement of sister chromatids looks rather symmetrical, as the two peaks localise at a similar distance from the central cohesin axis. We suggest rephrasing the sentence to improve clarity.

We thank the referee for raising this ambiguity and modified the text for clarification. We used the term asymmetry when relating the organisation of each sister chromatid's DNA to the cohesin axis. In unperturbed prometaphase chromosomes, the DNA of each sister chromatid is organised in a radially symmetrical distribution relative to the condensin axis of each sister chromatid. In WAPL depleted cells, the DNA of each sister chromatid is displaced outwards relative to a single cohesin axis that localises at the interface between the sister chromatids. We revised the main text as follows:

"This showed an outward-facing displacement of sister DNA relative to a single axis of cohesin in WAPL depleted G2 cells, in contrast to a symmetrical organisation of sister DNA around each of the split condensin or cohesin axes in unperturbed prophase cells or WAPL/Sororin depleted G2 cells (Fig. 3e). Thus, in the presence of arm cohesion, DNA loop extrusion promotes displacement of sister chromatid DNA away from a central cohesin axis, whereas under conditions of low arm cohesion, sister chromatids form two separate radially symmetrical bottlebrushes around split SMC axes."

4) The authors state that depletion of WAPL (hyperactive cohesin) is more robust in promoting resolution than Sororin depletion (removing cohesin). However, the graph in Fig. 2d does not present statistical analysis between these two conditions.

We thank the reviewer for pointing out that statistical testing was missing in this experiment. We have analysed these two perturbations using an appropriate statistical test (Mann Whitney U test) to support our conclusions and updated the requisite figure panel and legend.

5) The authors use Mann Whitney U test for comparative analysis throughout the manuscript, which is ideal to compare 2 groups. Using a non-parametric one-way ANOVA equivalent (e.g, Kruskal-Wallis) would be possibly more appropriate for multiple comparisons between 3 or more experimental conditions.

We thank the referee for their suggestion. In the manuscript we exclusively perform pairwise statistical analysis to compare two groups at a time and as such the Mann Whitney U is an appropriate test to use.

6) In the caption of Fig3, the last line reports: "Yellow boxes indicate inset regions". There are no yellow boxes in the figure.

We have removed the reference to yellow boxes in this figure legend.

7) In page 7, the last sentence of the "Genomic range of sister chromatids resolution" section says: " [...] chromatin loop extrusion is a fundamental mechanism by which genomes are segregated." The sentence can be misinterpreted as segregation is often referred to the poleward movement of chromatids during anaphase. Maybe using "separated" or "resolved" instead would be more appropriate.

We thank the referee for their suggestion and have changed the wording to remove possible ambiguity relating to the word segregation, which is often used in the context of the mitotic spindle. The changes are highlighted in red below.

"In contrast, increasing the DNA loop-extruding processivity of cohesin is sufficient to resolve sister chromatids over large genomic distances, even in the absence of mitotic activities, supporting that chromatin loop extrusion is a fundamental mechanism by which genomes are propagated to daughters."

Dr. Paul Batty
Institute of Molecular Biotechnology of the Austrian Academy of Sciences (IMBA)
Dr. Bohrgasse 3
Vienna 1030
Austria

23rd May 2023

Re: EMBOJ-2023-113475R
Cohesin-mediated DNA loop extrusion resolves sister chromatids in G2 phase

Dear Dr. Batty,

Thank you again for submitting your revised manuscript for our consideration. Referees 2 and 3 have now assessed it once more, and are mostly satisfied with the revisions and improvements of the manuscript. As you will see from the comments below, they only list a few minor points that would still require modification, and I would herewith like to invite you to incorporate them in a final round of minor revision.

In addition, there are also still a few editorial points that would need attention:

- In the Data Availability section, please make sure to include a direct URL allowing to access the referenced datasets (via their accession number) from the relevant databases (such as GEO). Please also ensure that datasets will become rapidly released to the public at this point.

- There are currently 12 "Expanded View" figures, but we only allow for 5, max. 6 of these "second-level" figures - with additional ones needing to go into the Appendix (i.e. "third level"). Therefore, I would appreciate if you would go through all Expanded View figures and selected the most important ones to remain "EV figures", while converting the remainder to "Appendix Figures S1/2/3..." - making sure to adjust all call-outs throughout the manuscript accordingly, and moving them TOGETHER WITH their legends into the Appendix PDF. Please, make sure to list them in the Appendix Table of Contents just like the Appendix Tables. Also, please head off the Appendix with a brief reference to the main article, such as "Appendix for: Cohesin-mediated DNA loop extrusion ...".

I am therefore returning the manuscript to you for a final round of minor revision, to allow you to make these adjustments and upload all modified files. Once we will have received them, we should be ready to swiftly proceed with formal acceptance and production of the manuscript.

With kind regards,

Hartmut Vodermaier

Hartmut Vodermaier, PhD
Senior Editor, The EMBO Journal
h.vodermaier@embojournal.org

*** PLEASE NOTE: All revised manuscript are subject to initial checks for completeness and adherence to our formatting guidelines. Revisions may be returned to the authors and delayed in their editorial re-evaluation if they fail to comply to the following requirements (see also our Guide to Authors for further information):

1) Every manuscript requires a Data Availability section (even if only stating that no deposited datasets are included). Primary datasets or computer code produced in the current study have to be deposited in appropriate public repositories prior to resubmission, and reviewer access details provided in case that public access is not yet allowed. Further information: embopress.org/page/journal/14602075/authorguide#dataavailability

2) Each figure legend must specify
- size of the scale bars that are mandatory for all micrograph panels
- the statistical test used to generate error bars and P-values
- the type error bars (e.g., S.E.M., S.D.)
- the number (n) and nature (biological or technical replicate) of independent experiments underlying each data point
- Figures may not include error bars for experiments with n<3; scatter plots showing individual data points should be used

instead.

3) Revised manuscript text (including main tables, and figure legends for main and EV figures) has to be submitted as editable text file (e.g., .docx format). We encourage highlighting of changes (e.g., via text color) for the referees' reference.

4) Each main and each Expanded View (EV) figure should be uploaded as individual production-quality files (preferably in .eps, .tif, .jpg formats). For suggestions on figure preparation/layout, please refer to our Figure Preparation Guidelines: http://bit.ly/EMBOPressFigurePreparationGuideline

5) Point-by-point response letters should include the original referee comments in full together with your detailed responses to them (and to specific editor requests if applicable), and also be uploaded as editable (e.g., .docx) text files.

6) Please complete our Author Checklist, and make sure that information entered into the checklist is also reflected in the manuscript; the checklist will be available to readers as part of the Review Process File. A download link is found at the top of our Guide to Authors: embopress.org/page/journal/14602075/authorguide

7) All authors listed as (co-)corresponding need to deposit, in their respective author profiles in our submission system, a unique ORCiD identifier linked to their name. Please see our Guide to Authors for detailed instructions.

8) Please note that supplementary information at EMBO Press has been superseded by the 'Expanded View' for inclusion of additional figures, tables, movies or datasets; with up to five EV Figures being typeset and directly accessible in the HTML version of the article. For details and guidance, please refer to: embopress.org/page/journal/14602075/authorguide#expandedview

9) Digital image enhancement is acceptable practice, as long as it accurately represents the original data and conforms to community standards. If a figure has been subjected to significant electronic manipulation, this must be clearly noted in the figure legend and/or the 'Materials and Methods' section. The editors reserve the right to request original versions of figures and the original images that were used to assemble the figure. Finally, we generally encourage uploading of numerical as well as gel/blot image source data; for details see: embopress.org/page/journal/14602075/authorguide#sourcedata

At EMBO Press, we ask authors to provide source data for the main manuscript figures. Our source data coordinator will contact you to discuss which figure panels we would need source data for and will also provide you with helpful tips on how to upload and organize the files.

Further information is available in our Guide For Authors:
https://www.embopress.org/page/journal/14602075/authorguide

In the interest of ensuring the conceptual advance provided by the work, we recommend submitting a revision within 3 months (21st Aug 2023). Please discuss the revision progress ahead of this time with the editor if you require more time to complete the revisions. Use the link below to submit your revision:

Link Not Available

Referee #2:

The authors have taken into account the comments made in this revised version of their work. They discuss the suggested points, i.e., cohesion conservation upon wapl depletion and cohesin conservation across life domains.
The authors omitted to cite Kleckner's work on sister chromatid decatenation by increasing loop size, which I really think they need to cite at the very least for the beautiful resolved sisters images (Chu et al (2020, 2022). Ideally, they also should discuss in a couple of sentences the effect of loop extrusion on catenation in G2 in light of these works and theirs.
With this minor addition, I consider the revised work to meet most of the reviewers' comments and recommend publication.

Referee #3:

I would like to congratulate the authors on their work in revising the manuscript. Most of my concerns have been appropriately addressed, and I can now fully recommend the publication of this interesting and thought-provoking manuscript.

I just have two minor issues regarding this version:

1) The authors present new data on acute condensin's depletion to show that resolution of sister chromatids is reverted even after their prior resolution. The authors conclude that "Thus, active loop extrusion is not only required to establish sister chromatid resolution, but also to maintain it." (page 4) and suggest "Like Topoisomerase II (Nielsen et al, 2020), active loop extrusion might therefore be required both for the formation and maintenance of resolved sister chromatids during mitosis." (page 6)
These statements should be followed by the appropriate references that first observed this requirement (Sen et al Mol Cell 2016 and Pikadlo et al, eLife 2017)

2) There are some issues with references that are found in the reference list but not in the main text:
Piskadlo et al 2017 (linked with my previous comment)
Coelho et al 2003
Wang et al 2015

We again thank the referees for their interest in our work and for helping us to improve our manuscript and are pleased that they recommend publication following the completion of minor changes. We have addressed each of the referee and editorial comments in a point-by-point response below.

Editorial points

- In the Data Availability section, please make sure to include a direct URL allowing to access the referenced datasets (via their accession number) from the relevant databases (such as GEO). Please also ensure that datasets will become rapidly released to the public at this point.

We have provided a direct URL and GEO accession number for the scsHi-C datasets generated in this manuscript in the 'Data Availability' section. The Hi-C data is now publicly available at GEO.

We have also provided a direct URL to the code used to analyse sequencing and microscopy data in this manuscript in the 'Data Availability' section. The code has been deposited to GitHub.

- There are currently 12 "Expanded View" figures, but we only allow for 5, max. 6 of these "second-level" figures - with additional ones needing to go into the Appendix (i.e. "third level").

Therefore, I would appreciate if you would go through all Expanded View figures and selected the most important ones to remain "EV figures", while converting the remainder to "Appendix Figures S1/2/3..." - making sure to adjust all call-outs throughout the manuscript accordingly, and moving them TOGETHER WITH their legends into the Appendix PDF.

Please, make sure to list them in the Appendix Table of Contents just like the Appendix Tables.

Also, please head off the Appendix with a brief reference to the main article, such as "Appendix for: Cohesin-mediated DNA loop extrusion ...".

We have performed these editorial changes as requested. We have reduced the number of Extended Version figures to 6 and moved the additional figures to the Appendix. We have also adjusted the call out of the figure panels in the main text accordingly and formatted the Appendix as requested.

Referee #2:

The authors have taken into account the comments made in this revised version of their work. They discuss the suggested points, i.e., cohesion conservation upon wapl depletion and cohesin conservation across life domains.

The authors omitted to cite Kleckner's work on sister chromatid decatenation by increasing loop size, which I really think they need to cite at the very least for the beautiful resolved sisters images (Chu et al (2020, 2022). Ideally, they also should discuss in a couple of sentences the effect of loop extrusion on catenation in G2 in light of these works and theirs.
With this minor addition, I consider the revised work to meet most of the reviewers' comments and recommend publication.

We thank the referee for their interest in our work and for their positive evaluation.

We have now cited Chu et al, 2020 in the introduction, and additionally have expanded upon the effect of loop extrusion, and its potential interplay with decatenation/catenation of sister chromatids in the discussion.

Referee #3:

I would like to congratulate the authors on their work in revising the manuscript. Most of my concerns have been appropriately addressed, and I can now fully recommend the publication of this interesting and thought-provoking manuscript.

We thank the referee for their interest in our work for and their positive evaluation.

I just have two minor issues regarding this version:

1) The authors present new data on acute condensin's depletion to show that resolution of sister chromatids is reverted even after their prior resolution. The authors conclude that "Thus, active loop extrusion is not only required to establish sister chromatid resolution, but also to maintain it." (page 4) and suggest "Like Topoisomerase II (Nielsen et al, 2020), active loop extrusion might therefore be required both for the formation and maintenance of resolved sister chromatids during mitosis." (page 6)
These statements should be followed by the appropriate references that first observed this requirement (Sen et al Mol Cell 2016 and Piskadlo et al, eLife 2017)

We thank the reviewer for their help in citing comprehensively and have added these references to the appropriate section.

2) There are some issues with references that are found in the reference list but not in the main text:
Piskadlo et al 2017 (linked with my previous comment)
Coelho et al 2003
Wang et al 2015

We thank the reviewer for noticing this discrepancy and have updated the reference list accordingly to remove citations that are not mentioned in the main text.

Dr. Daniel W. Gerlich
Institute of Molecular Biotechnology of the Austrian Academy of Sciences (IMBA)
-
Dr. Bohrgasse 3
Vienna 1030
Austria

14th Jun 2023

Re: EMBOJ-2023-113475R1
Cohesin-mediated DNA loop extrusion resolves sister chromatids in G2 phase

Dear Daniel,

Thank you for submitting your final revised manuscript for our consideration. I am pleased to inform you that we have now accepted it for publication in The EMBO Journal.

Your article will be processed for publication in The EMBO Journal by EMBO Press and Wiley, who will contact you with further information regarding production/publication procedures and license requirements. You will also be provided with page proofs after copy-editing and typesetting of main manuscript and expanded view figure files.

Should you be planning a Press Release on your article, please get in contact with embojournal@wiley.com as early as possible, in order to coordinate publication and release dates.

Thank you again for this contribution to The EMBO Journal and congratulations on a successful publication! Please consider us again in the future for your most exciting work.

With kind regards,

Hartmut

Hartmut Vodermaier, PhD
Senior Editor, The EMBO Journal
h.vodermaier@embojournal.org

EMBO Press Author Checklist

Corresponding Author Name: Daniel W. Gerlich, Paul Batty	
Journal Submitted to: The EMBO Journal	
Manuscript Number: EMBOJ-2023-113475	

USEFUL LINKS FOR COMPLETING THIS FORM
The EMBO Journal - Author Guidelines
EMBO Reports - Author Guidelines
Molecular Systems Biology - Author Guidelines
EMBO Molecular Medicine - Author Guidelines

Reporting Checklist for Life Science Articles (updated January
This checklist is adapted from Materials Design Analysis Reporting (MDAR) Checklist for Authors. MDAR establishes a minimum set of requirements in transparent reporting in the life sciences (see Statement of Task: 10.31222/osf.io/9sm4x). Please follow the journal's guidelines in preparing your
Please note that a copy of this checklist will be published alongside your article.

Abridged guidelines for figures
1. Data
The data shown in figures should satisfy the following conditions:
- ➞ the data were obtained and processed according to the field's best practice and are presented to reflect the results of the experiments in an accurate and unbiased manner.
- ➞ ideally, figure panels should include only measurements that are directly comparable to each other and obtained with the same assay.
- ➞ plots include clearly labeled error bars for independent experiments and sample sizes. Unless justified, error bars should not be shown for technical
- ➞ if n<5, the individual data points from each experiment should be plotted. Any statistical test employed should be justified.
- ➞ Source Data should be included to report the data underlying figures according to the guidelines set out in the authorship guidelines on Data

2. Captions
Each figure caption should contain the following information, for each panel where they are relevant:
- ➞ a specification of the experimental system investigated (eg cell line, species name).
- ➞ the assay(s) and method(s) used to carry out the reported observations and measurements.
- ➞ an explicit mention of the biological and chemical entity(ies) that are being measured.
- ➞ an explicit mention of the biological and chemical entity(ies) that are altered/varied/perturbed in a controlled manner.
- ➞ the exact sample size (n) for each experimental group/condition, given as a number, not a range;
- ➞ a description of the sample collection allowing the reader to understand whether the samples represent technical or biological replicates (including how many animals, litters, cultures, etc.).
- ➞ a statement of how many times the experiment shown was independently replicated in the laboratory.
- ➞ definitions of statistical methods and measures:
  - common tests, such as t-test (please specify whether paired vs. unpaired), simple χ2 tests, Wilcoxon and Mann-Whitney tests, can be unambiguously identified by name only, but more complex techniques should be described in the methods section;
  - are tests one-sided or two-sided?
  - are there adjustments for multiple comparisons?
  - exact statistical test results, e.g., P values = x but not P values < x;
  - definition of 'center values' as median or average;
  - definition of error bars as s.d. or s.e.m.

Please complete ALL of the questions below.
Select "Not Applicable" only when the requested information is not relevant for your study.

Materials

Newly Created Materials	Information included in the manuscript?	In which section is the information available? (Reagents and Tools Table, Materials and Methods, Figures, Data Availability Section)
New materials and reagents need to be available; do any restrictions apply?	Not Applicable	

Antibodies	Information included in the manuscript?	In which section is the information available? (Reagents and Tools Table, Materials and Methods, Figures, Data Availability Section)
For **antibodies** provide the following information:<br>- Commercial antibodies: RRID (if possible) or supplier name, catalogue number and or/clone number<br>- Non-commercial: RRID or citation	Yes	Materials and Methods

DNA and RNA sequences	Information included in the manuscript?	In which section is the information available? (Reagents and Tools Table, Materials and Methods, Figures, Data Availability Section)
**Short novel DNA or RNA including primers, probes**: provide the sequences.	Yes	Materials and Methods

Cell materials	Information included in the manuscript?	In which section is the information available? (Reagents and Tools Table, Materials and Methods, Figures, Data Availability Section)
**Cell lines:** Provide species information, strain. Provide accession number in repository **OR** supplier name, catalog number, clone number, and/**OR** RRID.	Yes	Materials and Methods
**Primary cultures:** Provide species, strain, sex of origin, genetic modification status.	Not Applicable	
Report if the cell lines were recently **authenticated** (e.g., by STR profiling) and tested for mycoplasma contamination.	Yes	Materials and Methods

Experimental animals	Information included in the manuscript?	In which section is the information available? (Reagents and Tools Table, Materials and Methods, Figures, Data Availability Section)
**Laboratory animals or Model organisms:** Provide species, strain, sex, age, genetic modification status. Provide accession number in repository **OR** supplier name, catalog number, clone number, **OR** RRID.	Not Applicable	
**Animal observed in or captured from the field:** Provide species, sex, and age where possible.	Not Applicable	
Please detail **housing and husbandry conditions**.	Not Applicable	

Plants and microbes	Information included in the manuscript?	In which section is the information available? (Reagents and Tools Table, Materials and Methods, Figures, Data Availability Section)
**Plants:** provide species and strain, ecotype and cultivar where relevant, unique accession number if available, and source (including location for collected wild specimens).	Not Applicable	
**Microbes:** provide species and strain, unique accession number if available, and source.	Not Applicable	

Human research participants	Information included in the manuscript?	In which section is the information available? (Reagents and Tools Table, Materials and Methods, Figures, Data Availability Section)
If collected and within the bounds of privacy constraints report on age, sex and gender or ethnicity for all study participants.	Not Applicable	

Core facilities	Information included in the manuscript?	In which section is the information available? (Reagents and Tools Table, Materials and Methods, Figures, Data Availability Section)
If your work benefited from core facilities, was their service mentioned in the acknowledgments section?	Yes	Materials and Methods

Design

Study protocol	Information included in the manuscript?	In which section is the information available? (Reagents and Tools Table, Materials and Methods, Figures, Data Availability Section)
If study protocol has been **pre-registered, provide DOI in the manuscript**. For clinical trials, provide the trial registration number **OR** cite DOI.	Not Applicable	
Report the **clinical trial registration number** (at ClinicalTrials.gov or equivalent), where applicable.	Not Applicable	

Laboratory protocol	Information included in the manuscript?	In which section is the information available? (Reagents and Tools Table, Materials and Methods, Figures, Data Availability Section)
Provide DOI OR other citation details if **external detailed step-by-step protocols** are available.	Yes	Materials and Methods

Experimental study design and statistics	Information included in the manuscript?	In which section is the information available? (Reagents and Tools Table, Materials and Methods, Figures, Data Availability Section)
Include a statement about **sample size** estimate even if no statistical methods were used.	Yes	Materials and Methods
Were any steps taken to minimize the effects of subjective bias when allocating animals/samples to treatment (e.g. **randomization procedure**)? If yes, have they been described?	Yes	Materials and Methods
Include a statement about **blinding** even if no blinding was done.	Yes	Materials and Methods
Describe **inclusion/exclusion criteria** if samples or animals were excluded from the analysis. Were the criteria pre-established?<br><br>If sample or data points were omitted from analysis, report if this was due to attrition or intentional exclusion and provide justification.	Yes	Materials and Methods
For every figure, are **statistical tests** justified as appropriate? Do the data meet the assumptions of the tests (e.g., normal distribution)? Describe any methods used to assess it. Is there an estimate of variation within each group of data? Is the variance similar between the groups that are being statistically compared?	Yes	Materials and Methods

Sample definition and in-laboratory replication	Information included in the manuscript?	In which section is the information available? (Reagents and Tools Table, Materials and Methods, Figures, Data Availability Section)
In the figure legends: state number of times the experiment was **replicated** in laboratory.	Yes	Figures
In the figure legends: define whether data describe **technical or biological replicates**.	Yes	Figures

Ethics

Ethics	Information included in the manuscript?	In which section is the information available? (Reagents and Tools Table, Materials and Methods, Figures, Data Availability Section)
Studies involving **human participants**: State details of **authority granting ethics approval** (IRB or equivalent committee(s), provide reference number for approval.	Not Applicable	
Studies involving **human participants**: Include a statement confirming that **informed consent** was obtained from all subjects and that the experiments conformed to the principles set out in the WMA Declaration of Helsinki and the Department of Health and Human Services Belmont Report.	Not Applicable	
Studies involving **human participants:** For publication of **patient photos**, include a statement confirming that consent to publish was obtained.	Not Applicable	
Studies involving experimental **animals**: State details of **authority granting ethics approval** (IRB or equivalent committee(s), provide reference number for approval. Include a statement of compliance with ethical regulations.	Not Applicable	
Studies involving **specimen and field samples:** State if relevant **permits** obtained, provide details of authority approving study; if none were required, explain why.	Not Applicable	

Dual Use Research of Concern (DURC)	Information included in the manuscript?	In which section is the information available? (Reagents and Tools Table, Materials and Methods, Figures, Data Availability Section)
Could your study fall under dual use research restrictions? Please check biosecurity documents and list of **select agents and toxins** (CDC): https://www.selectagents.gov/sat/list.htm	Not Applicable	
If you used a select agent, is the security level of the lab appropriate and reported in the manuscript?	Not Applicable	
If a study is subject to dual use research of concern regulations, is the name of the **authority granting approval and reference number** for the regulatory approval provided in the manuscript?	Not Applicable	

Reporting

The MDAR framework recommends adoption of discipline-specific guidelines, established and endorsed through community initiatives. Journals have their own policy about requiring specific guidelines and recommendations to complement MDAR.

Adherence to community standards	Information included in the manuscript?	In which section is the information available? (Reagents and Tools Table, Materials and Methods, Figures, Data Availability Section)
State if relevant guidelines or checklists (e.g., **ICMJE, MIBBI, ARRIVE, PRISMA**) have been followed or provided.	Not Applicable	
For **tumor marker prognostic studies,** we recommend that you follow the **REMARK** reporting guidelines (see link list at top right). See author guidelines, under 'Reporting Guidelines'. Please confirm you have followed these guidelines.	Not Applicable	
For **phase II and III randomized controlled trials**, please refer to the **CONSORT** flow diagram (see link list at top right) and submit the CONSORT checklist (see link list at top right) with your submission. See author guidelines, under 'Reporting Guidelines'. Please confirm you have submitted this list.	Not Applicable	

Data Availability

Data availability	Information included in the manuscript?	In which section is the information available? (Reagents and Tools Table, Materials and Methods, Figures, Data Availability Section)
Have **primary datasets** been deposited according to the journal's guidelines (see 'Data Deposition' section) and the respective accession numbers provided in the Data Availability Section?	Yes	Materials and Methods
Were **human clinical and genomic datasets** deposited in a public access-controlled repository in accordance to ethical obligations to the patients and to the applicable consent agreement?	Yes	Materials and Methods
Are **computational models** that are central and integral to a study available without restrictions in a machine-readable form? Were the relevant accession numbers or links provided?	Not Applicable	
If publicly available data were reused, provide the respective **data citations in the reference list**.	Yes	Materials and Methods
